# Differential clonal evolution in oesophageal cancers in response to neo-adjuvant chemotherapy

**DOI:** 10.1038/ncomms11111

**Published:** 2016-04-05

**Authors:** John M. Findlay, Francesc Castro-Giner, Seiko Makino, Emily Rayner, Christiana Kartsonaki, William Cross, Michal Kovac, Danny Ulahannan, Claire Palles, Richard S. Gillies, Thomas P. MacGregor, David Church, Nicholas D. Maynard, Francesca Buffa, Jean-Baptiste Cazier, Trevor A. Graham, Lai-Mun Wang, Ricky A. Sharma, Mark Middleton, Ian Tomlinson

**Affiliations:** 1 Molecular and Population Genetics Laboratory, Oxford Centre for Cancer Gene Research, Wellcome Trust Centre for Human Genetics, University of Oxford, Roosevelt Drive, Oxford OX3 7BN, UK; 2 Oxford Oesophagogastric Centre, Churchill Hospital, Oxford University Hospitals NHS Trust, Oxford OX3 7LJ, UK; 3 Genomic Medicine Theme, Oxford NIHR Biomedical Research Centre, Wellcome Trust Centre for Human Genetics, University of Oxford, Roosevelt Drive, Oxford OX3 7BN, UK; 4 Department of Oncology, Old Road Campus Research Building, Oxford OX3 7DQ, UK; 5 Oxford Institute for Radiation Oncology, Old Road Campus Research Building, Oxford OX3 7DQ, UK; 6 Evolution and Cancer Laboratory, Bart's Cancer Institute, Queen Mary University of London, Charterhouse Square, London EC1M 6BQ, UK; 7 Centre for Computational Biology, Haworth Building, and School of Cancer Sciences, College of Medical and Dental Sciences, University of Birmingham, Edgbaston, Birmingham B15 2TT, UK; 8 Department of Cellular Pathology, John Radcliffe Hospital, Oxford University Hospitals NHS Trust, Oxford OX3 9DU, UK; 9 Cancer Theme, Oxford NIHR Comprehensive Biomedical Research Centre, Old Road Campus Research Building, Roosevelt Drive, Oxford OX3 7DQ, UK

## Abstract

How chemotherapy affects carcinoma genomes is largely unknown. Here we report whole-exome and deep sequencing of 30 paired oesophageal adenocarcinomas sampled before and after neo-adjuvant chemotherapy. Most, but not all, good responders pass through genetic bottlenecks, a feature associated with higher mutation burden pre-treatment. Some poor responders pass through bottlenecks, but re-grow by the time of surgical resection, suggesting a missed therapeutic opportunity. Cancers often show major changes in driver mutation presence or frequency after treatment, owing to outgrowth persistence or loss of sub-clones, copy number changes, polyclonality and/or spatial genetic heterogeneity. Post-therapy mutation spectrum shifts are also common, particularly C>A and TT>CT changes in good responders or bottleneckers. Post-treatment samples may also acquire mutations in known cancer driver genes (for example, *SF3B1*, *TAF1* and *CCND2*) that are absent from the paired pre-treatment sample. Neo-adjuvant chemotherapy can rapidly and profoundly affect the oesophageal adenocarcinoma genome. Monitoring molecular changes during treatment may be clinically useful.

## Abstract (web summary)

Oesophageal adenocarcinoma is often treated with chemotherapy before surgery. Here, the authors sequence cancer samples before and after chemotherapy and examine how the genome changes, focusing on changes in driver gene mutations and differential clonal evolution between good and poor responders.

## Graphical abstract

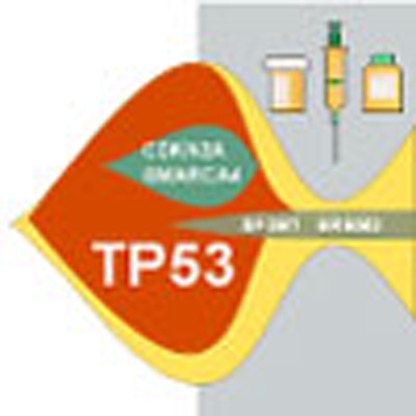

Oesophageal adenocarcinoma (EAC) is routinely treated with neo-adjuvant chemotherapy or chemo-radiotherapy before surgical resection. Clinicopathological response to this therapy is variable and is currently the primary predictor of survival and recurrence after surgery, but overall prognosis remains poor^1^,^2^. It is plausible that some tumours are intrinsically therapy-resistant, while others are partially sensitive or contain a mixed population of sensitive and resistant cells (Supplementary Fig. 1). It appears, however, that most cancers contain at least some resistant cells^3^.

The differences in therapeutic response among tumours might reflect several factors, including tumour genetics or epigenetics. Certain (epi)mutations in the pre-treatment sample might confer almost homogenous resistance or sensitivity to therapy, similar to that seen for *KRAS-*mutant cancers treated with targeted EGFR inhibitors^4^. Alternatively, resistant tumour sub-clones might carry (epi)mutations in resistance genes, although these (epi)mutations may be rare and difficult to detect in the pre-treatment sample. A longstanding example here is methylation of *MLH1,* and the resultant subsequent microsatellite instability, that can occur in ovarian cancers after platinum-based chemotherapy^5^. However, differences in response are not necessarily determined by genetic or epigenetic changes, and other factors such as hypoxia^6^, the cancer stem cell phenotype^7^, and stromal microenvironment^8^,^9^ have been implicated in therapeutic resistance.

The ability to perform repeated sampling with minimal patient risk makes haematological malignancies ideal for studying how neoplastic genomes change over time. Several such studies have shown mutations in specific genes to differ between leukaemia cells at presentation and relapse (for example, refs 10, 11, 12), and more recently, genome-wide sequencing has greatly increased our insights into how leukaemias and lymphomas evolve in patients who relapse following induction of remission with chemotherapy^13^,^14^,^15^,^16^,^17^. These studies have largely shown the lesions at presentation to be monoclonal or oligoclonal. In general, the relapses are clearly genetically related to the presenting clone, but may have reduced complexity and a number of ‘private' mutations, consistent with genetic bottlenecking followed by outgrowth of a clone that has not been killed by the chemotherapy used. These findings have led to speculation as to whether similar genetic phenomena occur during the treatment of solid adult malignancies, particularly cancers with low response rates to chemotherapy.

Solid tumours, such as EAC, differ from ‘liquid tumours' in their cellular and molecular origins, their environments, their clonal structures and dynamics, and their rates of response to non-surgical therapy. Multi-region sampling of primary cancers and metastases has shown varying degrees of branched tumour evolution, including the divergence of metastases from primary cancers at very early stages, parallel or convergent evolution (in which the same driver genes acquire different mutations in different regions of the tumour), and widely varying mutation rates, spectra and signatures (for example, refs 18, 19, 20). Where molecularly targeted therapies have been used, sequential biopsies of solid tumours and/or serial samples of circulating tumour cells or DNA have confirmed that most of the cells carrying the targeted mutation can be killed, but resistance usually develops owing to pre-existing reversion mutations in the targeted protein or a different component of its pathway (for example, refs 21, 22, 23, 24, 25, 26, 27). In studies of chemotherapeutic regimens, small gene sets or panels have shown that the frequencies of specific mutations or molecular phenotypes can change significantly^28^,^29^,^30^,^31^. Recently, studies of gliomas and glioblastomas have begun to examine how the exomes and genomes of solid tumours change following radiotherapy and chemotherapy, with some tumours showing major clonal shifts^32^,^33^. However, brain tumour evolution may be quite different from that of the common cancers, and little is known about how carcinoma genomes alter in response to genotoxic therapy^34^,^35^. Moreover, the use of neo-adjuvant chemotherapy to shrink tumours such as EAC before surgery provides the opportunity to compare the evolution of cancers that respond well and poorly, since response is rarely clinicopathologically complete and the majority of patients still undergo surgery.

In this study, we investigated the effects of therapy on EACs, comparing primary tumours with paired samples after two cycles of neo-adjuvant 5-fluorouracil and oxaliplatin. Our main aim was to examine how chemotherapy affected the architecture of the oesophageal cancer genome in responders compared with non-responders. A subsidiary aim was to identify mutations driving therapeutic resistance or tumour growth in responders after treatment.

## Results

### Overview of patients and sequencing strategy

Oesophageal cancer patients in the study (Table 1; Supplementary Table 1) received two cycles of oxaliplatin-5FU, each lasting 21 days (Methods). Following completion of therapy, restaging was performed by positron emission tomography-computed tomography (PET-CT). In the absence of progression to metastatic and/or unresectable disease, patients underwent attempted surgical resection 4–6 weeks later. Thirty EAC patients were enrolled for molecular analysis, representing 15 cases who had a clinically significant, but incomplete, pathological response to neo-adjuvant therapy (Mandard grade 2 or 3, ‘responders') and 15 with very limited or no response (Mandard grade ≥4, ‘non-responders')^1^,^2^,^36^. Each patient provided two fresh-frozen (FF) cancer samples, one ‘pre-treatment' at endoscopic biopsy and one ‘post-treatment' at surgical resection. Matched, histologically normal oesophageal tissue distinct from the cancer was also obtained before treatment. Additional paired or unpaired formalin-fixed, paraffin-embedded (FFPE) cancer samples were available from a sub-set of 20 patients.

After histological review (Supplementary Fig. 2), the FF sample trios from each patient underwent exome capture and sequencing to median depth of 74 × (range 36–133 × , Supplementary Fig. 3). The median somatic single nucleotide variant (SNV) and small insertion/deletion (indel) burden in the exome and other captured regions was 373 (range 50-6023) in pre-treatment samples, and this reduced to 272 (range 51–7051) in post-treatment samples (*P*<0.001, Wilcoxon test). Two cancers were microsatellite-unstable (microsatellite instability (MSI)+) in both pre-and post-treatment samples and these had very elevated SNV and indel burdens. Further sequencing metrics are shown in Supplementary Fig. 3.

We additionally performed deep sequencing of all available FF samples using the Ampliseq Comprehensive Cancer Panel, which contained most of the putative EAC drivers and a total of 409 genes. Median average read depths of 1,888 × and 1,787 × were achieved in the pre- and post-treatment cancers. The matched normal pre-treatment samples were sequenced at standard depth (median=224 ×). Of 278 SNVs and small indels found by exome sequencing within Comprehensive Cancer Panel genes, 97% were confirmed by Ampliseq, with very strong concordance between variant allele frequencies (VAFs) from the two platforms despite the different sequencing depths (Supplementary Fig. 4).

### The pre-treatment EAC genome

**Figure 1:**
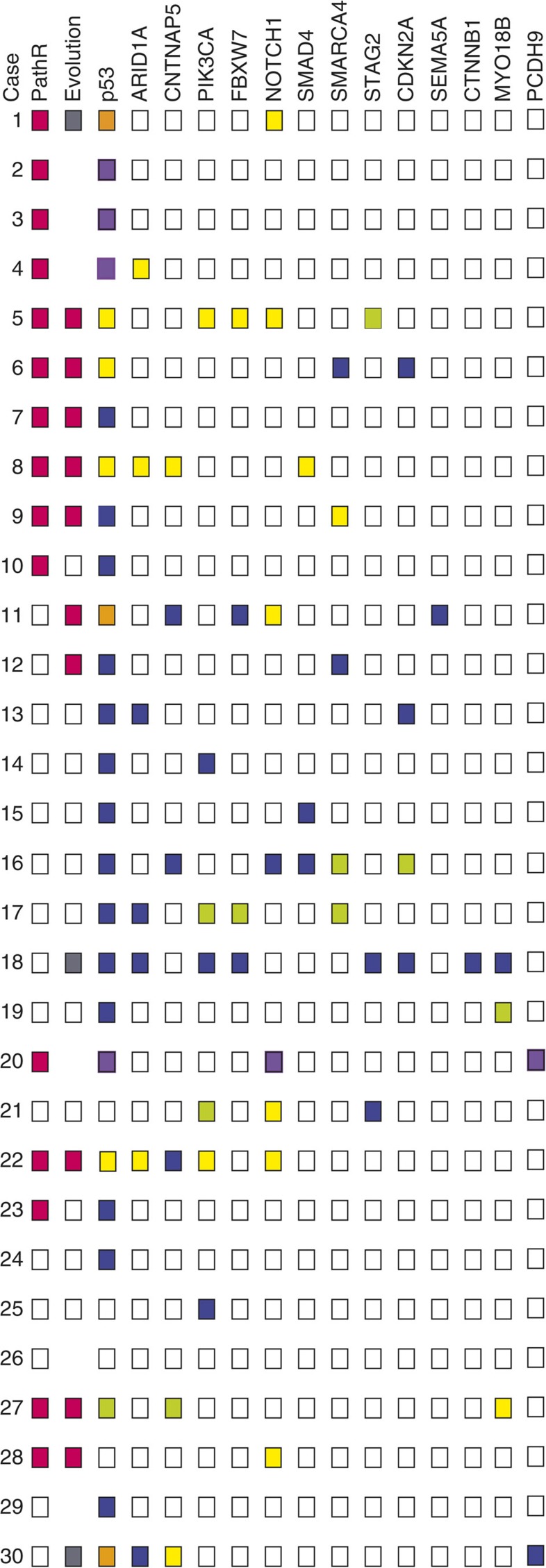
Summary of mutations and other molecular features in each cancer from exome sequencing. PathR: pathological response (pink) or non-response (clear). Evolution: putative bottleneck (pink), polyclonality (grey), no major clonal change (clear) or excluded owing to concerns about tumour purity as detailed in Supplementary Note 1 (absent). Mutant genes: yellow, SNV or small indel mutation in pre-treatment sample only; purple, mutation in pre-treatment sample but post-treatment sample excluded; lime green, mutation in post-treatment sample; blue, mutation present in both pre- and post-treatment sample; clear, no mutation detected; orange, more than one mutation present, with different directions of VAF change. MSI, microsatellite instability.

Exome sequencing showed that nearly all (28/30, 93%) cancers carried potentially functional (non-synonymous, protein-truncating or splice site) somatic SNVs in one or more of the previously postulated EAC driver genes (Fig. 1; Supplementary Table 2)^37^,^38^. *TP53* (p53) was mutated in 23 (77%) of the pre-treatment cancers and all of these changes were protein-truncating or missense mutations previously shown to be pathogenic. Other frequently mutated EAC driver genes included *NOTCH1* (*N*=8 cancers), *ARID1A* (*N*=7), *CNTNAP5* (*N*=3), *PIK3CA* (*N*=3) and *SMARCA4* (*N*=3) (Supplementary Tables 2 and 3). Additional genes with well-established roles in tumorigenesis (for example, *CDKN2A*, *FBXW7* and *SMAD4*) also harboured SNVs and indels of previously proven pathogenic effect. Of interest, the MSI+ cancers tended to harbour pathogenic mutations in classical colorectal cancer driver genes such as *KRAS*, *ACVR2A*, *PTEN*, *BAX* and *CTNNB1.* We found two cancers with mutations (p.Arg298Cys and p.Arg259X) in *STAG2*, a gene not previously associated with EAC, but known to be a driver gene in other cancer types^39^; further exploration of TCGA EAC exome data found 6 of 88 tumours with *STAG2* mutations (https://tcga-data.nci.nih.gov/tcga/). Intogen^40^ and MutSigCV^41^ analysis did not identify any further EAC driver genes at false discovery rate <0.05 and no pathogenic mutations were found in some of the previously suggested EAC drivers^38^,^42^, including *TRIM58*, *PNLIPRP3*, *UNC13B*, *TLR4* and *CCDC102B* (ref. 39).

Excluding a small number of mutations in regions of poor coverage in the Ampliseq panel, the high-depth sequencing on the same FF samples validated 66/70 (94%) of the driver SNVs and small indels. In addition, 10 mutations in EAC driver genes were called only in the Ampliseq data, including probably pathogenic changes in p53, *SMAD4*, *CDKN2A*, *PIK3CA* and *NOTCH1* (Supplementary Tables 3 and 4). The reasons for these changes not being present in the exome data were various, including low depth of coverage, low regional sequencing quality, and VAF below the calling threshold. In all cases where coverage and quality were acceptable, the mutation was retrospectively found to be present in the exome sequencing data. No driver was universally predicted to be clonal or sub-clonal, p53 and *SMARCA4* tending to be in the former category and *NOTCH1* in the latter.

**Figure 2:**
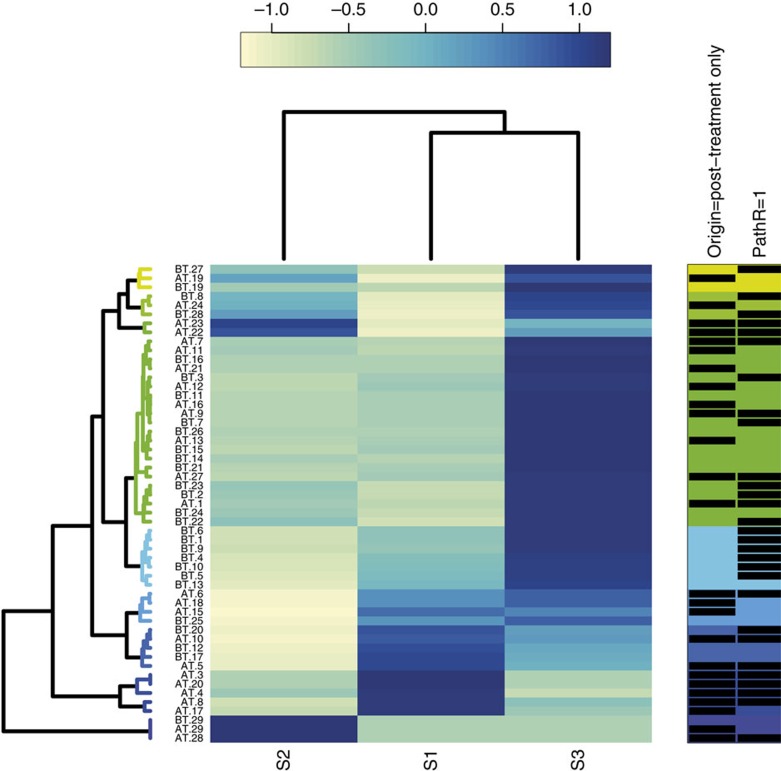
Mutation signatures. Cluster dendrogram showing the contribution of each composite mutation signature (S1-3) to the overall mutation spectrum of each pre- and post-treatment sample. The increasing contribution is reflected in the shading from light yellow through to dark blue. The predominant signature is taken as the darkest blue shading for each sample. Some samples are not shown if their mutation burden was too low for accurate signature assessment. The contribution of each SNV type to the three mutational signatures is shown in Supplementary Fig. 5.

SNV mutation spectra and signatures for all samples are shown in Fig. 2 and Supplementary Fig. 5. Almost all pre-treatment samples showed a predominant signature S3. This partially resembled the aging-associated Signature 1 of Alexandrov *et al.*^43^, with a predominance of C>T changes at CpG dinucleotides, but also included .TT>.GT changes, as previously noted in treatment-naive EACs^37^,^44^. In agreement with these data, patient age was positively correlated with the proportion of C>T changes in the pre-treatment samples (*P*=0.038). The .TT>.GT mutations were not related to age (*P*=0.762), perhaps because they originated from inflammation-induced oxidative damage in precursor lesions such as Barrett's oesophagus^38^. Of particular note, we found two further signatures (S1, S2) in some of our cancers: S1 resembled the Alexandrov smoking-associated signature #4 typified by C>A changes, whereas S2 did not clearly map to an Alexandrov signature, being characterized by both T>C (especially .TT>.CT) and C>A changes. S1 was the predominant signature in two cancers (#17 and #20) and S2 was present only in case #29, suggesting that these cancers arose owing to an aetiology that was not related to ageing and may have been acting throughout tumorigenesis. Patient #29, in particular, was exceptionally young (aged 29 years at presentation). His cancer had an unusual histology (Supplementary Fig. 2) and a relatively low mutation burden, and he was found to carry a p53 mutation in his morphologically normal oesophagus. DNA from other cell types was not available and no family history of cancer had been reported, suggesting that this patient may have been somatic mosaic for mutant p53.

In most cancers (*N*=23), we were able to assess somatic copy number alterations (SCNAs) and loss of heterozygosity (LOH) using SNP arrays. EACs were generally hypertriploid, and as others have noted previously^38^,^45^,^46^, chromosome arm-scale SCNAs were common (Supplementary Fig. 6), especially gains of chromosome 5p, 8q and 13q, and deletions of 4q, 9p, 15q, 17p, 19 and 22q. A median 18 large SCNAs, defined for convenience as changes involving more than half of a chromosome arm, were found per cancer (Supplementary Fig. 6). Focal SCNAs included amplification of known oncogenes (including *ERBB2*, *TERT*, *MYC* and *CCNE1*) and loss of tumour suppressors (including *ARID1A* and *CDKN2A*) (Supplementary Table 5). The two MSI+ cancers showed very few SCNAs and were hyperdiploid.

Several of the focal SCNAs represented actionable targets for therapy (Supplementary Table 6). In addition, a number of SNVs was potentially actionable, although some of these mutations were of uncertain pathogenic significance. The actionable changes were present variably in pre- and/or post-treatment samples, and hence not only represented potential targets for second-line therapy, but also suggested the potential utility of adding targeted therapy to first-line genotoxic treatment.

We searched for clinically useful molecular predictors of response to chemotherapy in the pre-treatment tumour. Response to chemotherapy was not associated (at *P*<0.05) with any specific mutated gene or SCNA, or with the burden of these changes, although there was evidence that a higher burden of pre-treatment SNVs and indels was associated with a good response (Supplementary Table 7). Neither MSI+ cancer responded well, in accord with the reported poor response of MSI+ colorectal cancers to chemotherapy^47^. Based on findings linking intra-tumour variability to outcome in other cancers^18^,^34^,^48^, we also used the VAF coefficient of variation in pre-treatment samples as a simple measure of genetic diversity, but this was not associated with response (*P*=0.178, Wilcoxon test). The proportion of T:A>A:T mutations in the pre-treatment sample was, however, associated with response independent of other clinico-pathological factors (median 7.1% T>A in responders versus 4.8% in non-responders, *P*=0.0028, Wilcoxon test; odds ratio using median=16, Supplementary Table 7).

All patients had a minimum follow-up of 5 years. In multivariate models, a good pathological response and absence of lymph node metastases were associated with better recurrence-free survival in a Cox proportional hazards model, as expected^2^, but no pre-treatment molecular or clinical variable predicted survival (Supplementary Table 7).

### Driver mutation changes following chemotherapy

We initially observed from the exome data that some cancers, especially clinical responders, showed large decreases in mutation burden after treatment (Supplementary Fig. 7; Supplementary Table 3). Since neo-adjuvant therapy can result in lesions with low tumour cell content, we assessed this possibility carefully (Supplementary Note 1). As a result, we excluded six post-treatment samples (four responders, two non-responders) on the basis of three features (Fig. 1; Supplementary Note 1): estimated large decreases in purity between the pre- and post-treatment samples; likely low tumour cell content (<20% in histological sections); and low mutation burden after therapy. After this process, several of the 24 remaining cancers still showed complete loss of mutations, including driver changes such as p53, *SMARCA4* and *ARID1A*, and complete gain of mutations was also observed, although less frequently (Fig. 1; Supplementary Table 3; Supplementary Fig. 7). We then assessed other explanations for these observations, principally: (i) copy number losses that deleted the mutation; (ii) intra-tumour heterogeneity/polyclonality, causing apparent mutation loss through tumour sampling; and (iii) clonal evolution as a result of therapy. We used p53 as an exemplar for the behaviour of EAC driver mutations after chemotherapy, owing to its relatively high mutation frequency and its established role in tumorigenesis from the stage of severe dysplasia onwards.

First, where assessment using SNP arrays could be performed, we found evidence that deletions in the post-treatment sample could contribute to decreased mutation VAF. For example, reduced chromosome 17p (p53) copy number occurred after therapy in cancers #5, #8 and #22, all of which showed loss of a p53 missense or nonsense mutation. Conversely, p53 copy number increase occurred after treatment in #27, which showed gain of a p53 missense mutation.

**Figure 3:**
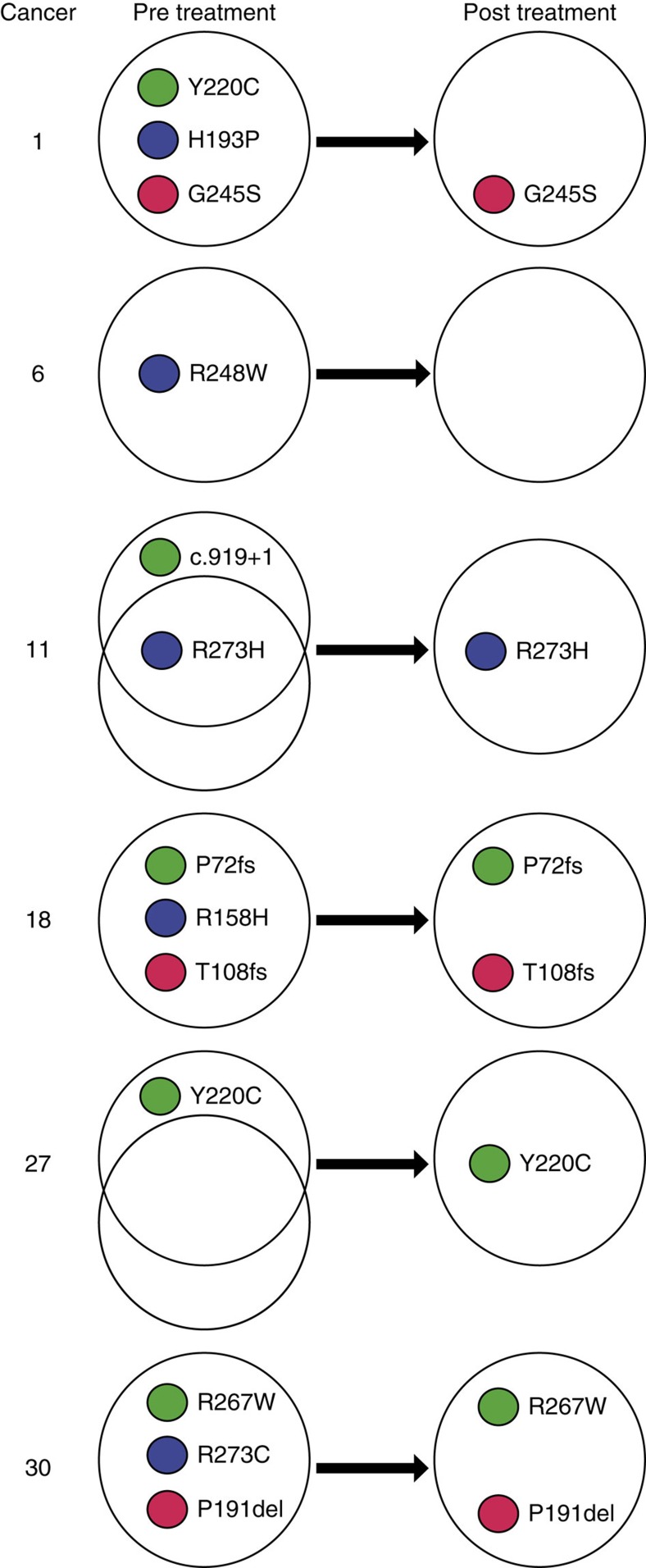
p53 mutation changes after treatment in selected cancers. The figure shows the cancers in which the p53 mutation complement (presence or absence of an SNV) changed between the pre- and post-treatment sample, based on the combined sequencing data. p53 VAF changes are shown in Supplementary Table 3, Supplementary Fig. 7 and Supplementary Note 1.

Second, several cancers showed evidence of intra-tumour heterogeneity/polyclonality, as reflected by the presence of multiple p53 mutations that behaved discordantly after therapy (Fig. 3; Supplementary Table 3). The MSI+ cancers were in this group, perhaps as expected, but other cancers (#1, #11) showed similar features.

**Figure 4:**
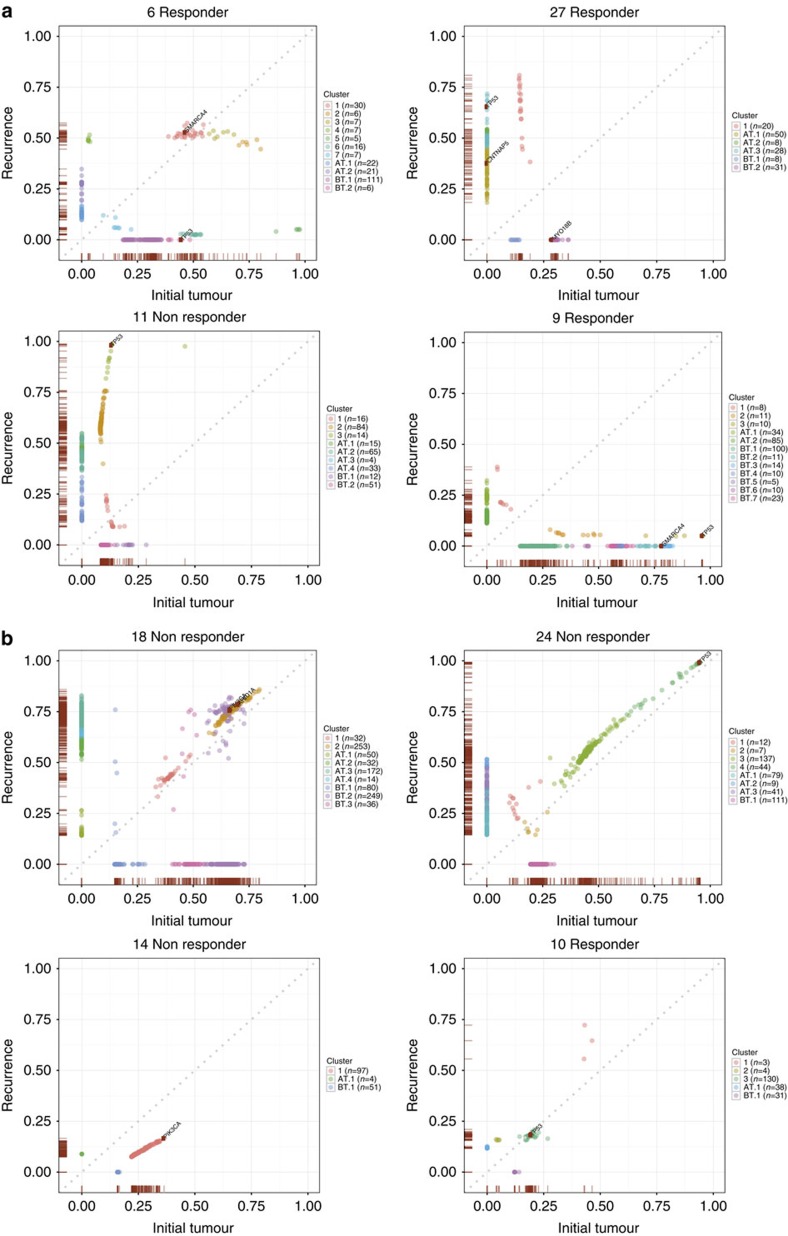
Exemplars of clonal evolution in cancers with and without evidence of genetic bottlenecking after treatment. The plots show posterior allele frequencies in pre-treatment (*x* axis) and post-treatment (*y* axis) paired samples in cancers (**a**) with and (**b**) without evidence of genetic bottlenecking after treatment. VAFs are corrected for copy number, this effect being greater at relatively high VAFs, but not for tumour purity. Each predicted clone or sub-clone is colour coded. The number of SNVs and small indels in each clone and the presence of the clone in pre-treatment (BT), post treatment (AT) or both samples are shown in the legend to the right of each plot. In order to provide high-confidence VAF estimates, a minimum of 60 reads was required for the inclusion of variants. For clarity of display, mutations with VAF<0.03 in both of the paired samples are not shown.

Third, in a single case (#6), loss of p53 mutation could be ascribed with confidence to clonal evolution after therapy (Fig. 4). This cancer showed no post-treatment copy number change at p53, evidence of problematic purity, or polyclonality for p53. Similarly, *SMAD4* mutation was lost in cancer #8, and actually showed copy number gain, strongly suggesting loss of its sub-clone.

### Clonal evolution of the EAC genome after chemotherapy

We then assessed in more detail the evolution of the 24 EACs as a result of chemotherapy and found a variety of clonal behaviours (Fig. 4; Supplementary Table 1; Supplementary Fig. 8). Eleven cases (9 non-responders, 2 responders) showed little change in clonal composition after therapy (Figs 1 and 3). In these lesions, although mutations at low VAF in the pre-treatment lesion were often lost from the post-treatment sample and a number of mutations were unique to the latter, we reasoned that these changes could be accounted for by sampling effects (Supplementary Fig. 9). The two responders in this category (#10, #23) both showed regression of the primary tumour by PET scan, as well as histology.

A different pattern of evolution was seen in the two MSI+ cancers, both non-responders. Mutations, including drivers, were lost from the pre-treatment cancer across the VAF spectrum. In fact, both MSI+ cancers contained three detectable, pathogenic p53 mutations (p.Arg158His/p.Thr102fs/p.Pro72fs in #18 and p.Arg267Trp/p.Pro191delCTC/p.Arg273Cys in #30) and the sequencing of additional FFPE tumour regions showed that some of these changes were spatially heterogeneous (Fig. 3). Each cancer showed loss or severe VAF reduction of one of these mutations in the post-treatment sample, while the other two mutations increased in VAF. The post-treatment samples had very large numbers of unique mutations across the VAF spectrum and these included potential driver changes in *PIK3R1* and *KMT2D.* The genomic evolution of the MSI+ cancers strongly suggested that these were polyclonal tumours. Another cancer (#1) was MSI- and a responder, but also harboured three p53 mutations (p.Gly245Ser/p.Tyr220Cys/p.His193Pro), plus LOH, and these mutations showed similar temporal and spatial behaviour to those in the MSI+ cancers. We therefore included this cancer in the polyclonal group although its level of clonal complexity appeared lower than that of the MSI+ cases.

A further 10 cancers (Fig. 1) showed evidence of having passed through a genetic bottleneck of varying severity based on the following features (Fig. 4; Supplementary Fig. 8): loss of many mutations, including some not at low VAFs, from the pre-treatment sample; sharing of a non-trivial number of mutations between pre- and post-treatment samples; and increased frequency in the post-treatment samples of a clone that was uncommon in the pre-treatment sample. Most, but not all, cancers that had passed through a bottleneck were responders (Fig. 1). In the two non-responders with evidence of a bottleneck (#11, #12), there was evidence of some radiological reduction in tumour volume after the course of chemotherapy. Some of the ‘bottlenecker' cancers showing the clearest behaviour were as follows. Cancer #6 was noteworthy for having lost its p53 mutation after treatment owing to loss of the clone containing this mutation. The post-treatment cancer retained its pathogenic *CDKN2A* and *SMARCA4* mutations and gained a low VAF, truncating *ARID1A* change. In cancer #8, p53, *SMAD4*, *CNTNAP5* and 1,006 other mutations were lost entirely after treatment. Some of these changes, such as p53 and *CNTNAP5* loss, could potentially be explained by SCNAs, although most could not (details not shown). The post-treatment sample of #8 harboured 170 mutations with VAF>0.03 (112 of which were not found pre-treatment), 85 with VAF>0.1 and 25 with VAF>0.2. In the absence of sequencing quality issues (for which there was no evidence) or major non-neoplastic clone(s) present in this sample (which is an intriguing possibility that remains hypothetical), the loss of *SMAD4*—which is the only EAC driver reported as mutated in carcinomas but not in precursors such as Barrett's oesophagus^38^—raises the possibility that the remaining post-treatment cancer in patient #8 represents an earlier stage of EAC development, such as an early cancer or even severe dysplasia. Cancer #11 showed loss of the major pre-treatment clone after treatment, and expansion of a sub-population of cells from the minor clone. It also showed loss of one p53 mutation (c.919+1G>T splice site) and expansion of another (p.Arg273His) after therapy. Two *NOTCH1* mutations (p.Pro1381fs, p.Ala465Thr) were lost and two other driver mutations (*FBXW7* p.Arg465Cys, *SEMA5A* p.Glu832X) were within a sub-clone that expanded greatly after treatment. Cancer #12 showed loss of the major pre-treatment clone and expansion of a minor clone including p53 and *SMARCA4* mutations. Cancer #27 lost the major pre-treatment clone that contained a *MYO18B* mutation after therapy, but gained p53 and *CNTNAP5* mutations not present before therapy, alongside expansion of a minor pre-treatment clone. We subsequently found the p53 mutation to be present in the additional FFPE tumour region analysed and hence, spatially heterogeneous (sub-clonal) in the primary cancer. The other cancers with probable bottlenecks variably showed loss, gain and retention of driver mutations.

**Figure 5:**
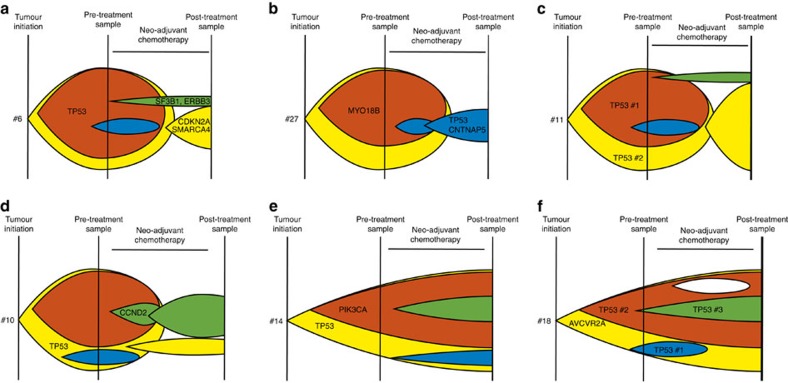
Schematic of putative evolution and sampling of patients with respect to genetic bottlenecks and clinical response. Although the effects of sample time and place, tumour growth and shrinkage, and clone-specific growth and shrinkage mean that the observed data could be explained by a number of behaviours, these diagrams show some plausible scenarios of cancer sub-clone frequencies (coloured) in relation to treatment and sampling times: (**a**) bottleneck in which a few mutations post treatment are ‘new' (green), many are lost (red) and yet others have unchanged frequency (for example, #6); (**b**) bottleneck in which many mutations are entirely new (blue) (for example, #27); (**c**) bottleneck similar to **a** in which there is no clinical response (for example, #11); (**d**) no bottleneck but with clinical response (for example, #10); (**e**) no bottleneck or clinical response (for example, #14); and (**f**) complex, possibly polyclonal evolution without bottleneck (for example, #18). Mutations from these six cancers are mapped onto their putative positions on the evolutionary diagram.

Putative clonal histories with respect to bottlenecking and response are shown in schematic fashion in Fig. 5. Overall, we found that responders tended to show bottlenecks and non-responders, no major clonal shifts (Fig. 1), with the distribution of responder and non-responder cancers between the three clonal behaviours being formally non-random (*P*=0.013, Fisher's exact test). We surmised that the four cancers discordant for response and bottlenecking could be explained as follows: in bottleneckers with Mandard grade 4 or 5 cancer at resection (#11, #12), there was rapid tumour re-growth after an initially good response; and in non-bottleneckers with a Mandard grade ≤3 tumour (#10, #23), clonally uniform loss of cancer cells related to factors other than their genotype may have occurred.

### Driver mutations in post-chemotherapy samples

We searched for mutations that could confer chemotherapy resistance and/or drive the growth of cancers that had passed through a bottleneck, specifically SNVs and focal SCNAs found only in the post-treatment sample. Twenty-four SNVs were found in known cancer driver genes (Table 2), some of which were EAC drivers (p53, *FBXW7*, *SMARC4A*, *CNTNAP5*) and/or pan-cancer drivers (for example, *SF3B1* and *TAF1*). Ten of the 24 mutations were scored as probably functional. Four post-treatment cancers also showed focal copy number gains of *ERBB2*, *CCND2*, *TERT* and *CCNE1* that were absent from the pre-treatment samples (Table 3). A further cancer (#22) acquired a homozygous deletion of the gastrointestinal cancer predisposition gene *MUTYH* and, in accordance with defective base excision repair, showed an ∼3-fold increase in the proportion of G:C>T:A SNVs in the post-treatment sample (Supplementary Fig. 10).

### Other changes in the EAC genome after therapy

We examined the spectrum of SNVs, comparing mutations that had definitely occurred before therapy (either specific to the pre-treatment sample or shared) with those that were present only in the post-treatment samples. *En masse*, there were highly significant differences between the mutation spectra before and after chemotherapy (*P*<0.0001, *χ*^2^ test, Supplementary Fig. 10). Most notably, there was a decrease in C>T frequency and an increase in C>A frequency after treatment, as also found in a study of eight samples by Muruguase *et al.*^34^. These changes were reflected in significant (*P*<0.01, Fisher's exact test) overall mutation spectrum changes between individual pre- and post-treatment pairs in 11/23 patients (Supplementary Fig. 10), in some cases owing to large shifts in the frequencies of mutations other than C>T and C>A. Several cancers showed a change in mutation signature from S3 to S1 or S2 following treatment (Fig. 2; Supplementary Fig. 5). Good responders were more likely to show a signature change (*P*=0.05, Fisher's exact test), suggesting that the signature resulted from the effects of chemotherapy, either directly (for example, from nucleotide excision repair of platinum adducts), or from the selection of a resistant sub-clone that had few ageing-associated mutations, but had acquired mutations on a specific environmental background.

Excluding the MSI+ cancers that had very low SCNA burdens, the median proportion of large SCNAs shared between the 21 pre- and post-treatment pairs analysed was 38% (range 6–95%; Supplementary Fig. 6). In keeping with the SNV clonal shift data, fewer mutations were shared by the pre- and post-treatment samples of responders than of non-responders (medians 32% versus 47% shared, *P*=0.033, Wilcoxon test). A similar association was seen with bottlenecking, although this was formally non-significant, plausibly owing to the excluded samples (medians 34% versus 46%, *P*=0.100). Gain of chromosome 4q and loss of 1q and 20 were specifically more frequent in post-treatment than pre-treatment cancers, especially those of responders (Supplementary Fig. 6).

### Sequencing of additional tumour regions

For four cancers (#16, #18, #27 and #28), extra regions of both pre- and post-treatment cancer were available from FFPE tissue, and additional single pre- or post-treatment FFPE samples were available from a further nine cancers. These samples were sequenced using the Ampliseq Comprehensive Cancer Panel. Median average sequencing depth (Supplementary Fig. 11) was 1,228 × (range 463–2,373). A few samples harboured driver gene mutations (in *ARID1A* and *NOTCH1*) that had not been not found by exome sequencing or Ampliseq in the equivalent FF sample and conversely, some frozen sample driver mutations, notably also in *NOTCH1*, were absent from the FFPE samples (Supplementary Table 8). In the whole set of FFPE samples, excluding *NOTCH1* mutations that appear often to be polyclonal^34^, 55/66 (83.3%) mutations were concordantly present or absent in the FF and FFPE samples. A small number (*N*=7) of mutations apparently gained in the post-treatment FF sample were actually present in the FFPE pre-treatment sample, indicating spatial sub-clonal heterogeneity in the latter and artefactual gain owing to sampling effects^34^. Genetic divergence between spatially distinct pre-treatment biopsies was not predictive of response to therapy (details not shown).

## Discussion

The clinicopathological response of oesophageal adenocarcinoma to neo-adjuvant chemotherapy predicts survival after surgery^1^, whether as a direct consequence of residual tumour burden, or as a marker of how effectively metastases have been treated. In this study, we have shown that the response of the EAC genome to neo-adjuvant oxaliplatin and 5-fluorouracil varies greatly, with cancers falling into three main groups. First, some EACs appear intrinsically resistant to therapy and show only minor genomic changes after treatment, plausibly resulting from tumour sampling effects alone. Second, a small number of cancers, especially MSI+ lesions, tend to have multiple driver gene mutations that variably increase or decrease in frequency after treatment, sometimes showing complete loss or gain. However, the overall genetic complexity of the cancer is largely unchanged by therapy. We propose that these tumours have a high degree of polyclonality and spatial heterogeneity, such that the observed changes after treatment largely reflect sampling effects, although differential effects of therapy on sub-clones remain possible. In the final group of cancers, profound clonal shifts strongly reminiscent of genetic bottlenecking occur. A good clinical response is associated with a bottleneck, but importantly, some poor responders go through bottlenecks and some good responders do not.

We used p53 as an exemplar for the behaviour of EAC driver mutations after chemotherapy. While we took considerable care to address the influence of treatment-induced changes in tumour purity on our major findings and excluded six cancers on that basis, we accept that some instances of p53 loss in the remaining 24 cancers might still be artefacts of low purity in post-treatment samples. In other cases, p53 loss might simply result from a SCNA deleting the mutant allele, leaving a tumour that remains p53-deficient, although the replacement of a missense mutation with a null mutation might still have functional consequences^49^. Irrespective of potential purity problems, however, we believe that the three types of clonal evolution described above remain valid. While some cancers retain their p53 mutation after treatment, others harbour multiple p53 SNVs and/or SCNAs that can be lost, gained or change in frequency after treatment, even if the post-treatment cancer remains p53-mutant. These data strongly suggest that p53 mutations can be polyclonal in EACs. Finally, as seen most clearly in cancer #6, p53 mutations can be lost in the absence of a copy number change as it resides within an EAC sub-clone that is lost as the cancer passes through a genetic bottleneck. We speculate that the few complete pathological responders in the clinical trial (who could not, of course, be analysed) may have shown even more severe bottlenecking than that found in our analysable, good responder cancers.

The nature of the remaining tumour in good responders is a little uncertain, as EAC morphology after chemotherapy can be hard to assess. It has been speculated that precursor lesions such as Barrett's oesophagus are more resistant to chemotherapy than EAC proper. We wonder whether early EAC or severely dysplastic Barrett's oesophagus was present at non-trivial levels in some of our post-treatment specimens, for example reflecting the loss of the *SMAD4* mutation after treatment in cancer #8. Since Barrett's oesophagus may be polyclonal and variably harbours p53 and other driver mutations, this scenario might also explain the loss of p53 mutation in cancer #6. Further work is, however, required to confirm or refute this possibility.

Studies of haematological malignancies have shown that genetic bottlenecks can occur in patients who relapse after an initially good response. In a few cases, they have also demonstrated large changes in known driver genes. For example, Griffith *et al.*^50^ showed loss of *IDH1* mutation, accompanied by an *IDH2* mutation rising from 2% frequency to clonal driver status in an acute myelogenous leukaemia (AML) patient, similar to our findings for p53 in cancer #11. Fewer studies have examined the effects of chemotherapy on the carcinoma genome, especially in the neo-adjuvant setting in which response, rather than recurrence, is the primary endpoint. Our results are, however, consistent with previous reports. Using targeted Sanger sequencing and Pyrosequencing, Jiang *et al.*^28^ reported that the allele frequencies of p53 and *PIK3CA* mutations tended to fall in breast cancers after they responded to taxol and carboplatin, but the study did not perform deep sequencing or examine tumour purity changes. Other groups have reported that recurrent brain tumours can show bottleneck behaviour affecting the major clone after genotoxic primary or adjuvant treatment with temozolomide^32^,^33^. Of note, the brain tumour studies found no good evidence of specific, recurrent chemotherapy resistance mutations. Possible explanations for this include polygenic mechanisms of resistance, multiple resistant sub-clones within cancers, chemoresistance of non-genetic origins and large-scale genetic heterogeneity resulting in many potential resistance genes, and hence sub-optimal power for their detection. In general, our data were in accordance with the brain tumour findings, although we did find a few cancer-associated mutations that could have conferred therapeutic resistance and/or acted as post-chemotherapy drivers.

As regards EAC sequencing, a recent study by Murugaesu *et al.*^34^ examined neo-adjuvant chemotherapy in eight EAC patients, with a focus on multi-region sequencing. Our study is concordant with Murugaesu *et al.* in some respects, including the importance of SCNAs, sub-clonal *NOTCH1* mutations, the widespread genome doubling, and the change in mutation spectrum after chemotherapy. However, Murugaesu *et al.* did not perform very deep sequencing or array-based SCNA assessment, and examined only one responder who had post-treatment samples available. They were consequently unable systematically to examine genetic bottlenecks or relate the genomic changes they found to patient response. Murugaesu *et al.* reported that EACs have stable genetic vulnerabilities, unimpeded by cytotoxics and suitable for therapeutic intervention, but our data show that this is not generally true, although it holds for a sub-set of patients, largely poor responders.

It is interesting that platinum therapies are postulated to require deficient homologous recombination for their effects. Several studies (summarized in ref. 51), have suggested p53 mutations to be a marker of poor response and outcome in EAC. p53 mutation has also been suggested as causal for platinum resistance owing to the failure of pro-apoptotic pathways. Typically, however, associations between tumour markers and pathological response to chemotherapy are assessed using surgical resection specimens. In our study, p53 mutations were indeed over-represented in the post-treatment samples of non-responders (*P*=0.046, Fisher's exact test). However, it was not the p53 SNV in the primary cancer, but persistence of p53 mutation in the post-treatment tumour that was associated with a poor outcome. It follows that biomarkers derived from post-therapy samples should be used with caution in the clinical setting.

Our study has some clinical implications. For example, molecular (bottleneck) and clinical (enhanced PET) response are not always in accordance. This is of particular clinical importance, as presently a number of centres and studies use interval metabolic response on PET after one cycle of chemotherapy as a surrogate of response, and a basis for aborting or continuing neo-adjuvant therapy^52^. Similar considerations apply to Mandard grade, which remains the mainstay of assessing response after surgery, but is prone to inter-observer variability. Not only can a clinical response occur without a bottleneck, but more importantly a bottleneck can occur in the absence of an apparent clinical response. In the latter case, it is quite possible that the cancer has responded well initially and re-grown equally rapidly, either subsequent to shrinkage of the major clone or in parallel with it. This begs the question of whether additional therapy, in parallel or contiguously, could prevent emergence of the resistant clone in bottlenecker non-responders.

In summary, even a short course of pre-operative chemotherapy can cause rapid EAC genome evolution. Similarly rapid changes have been shown when tumours acquire resistance to targeted therapies^24^. A good clinical response and/or genetic bottlenecking is predicted by a large SNV burden and high proportion of T:A>A:T mutations in the pre-treatment EAC although not by the level of genetic diversity. The clinical utility of these biomarkers requires assessment in independent studies. A good response is usually accompanied by a variably tight genetic bottleneck, although bottlenecks also occur in some non-responders. The remaining cells in such tumours may harbour driver mutations not detectably present in the primary cancer. By contrast, many other EACs, especially non-responders, show relatively few new genetic changes after therapy. Overall, our data suggest that anti-EAC therapy should not be based solely on pre-treatment biopsies. Instead, a combination of improved imaging^52^ and molecular monitoring during treatment—for example, by repeated biopsies or using circulating tumour DNA—has the potential to classify response better and to suggest timely additional treatment strategies based on cytotoxics or therapies targeted to mutations in the residual cancer.

## Methods

### Patients and samples

Patients with oesophageal adenocarcinoma were drawn from a phase II of neo-adjuvant oxaliplatin and 5-fluorouracil for oesophageal cancer (EudraCT number 2005-000834-34). All patients provided written informed consent for treatment and molecular analysis, with ethical approval provided by the Oxfordshire Regional Ethics Committee. Recruitment took place between May 2006 and February 2010. Inclusion criteria were: age over 18 years; histological evidence of locally advanced (>T1N0; UICC-AJCC 6th edition 37) invasive cancer of the oesophagus or gastro-oesophageal junction (excluding Siewert type III tumours); no evidence of unresectable disease; World Health Organisation (WHO) performance status 0–2; absence of any clinically significant disease likely to interfere with study evaluations; and adequate haematological, renal and hepatic function. Exclusion criteria included: pregnant or breast-feeding women; and dihydropyrimidine dehydrogenase deficiency. Patients were staged sequentially by contrast-enhanced CT, endoscopic ultrasound (EUS), ^18^F-FDG PET-CT, and laparoscopy (for tumours extending below the diaphragm). Patients received two cycles of oxaliplatin-5FU: each lasted 21 days, and comprised 130 mg m^−2^ intravenous oxaliplatin on day 1, followed by 1,000 mg m^−2^ per day intravenous 5-fluorouracil on days 1–4. Following completion of therapy, restaging was performed by PET-CT. In the absence of progression to unresectable disease, patients underwent attempted surgical resection 4–6 weeks later.

Forty of 51 patients in the trial had adenocarcinoma. Random biopsies comprising 2–3 mm^3^ of the primary cancer and normal squamous oesophageal epithelium 5–10 cm proximally were taken at endoscopic ultrasound examination within 3 weeks of diagnosis and before instigation of therapy. Some of these samples were snap-frozen; others were fixed in formalin and paraffin embedded. Serial haematoxylin-and-eosin examination confirmed the presence of an estimated >60% malignant cells in each cancer sample. Thirty-six patients proceeded to surgery 12–14 weeks after diagnosis (one patient died during chemotherapy, one was unable to undergo resection due to performance status, and two had metastatic disease evident on re-staging scan). In 2 of the 36, unresectable disease was encountered; successful resection was therefore performed in 34 patients. Of these, the resection specimen underwent histopathological assessment within 1 h of resection, and 3–4 mm^3^ macrodissected samples were taken randomly from areas of cancer and frozen for molecular analysis; haematoxylin-and-eosin examination confirmed the presence of an estimated >70% malignant cells in each sample. One tumour underwent complete histopathological regression, leaving 33 tumours potentially suitable for analysis, of which 30 were chosen. Normal tissue >5 cm proximal to the primary tumour was again taken and frozen for analysis.

Mandard grading was performed before molecular work being performed according to the standard criteria by two independent, blinded observers (J.M.F. and L.M.W.) with any discordance resolved by consensus.

Genomic DNA was extracted from tumour and normal samples after homogenization using the Qiagen Dneasy Blood and tissue kit according to the manufacturer's instructions. Total messenger RNA was also extracted using the Qiagen AllPrep DNA/RNA Micro Kit (Qiagen Inc., Valencia, CA) in accordance with the manufacturer's instructions.

### Exome sequencing

Exome capture was performed using the Illumina TruSeq or Nextera method. Samples were quantified using the Qubit system (Invitrogen) and sequencing libraries constructed from 1 μg DNA post capture using the NEBNext DNA Sample Prep Master Mix Set 1 Kit (NEB). Ligation of adaptors was performed using 6 μl of the Illumina Multiplexing Sample Preparation Oliogonucleotide Kit. Libraries were size-selected using 2% gel electrophoresis and the distribution of fragments in the purified fraction was determined using the Tapestation 1DK system (Agilent/Lab901). Each library was PCR-enriched using the following custom primers:

Multiplex PCR primer 1.0:

5′-AATGATACGGCGACCACCGAGATCTACACTCTTTCCCTACACGACGCTCTTCCGATCT-3′

Index primer:

5′-CAAGCAGAAGACGGCATACGAGAT[INDEX]CAGTGACTGGAGTTCAGACGTGTGCTCTTCCGATCT-3′

Indexes were 8 bp long and part of a system developed in-house. Four independent PCR reactions per sample were prepared using 25% volume of the pre-PCR library each. After eight cycles of PCR (cycling conditions as per Illumina recommendations), the four reactions were pooled and purified with AmpureXp beads. The final size distribution was determined using the Tapestation 1DK system (Agilent/Lab901). The concentration of each library was determined by the Agilent qPCR Library Quantification kit. Samples were sequenced using the Illumina HiSeq2000 platform as paired 100 bp reads with Chemistry version 3.0, with the aim of a target average coverage of 100 × for the blood DNA and 200 × for the tumours.

Reads were mapped with Stampy version 1.0.21 (ref. 53) and BWA version 0.7.0 (ref. 54) onto the decoy Human Reference Genome (GRCh37d5). Duplicated reads were removed with Picard suite (version 1.111) (http://broadinstitute.github.io/picard/) and realigned around indels with the Genome Analysis Toolkit version 3.1-1 (ref. 55). SNVs and small insertion–deletions (indels) were called with Platypus version 0.5.2 (ref. 56). Normal, pre-chemotherapy tumour (BT) and post-chemotherapy tumour (AT) samples were analysed together to ensure comparable calls at every locus. Reads with mapping quality <30 and base quality <20 were discarded. Variants were only kept if they passed the standard Platypus filtering criteria, with the exception of variants showing allele bias to increase sensitivity for mutations in sub-clones or SCNAs. An additional set of SNVs was identified using MuTect version 1.1.4 (ref. 57) to increase our sensitivity for somatic mutations. Variants were annotated using SnpEff version 3.6b (ref. 58) and VCFtools version 0.1.12 (ref. 59).

Putative somatic mutations were defined as variant sites in any of the tumours that were different from the paired normal samples. Then, an empirically based filtering was applied to identify high reliable/quality somatic variants, specifically removing:

- less than 10 reads in total at the variant site in the normal sample and <8 reads in tumour samples;
- VAF ≥0.03 in normal;
- less than four variant alleles;
- VAF ≤0.02;
- presence of variant in public databases of germline variants (Exome Variant Server, 1000 genomes project, Complete Genomics 69 reference genomes) at a frequency ≥1%;
- variants identified in constitutional DNA from any of the other local, non-cancer sequencing projects (for example, 29 million variants across 284 samples from the Oxford-Illumina WGS500 consortium), owing to their likely origin as systematic errors in our pipeline; and
- presence in a segmental duplication region (>90% homology) or a region with mappability score <0.8.

Since we wished to compare pre- and post-treatment samples, we required only one member of each tumour pair to pass filters (iii) and (iv). Finally, for selected variants, and all variants in EAC driver genes (whether detected using exome sequencing or Ampliseq), we made sure the automatic call matched the data by expert visual inspection (I.T.) of the reads mapped onto the reference genome using read direction colouring on top of the standard integrated genomics viewer scheme (http://www.broadinstitute.org/igv/alignmentdata/). All variants in regions of poor quality were excluded.

### Ion proton sequencing

The Lifetech/ThermoFisher Ion AmpliSeq comprehensive cancer panel (http://www.lifetechnologies.com/order/catalog/product/4477685) was used for technical validation and deep sequencing of exome sequence data using the same FF DNA samples as used previously, and additionally for a small number of sample trios and some unpaired samples with additional formalin-fixed, paraffin-embedded biopsies from the same patients. A total of 40–60 ng of total genomic DNA was used to amplify targets according to the manufacturer's protocol (LifeTechnologies, cat. no. 4477685 and 4480442). Briefly, PCR cycling used for amplifying target DNA was an initial denaturation step at 99 °C for 2 min, followed by 16 cycles of 99 °C for 15 s and 60 °C for 8 min. The primer sequences within the PCR products were partially digested with FuPa reagent at 50 °C for 10 min, 55 °C for 10 min and 60 °C for 20 min, followed by ligation of IonXpress sequencing barcode adaptors (cat. no. 4471250). Adaptor-ligated DNA was purified using Agencourt AMPure XP reagent beads (Beckman Coulter, cat. no. A63881). Library amplification was performed with an initial denaturation at 98 °C for 2 min, followed by five cycles of 98 °C for 15 s and 60 °C for 1 min. Libraries were size-selected between 100 and 300 bp using Agencourt AMPure XP reagent beads and eluted in 50 μl lowTE (Life Technologies). Libraries were quantified using the Agilent Bioanalyzer high sensitivity DNA kit (Agilent, cat. no. 5067–4626).

A volume of 70 μl of diluted libraries at 200 pM were loaded via the Ion Chef instrument (Life Technologies, cat. no. 4484177) onto Ion PI chip v2 BC (cat. no. 4484270). Chips were sequenced on the Ion Proton sequencer (cat. no. 4476610) initialized with the Ion PI CI sequencing 200 kit (cat. no. 4488377) according to the manufacturer's instruction. Signal processing and base calling were performed using the Ion Proton Torrent suite (version 4.4). Variant calling was performed using variantCaller and Ion Reporter with a generic default parameter. The sites of variants present in the Ampliseq and/or exome sequence data were assessed alongside each other using both automatic calls in the Torrent Server output and visual inspection in the integrated genomics viewer.

The quality control filters were set as per the exome sequencing data, except that tumour minimum VAF was set at 0.02 for the FF samples (to reflect standard Ion Reporter somatic calling for high-depth sequencing) and 0.05 for the formalin-fixed, paraffin-embedded samples to reflect greater potential PCR bias and possible low-level deamination. Again, all variants passing VAF thresholds in one sample were visually inspected and reported in the other samples.

### Mutational signatures

Signatures of somatic mutations were inferred using the Bioconductor SomaticSignature package version 2.2.1 (doi: http://dx.doi.org/10.1101/010686). Briefly, the observed mutation spectrum is mathematically decomposed using a non-negative matrix factorization method^60^. The decomposition was performer for an a priori set of two to eight signatures. The optimal number of signatures (*r*=3) was manually chosen based on the maximum differentiation between the signatures. The conclusions of our analysis in terms of spectral changes after therapy remained if two or four signatures were assumed.

### Clonal structure analysis

Allelic heterogeneity, and thus clonal structure, was evaluated using PyClone algorithm (version 0.12.3)^61^. Allelic frequencies of selected somatic mutations were obtained using the number of reads and the number of reads carrying a variant. The copy number value at each of these loci was obtained from Onco-SNP (or VarScan2 where SNP array data were unavailable). For multi-sample analysis, only somatic mutations shared between pre-treatment and post-treatment samples, with at least 60 × coverage in one of the samples were included to provide a robust measure of clonal shifts based on precedent^33^. In addition, we included also shared positions with an allelic ratio <0.03 in just one of the samples. Mutations annotated as a candidate drivers were included in all the analyses regardless of their coverage. PyClone was run with a 10,000 iterations and a burn-in of 1,000 as suggested by the authors (http://compbio.bccrc.ca/software/pyclone/). To reduce the running time in the analysis of hypermutated cancers #18 and #30, PyClone was run for these samples with a randomly selected set of 500 variants.

### Mutation and gene set analysis

Gene-based and pathway analyses to detect significantly over-represented mutant genes and pathways were performed by IntOGen version 2.4.1 (ref. 40) and MutSigCV^41^ using the annotated, quality-filtered, somatic mutations. Mutation set analyses were performed using the CPDB (http://cpdb.molgen.mpg.de/).

### Copy number analysis

Where DNA was available, we analysed pre- and post-treatment tumour samples using Illumina Core Exome 24 arrays using standard methods. The OncoSNP v2.1 program (https://sites.google.com/site/oncosnp/user-guide) was used in unpaired mode. We used the default top ranked ploidy estimate. A copy number change was called if a region showed an absolute copy number that differed from the genome-wide mean by >1 in either direction. LOH was called using the default program settings. We presented data from two types of OncoSNP analysis: rank 1 (highest confidence) calls, which tend to be large regions of change; and all rank 1–5 calls, which include smaller regions with somewhat weaker evidence in favour of SCNAs and/or LOH.

Where DNA was unavailable for SNP array analysis, copy number was estimated using VarScan2 (version 2.3.7)^62^ using matched tumour-normal pairs using default parameters and adjusting for data and GC content. Sex chromosomes and low mappability regions extracted from the ENCODE ‘DAC blacklisted' table of the UCSC genome browser were excluded from the analysis. Winsorization using the Median Absolute Deviation was performed to detect and modify extreme logR values and genomic segments of constant logR were identified and merged at individual level by the bioconductor package copynumber (version 1.6.0)^63^ (*γ*=1,000). Gains and losses were called using a logR threshold of ±0.15. These data were only used for incorporation into the Pyclone program.

### Microsatellite instability

MSI was evaluated in the exome sequence data with MSIsensor version 0.2 (ref. 64) using default parameters and filtered using a 0.05 false discovery rate threshold.

## Additional information

**Accession code**: The genome sequence and array data have been deposited in the European Genome-phenome Archive under accession code: http://www.ebi.ac.uk/ena/data/view/PRJEB12657

**How to cite this article**: Findlay, J. M. *et al.* Differential Clonal Evolution in Oesophageal Cancers in Response to Neo-Adjuvant Chemotherapy. *Nat. Commun.* 7:11111 doi: 10.1038/ncomms11111 (2016).

## Supplementary Material

Supplementary Information Supplementary Figures 1-11, Supplementary Tables 1-8, Supplementary Note and Supplementary References.

## Figures and Tables

**Table 1:** Clinical features of each patient.

**Case no.**	**Age**	**Sex**	***T***	***N***	**PathR**	**Mandard**
1	69	Male	2	0	Yes	3
2	78	Male	3	1	Yes	3
3	49	Male	3	1	Yes	3
4	53	Male	2	1	Yes	3
5	73	Male	3	0	Yes	2
6	63	Male	2	0	Yes	2
7	68	Male	3	1	Yes	3
8	54	Male	3	0	Yes	2
9	58	Female	2	0	Yes	3
10	61	Male	1	1	Yes	3
11	71	Male	3	1	No	4
12	56	Female	3	1	No	5
13	67	Male	2	1	No	4
14	62	Male	3	1	No	4
15	67	Male	2	1	No	4
16	60	Male	3	1	No	4
17	77	Male	3	1	No	5
18	68	Female	2	0	No	5
19	65	Male	2	3	No	4
20	65	Male	3	1	Yes	3
21	70	Male	3	1	No	4
22	60	Male	3	0	Yes	3
23	73	Male	3	0	Yes	3
24	57	Male	1	1	No	5
25	65	Female	3	1	No	4
26	76	Male	2	0	No	4
27	78	Female	4	0	Yes	3
28	74	Male	2	0	Yes	3
29	29	Male	3	0	No	5
30	69	Female	3	1	No	4

T, N AJCC tumour and node stage; PathR, binary pathological response derived from Mandard score, Mandard, Mandard response grade. Further details are provided in Supplementary Table 1.

**Table 2:** Cancer driver mutations specific to post-chemotherapy tumours of responders and/or bottleneckers.

**Case**	**Chr**	**Position**	**Gene**	**Mutation**	**Functional?**
5	X	123,220,581	STAG2	p.Gly1080Arg/c.3238G>A	Possible
6	2	198,267,720	SF3B1	p.Tyr587His/c.1759T>C	Probable
6	12	56,482,338	ERBB3	p.Val296Met/c.886G>A	Probable
7	3	126,722,305	PLXNA1	p.Glu504Lys/c.1510G>A	Possible
7	4	144,446,615	SMARCA5	p.Gly178Ser/c.532G>A	Probable
7	4	187,628,194	FAT1	p.Pro930Ala/c.2788C>G	Unlikely
7	14	58,817,866	ARID4A	p.Lys494_Asp495ins2/c.1482_1483ins6bp	Probable
7	15	57,545,555	TCF12	p.Ile456fs/c.1366_1367ins13	Yes
7	21	35,154,412	ITSN1	p.Ile600Thr/c.1799T>C	Probable
8	10	32,561,062	EPC1	p.Ala633Ser/c.1897G>T	Possible
8	12	124,812,146	NCOR2	p.Glu2338fs/c.7012delG	Probable
9	7	140,494,212	BRAF	p.Phe346Leu/c.1036T>C	Unlikely
11	2	198,269,813	SF3B1	p.Pro509Gln/c.1526C>A	Probable
11	3	168,845,652	MECOM	p.Gln270His/c.810A>C	Probable
11	9	16,419,233	BNC2	p.Glu1018Asp/c.3054A>T	Unlikely
11	X	70,598,761	TAF1	p.Asp413His/c.1237G>C	Probable
12	20	31,022,659	ASXL1	p.Arg710Lys/c.2129G>A	Unlikely
22	12	49,437,523	KMT2D	p.Ala1788Thr/c.5362G>A	Possible
27	2	74,128,460	ACTG2	p.Ala8Thr/c.22G>A	Probable
27	2	125,555,816	CNTNAP5	p.Val1045Leu/c.3133G>T	Unlikely
27	6	129,766,853	LAMA2	p.Ala2106Pro/c.6316G>C	Possible
27	6	152,647,435	SYNE1	p.Gln5097X/c.15289C>T	Possible
27	17	7,578,190	TP53	p.Tyr220Cys/c.659A>G	Yes
28	11	3,707,397	NUP98	p.Glu1494Asp/c.4482G>T	Possible

Driver mutation status was obtained from the IntOGen database. The predicted functionality is shown as Yes (proven)/probable/possible/unlikely as assessed using functional prediction (SIFT, Polyphen2) and previous reports of mutations at that residue in COSMIC and the cBIO portal.

**Table 3:** Focal copy number driver mutations specific to post-chemotherapy tumours of responders and/or bottleneckers.

**Case**	**Chr**	**Start position**	**End position**	**Gene**	**Gain/deletion**
4	17	37,771,746	38,948,438	ERBB2	Gain
10	12	4,066,795	4,823,986	CCND2	Gain
22	1	44,844,958	46,743,900	MUTYH	Deletion
27	5	38,139	1,493,608	TERT	Gain
27	19	28,959,499	30,105,969	CCNE1	Gain

These changes were present only in the post-treatment samples of the 23 cancers analysed for SCNAs.
