# Supplementary material for: Differential clonal evolution in oesophageal cancers in response to neo-adjuvant chemotherapy
Source: Nat Commun. 2016 Apr 5;7:11111. doi: 10.1038/ncomms11111 (PMC4822033; doi:10.1038/ncomms11111)
Supplement: Supplementary Information — Supplementary Figures 1-11, Supplementary Tables 1-8, Supplementary Note and Supplementary References. [file ncomms11111-s1.pdf]

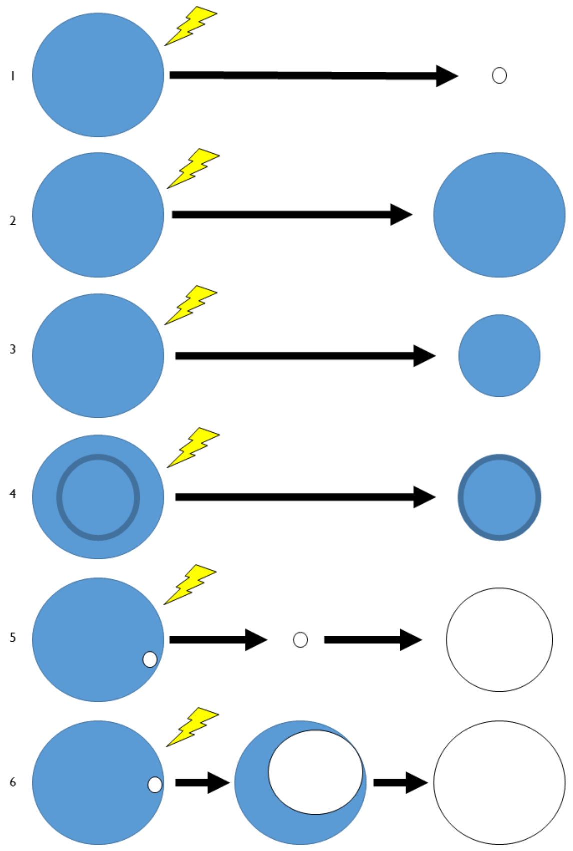

Supplementary Figure 1. Models of tumour response to chemotherapy
Most non-surgical cancer therapies potentially place a massive selection burden on the tumour. Some tumours may exhibit no response, others may respond and relapse, and yet others may show an enduring, perhaps complete, response. Several measures of response exist, based on clinical features, imaging, biomarkers or histology. We can envisage at least 6 ways in which cancers might evolve owing to chemo- or radiotherapy:
(1) complete response;
(2) complete resistance;
(3) tumour shrinkage owing to death of cells in vulnerable states, such as rapid cell cycling
(4) tumour shrinkage owing to the death of intrinsically susceptible cells, leaving a smaller resistant population such as cancer stem cells;
(5) tumour shrinkage as above, initially mimicking a complete response, but with a resistant population growing out from very rare, resistant cells; or
(6) no tumour shrinkage, but owing to the selective pressure, selection of a different sub-clone that replaces the major clone that was present pre-treatment.
Underlying these models are two types of relative Darwinian fitness, that of tumour cells over normal cells and that between tumour sub-clones. In addition, we can in principle distinguish two further types of fitness, namely resistance to therapy and re-growth, although distinguishing these in practice is challenging. To take one example, consider the now classical illustration of model (5), in which a patient's multiple melanoma metastases respond rapidly to targeted BRAF inhibition, followed by an equally regrowth of rapid resistance within those same tumour deposits . This scenario suggests death of many tumour cells followed by re-growth of a resistant clone that no longer has competition from the fitter major clone and can therefore re-populate the tumour niche rapidly. This situation differs importantly from our work in this study, since our patients have received genotoxic agents which are not known to have any predilection for specific mutations or clones.

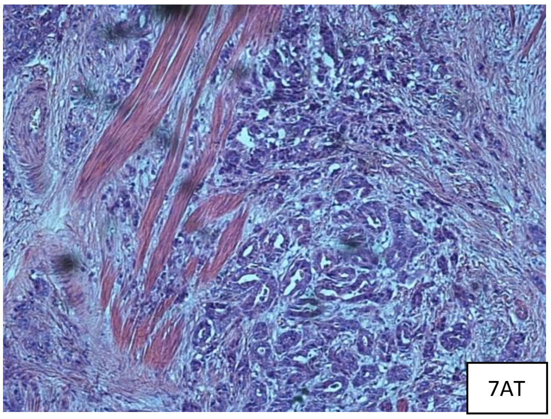

7AT

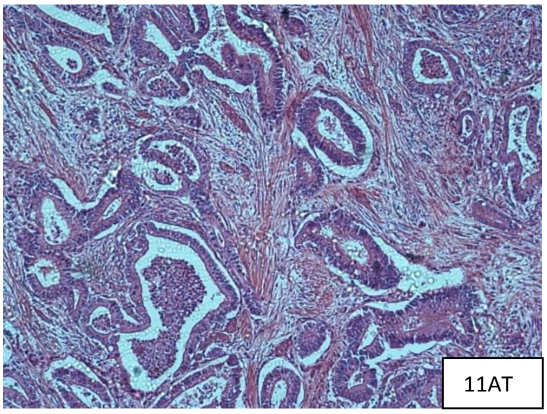

11AT

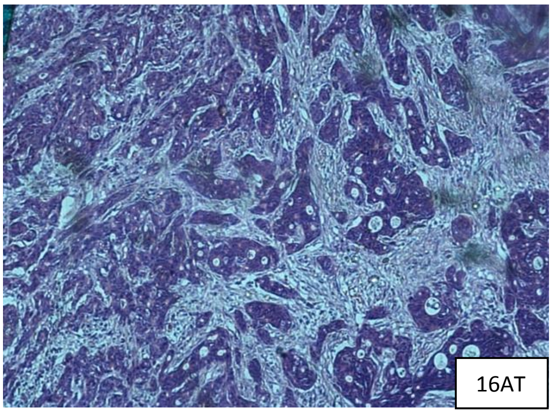

16AT

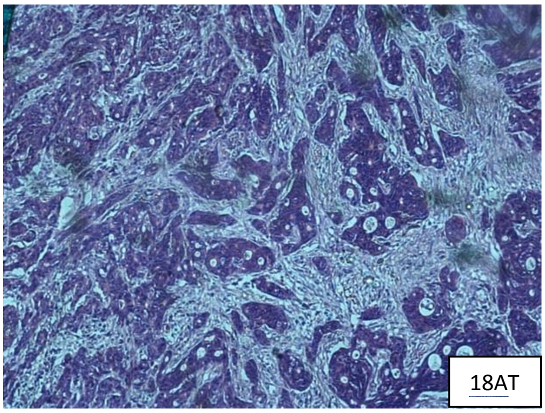

18AT

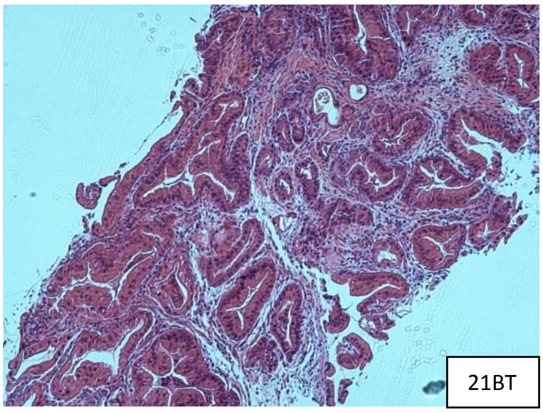

21BT

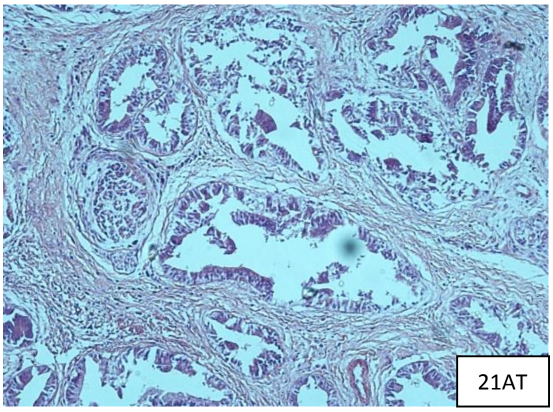

21AT

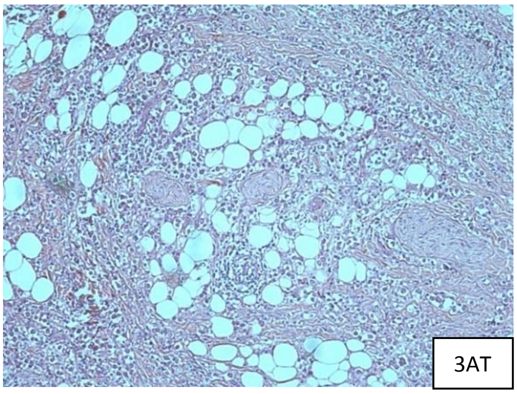

3AT

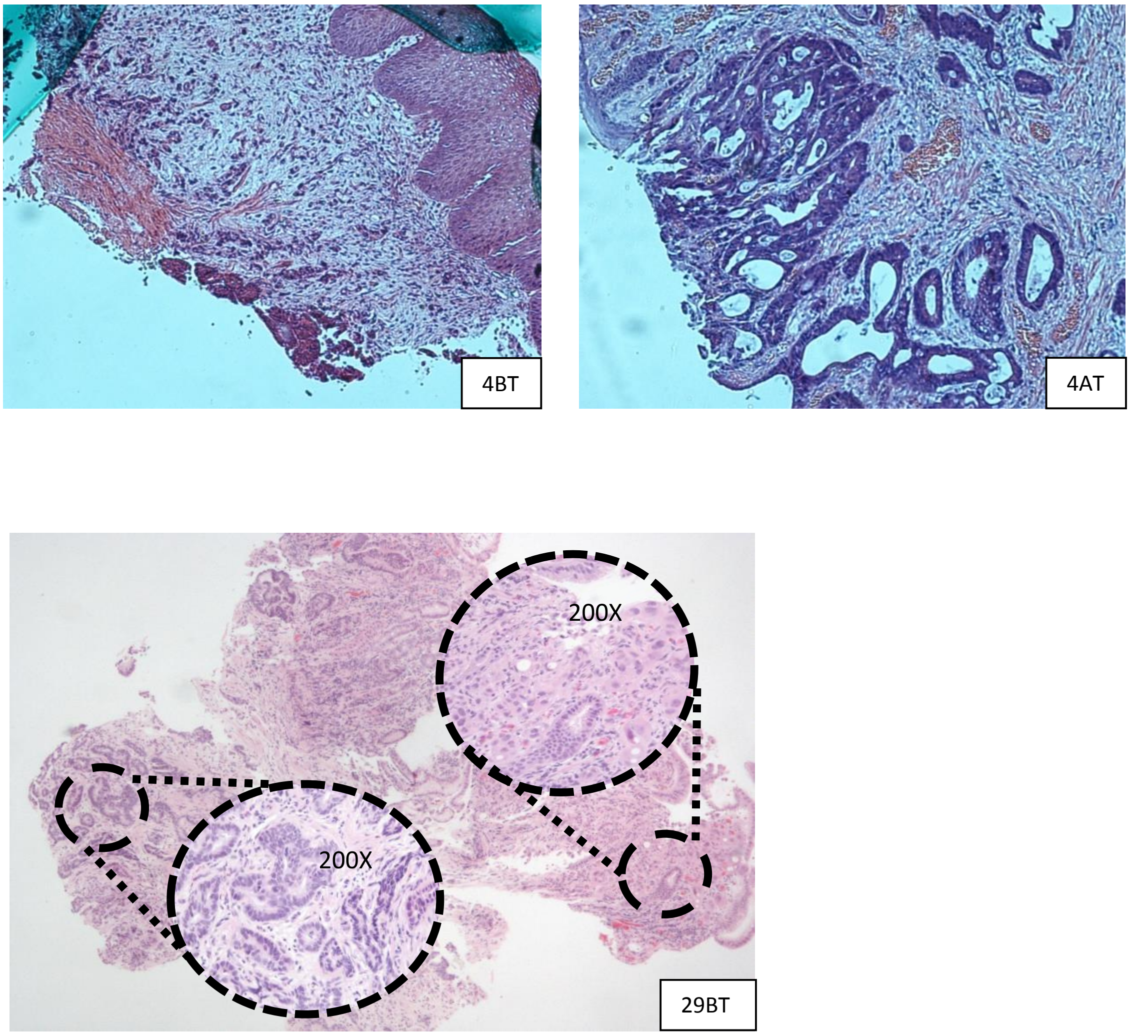

Supplementary Figure 2. Exemplar haematoxylin-and-eosin stained sections of EAC samples analysed in the study. BT=pre-treatment, AT=post-treatment. Views are low power (100X) to demonstrate gross tumour cell content. Bottleneckers (#7, #11) and non-botteneckers (#16, #18, #21) are shown. The areas shown in the Figures are those selected for microenrichment. An example of an excluded specimen with low tumour cell content is shown (post-treatment sample of cancer #3). Cancer #4, a responder, was excluded from the clonal shift analysis on the basis of a low mutation burden in the post-treatment sample, but the histology showing areas of cancer cells underscores the arguably borderline nature of this decision. The cancer also showed a distinct morphological shift with near-complete regression of the poorly differentiated carcinoma cells before treatment, leaving a solid cribriform area post-treatment. The post-treatment sample showed a large decrease (to about 0.5%) in the VAF of a p53 mutation, but actually carried a higher SCNA burden than the pre-treatment sample (Supplementary Figure 6). We suspect that this tumour did pass through a genetic bottleneck, but that the data are insufficient to demonstrate that possibility convincingly. Patient #29's pre-treatment sample is also shown. This case was 29 years old at presentation and had an cancer of unusual morphology. One part of the tumour (left) is moderately differentiated adenocarcinoma with gland formation. The other component (right) has a more diffuse, single-cell growth pattern infiltrating within the lamina propria, and the cells are plump with abundant pink cytoplasm. The two components were analysed together in this study. Of interest, #29 had a p53 mutation at ~50% VAF in his normal oesophageal sample. He was also excluded from the clonal shift analysis owing to low mutation burden in the post-treatment sample, although the pre-treatment mutation burden was also low and the purity decrease after treatment was predicted to be modest. We wonder whether this tumour was driven principally by copy number changes owing to early p53 loss, with the patient's young age accounting in part for the low mutation burden. Moreover, the multi-focal nature of the lesion is suggestive of polyclonality. As for #4, we suspect that the precautionary exclusion of this non-responder patient from the clonal shift analysis might not have been necessary.

(a)

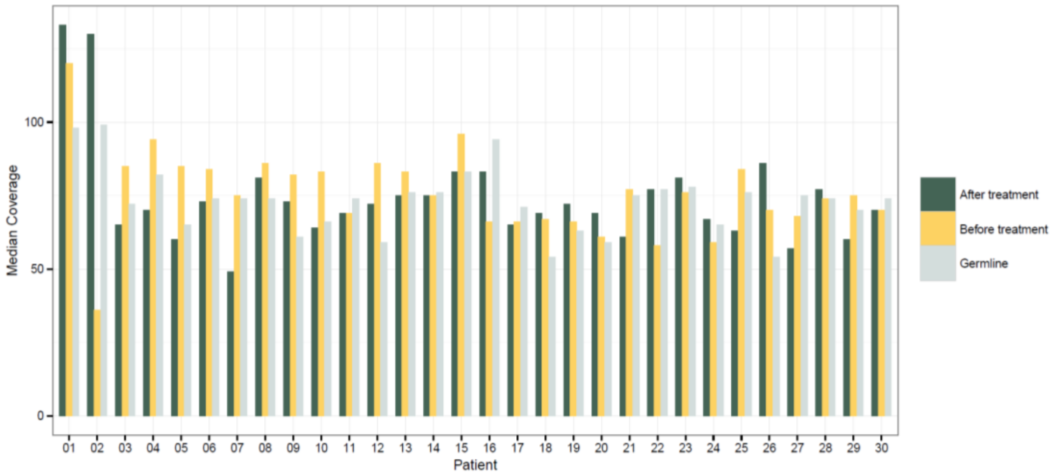
Median Coverage
Patient
After treatment
Before treatment
Germline

(b)

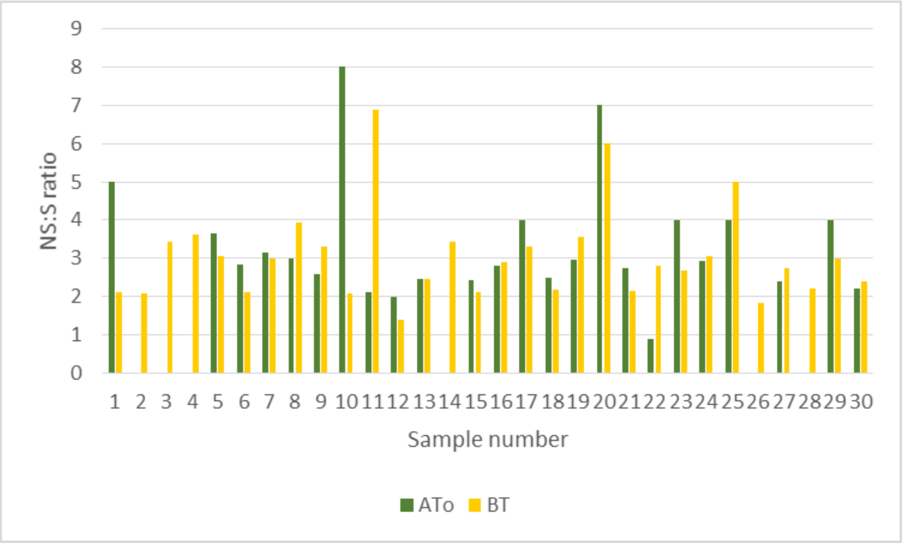
NS:S ratio
Sample number
ATo
BT

(c)

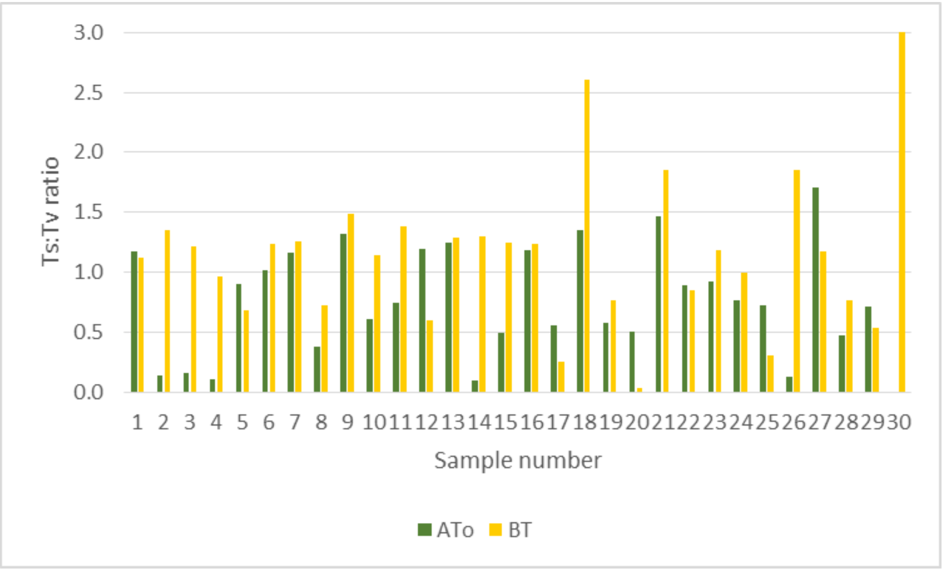
Ts:Tv ratio
Sample number
ATo
BT

(d)

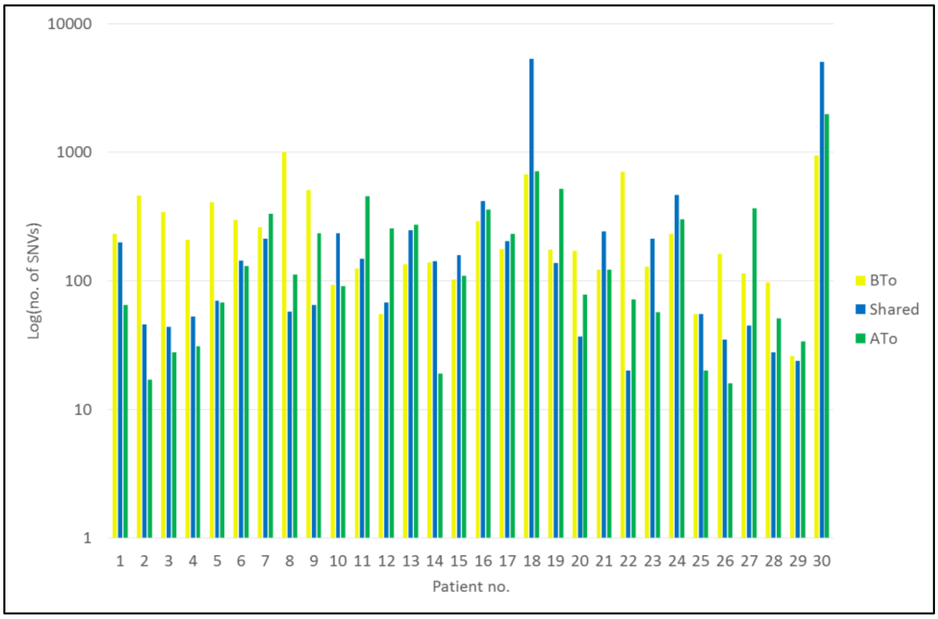

Supplementary Figure 3. Summary statistics for exome sequencing of fresh-frozen samples
(a) Coverage. Median sequencing coverage exome-wide is shown for each sample (green=post-treatment cancer, yellow=post-treatment cancer, grey=pre-treatment normal tissue). In almost all samples, ~50% of the exome was covered at >100X. Overall, median sequencing coverage was 74X (range 36-133). Patients 1-6 and 9-12 were analysed using the Nextera capture system, whereas other patients were analysed using TruSeq. The median sequencing coverage did not differ significantly between these methods (P=0.29, Wilcoxon test). There was also no significant difference in median read depth between responders and non-responders or in the origin of samples from normal tissue, cancer pre-treatment or cancer post-treatment (P=0.13 and P=0.76, Anova). Specifically, paired pre- and post-treatment sample sequencing coverage did not differ significantly (P=0.24, paired Wilcoxon test).
(b) Non-synonymous:synonymous SNV ratios. Ratios are shown in combined pre-treatment and shared (BT) and post-treatment-specific (ATo) samples. Absent data represent samples in which few mutations occurred and ratios were based on too few data points for useful analysis.
(c) Transition:transversion ratios. Ratios are shown in combined pre-treatment and shared (BT) and post-treatment-specific (ATo) samples. Ratios were significantly higher for pre-treatment/shared mutations (P=0.0148, paired Wilcoxon test), reflecting decrease in C>T changes in post-treatment samples, but there was no association with response (data not shown).
(d) SNV counts in each sample. Counts are derived from variants present only in the pre-treatment sample (BTo, yellow), in both the pre- and post-treatment samples (Shared, blue) and only in the post-treatment sample (ATo, green). Note the high burdens in the MSI+ samples #18 and #30. Mutations are not filtered for location within the exome or elsewhere in the genome.

(a)

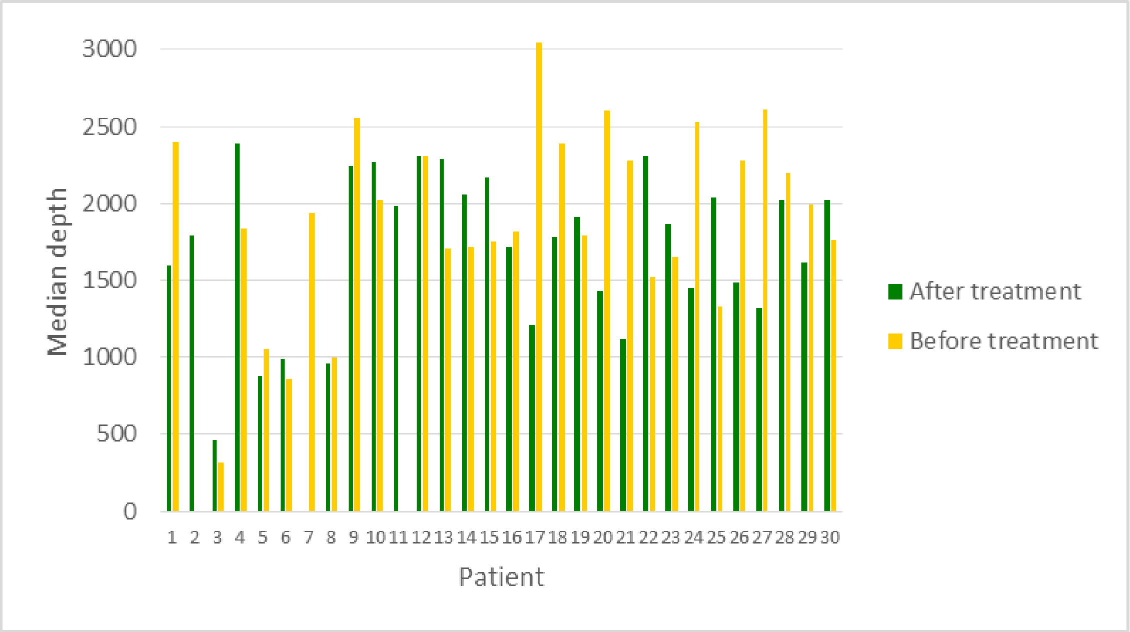
3000
2500
2000
1500
1000
500
0
Median depth
1 2 3 4 5 6 7 8 9 10 11 12 13 14 15 16 17 18 19 20 21 22 23 24 25 26 27 28 29 30
Patient
After treatment
Before treatment

(b)

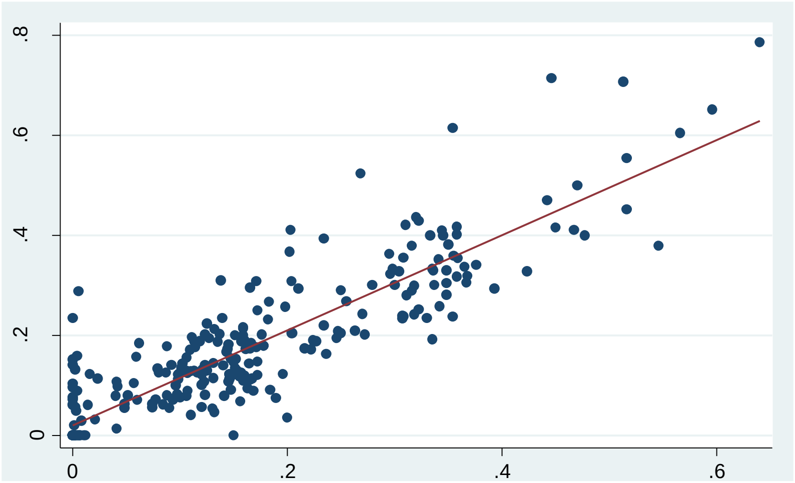
0
.2
.4
.6
.8
0
.2
.4
.6

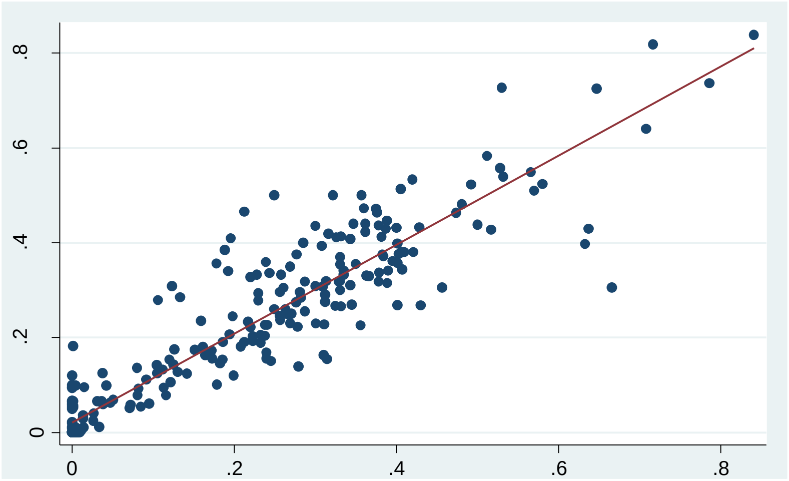
0
.2
.4
.6
.8
0
.2
.4
.6
.8

Supplementary Figure 4. Ampliseq data from fresh-frozen samples
(a) Median depth of sequencing using the Ampliseq comprehensive cancer panel is shown for each sample. For a small number of cancers, lack of sample precluded technical validation of exome data in this way. Somatic mutations in the following genes present on the comprehensive cancer panel arrays were found previously in the exome sequencing data: AFF3, LRP1B, AKAP9, **NOTCH1**, THBS1, **TP53**, GNAS, BTK, ALK, RPS6KA2, GUCY1A2, CDK4, PIM1, PKHD1, SYNE1, PTPN11, TGM7, ERG, **ARID1A**, FLT4, RET, AKT1, IL7R, CARD11, MET, CSMD3, FGFR2, **STAG2**, SF3B1, **CDKN2A**, ERBB3, ZNF521, **SMARCA4**, SOX11, RECQL4, IRS2, **CNTNAP5**, SOX2, GDNF, EPHA7, CDH1, **SMAD4**, MYH9, DPYD, ERBB4, CTNNA1, TRRAP, BRAF, NTRK3, RNF213, PTPRT, G6PD, ARNT, CDC73, CDK12, RNF2, BCL6, **FBXW7**, SEMA5A, SMARCB1, TAF1, HIF1A, ERBB2, ASXL1, PIK3CG, CDH11, PPP2R1A, **PIK3CA**, KIT, CDH2, PIK3C2B, ERCC3, FLCN, SETD2, APC, GRM8, EXT1, EP400, LAMP1, CIC, NF2, EPHB1, CASC5, BRIP1, MPL, NOTCH2, **CTNNB1**, MITF, DST, ROS1, IGF2R, EGFR, KAT6A, PTCH1, KAT6B, BMPR1A, TCF7L2, MAML2, ARID2, NKX2-1, CREBBP, ERCC4, MYH11, CDH5, ETV4, ITGB3, SEPT09, MBD1, KEAP1, TBX22, MTR, EZH2, TET1, MYO18B, PTEN, PCDH9, NIN, PIK3R1, SAMD9, FANCF, DCC, BAI3, RUNX1T1, PTPRD, MAP2K1, XPO1, ABL2, MRE11A, RB1, PDGFRA, FANCG, XPA, CDK8, TCF3, NUP98, AR, TNFRSF14, PIK3CD, PAX7, CMPK1, PDE4DIP, RNASEL, PAX8, XPC, ITGA9, GATA2, WHSC1, AFF1, LIFR, FGFR4, NOTCH4, DAXX, MYB, CDK6, EPHB6, IKBKB, PRKDC, NCOA2, UBR5, TAF1L, PAX5, RALGDS, MEN1, NUMA1, ATM, FLI1, **KRAS**, BCL11B, BUB1B, PML, SOCS1, PER1, COL1A1, PIK3R2, PLCG1, ITGB2, TIMP3, GATA1. Median coverage obtained for these mutations was 1737x (range 259-4035x). Bold text indicates probable EAC driver genes.
(b) The plots show (upper) VAFs of SNVs present in the pre-treatment samples and (lower), VAFs of SNVs present in the post-treatment samples, with the Ampliseq data on the x-axis and exome sequencing data on the y-axis. In both cases, there was strong concordance (r^2=0.80, slope=0.95 and r^2=0.87, slope=0.94 respectively).

(a)

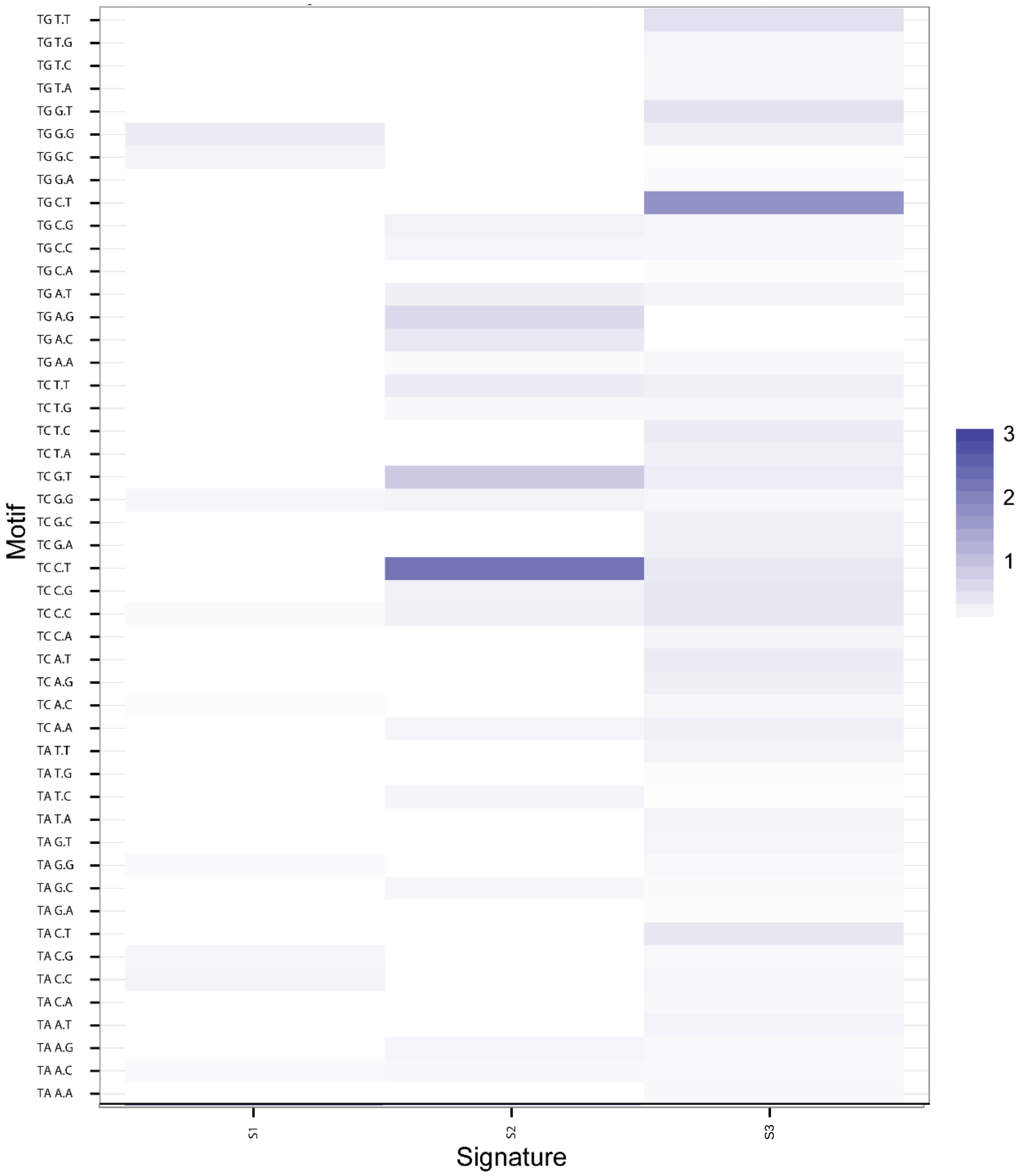
TG T.T
TG T.G
TG T.C
TG T.A
TG G.T
TG G.G
TG G.C
TG G.A
TG C.T
TG C.G
TG C.C
TG C.A
TG A.T
TG A.G
TG A.C
TG A.A
TC T.T
TC T.G
TC T.C
TC T.A
TC G.T
TC G.G
TC G.C
TC G.A
TC C.T
TC C.G
TC C.C
TC C.A
TC A.T
TC A.G
TC A.C
TC A.A
TA T.T
TA T.G
TA T.C
TA T.A
TA G.T
TA G.G
TA G.C
TA G.A
TA C.T
TA C.G
TA C.C
TA C.A
TA A.T
TA A.G
TA A.C
TA A.A
Motif
S1
S2
S3
Signature
3
2
1

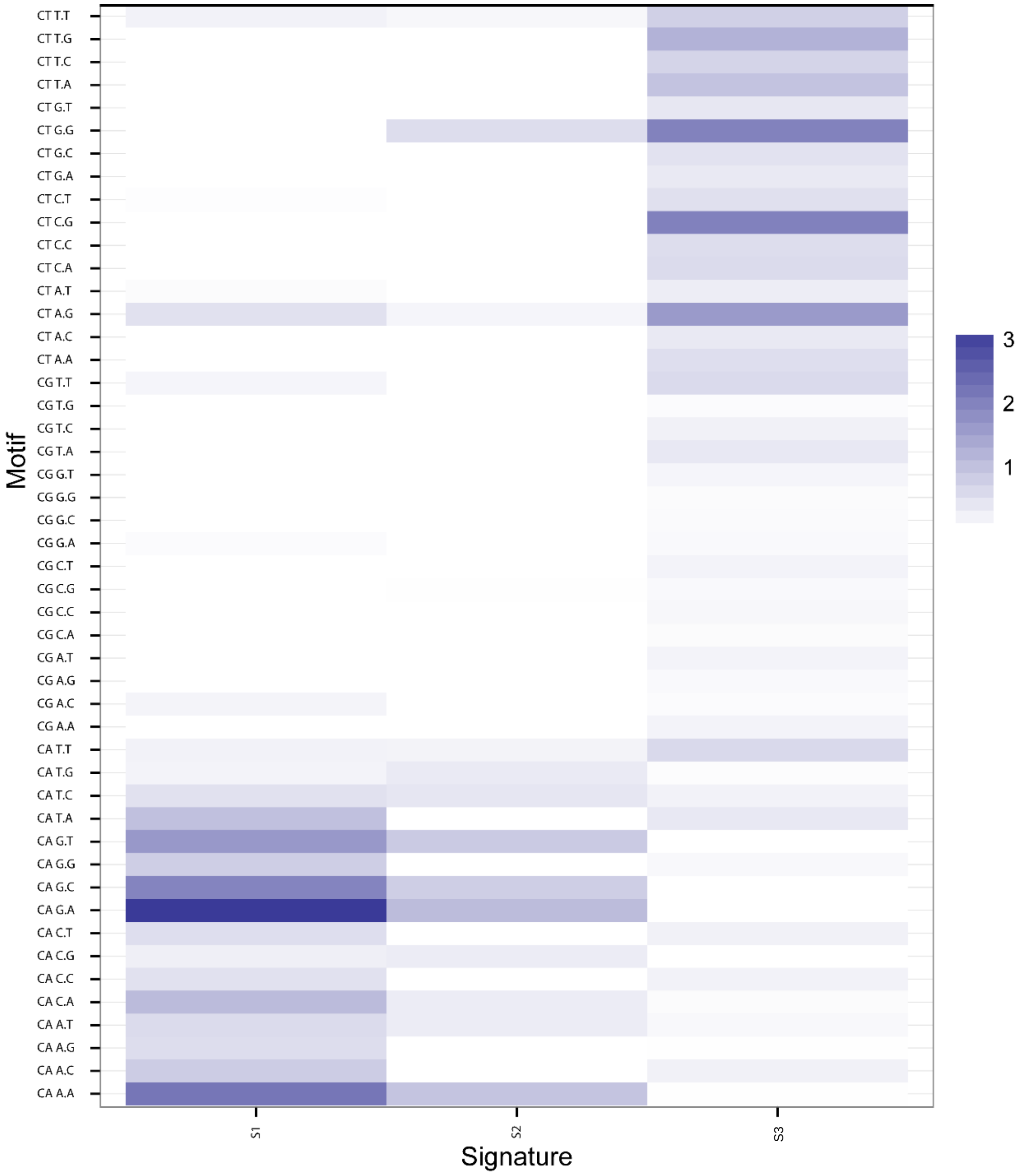

CT T.T
CT T.G
CT T.C
CT T.A
CT G.T
CT G.G
CT G.C
CT G.A
CT C.T
CT C.G
CT C.C
CT C.A
CT A.T
CT A.G
CT A.C
CT A.A
CG T.T
CG T.G
CG T.C
CG T.A
CG G.T
CG G.G
CG G.C
CG G.A
CG C.T
CG C.G
CG C.C
CG C.A
CG A.T
CG A.G
CG A.C
CG A.A
CA T.T
CA T.G
CA T.C
CA T.A
CA G.T
CA G.G
CA G.C
CA G.A
CA C.T
CA C.G
CA C.C
CA C.A
CA A.T
CA A.G
CA A.C
CA A.A
Motif
S1
S2
S3
Signature
3
2
1

(b)

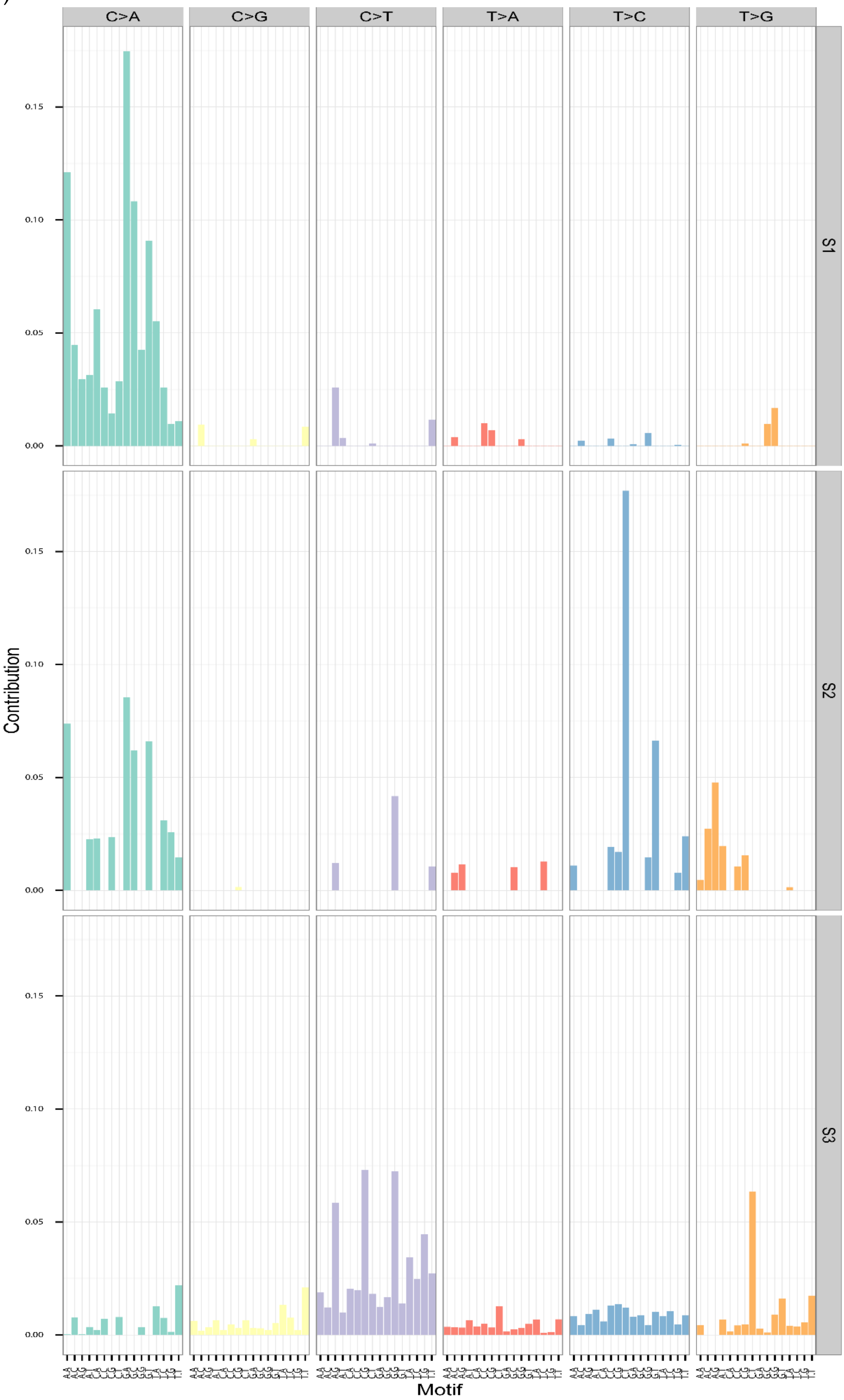

(c)

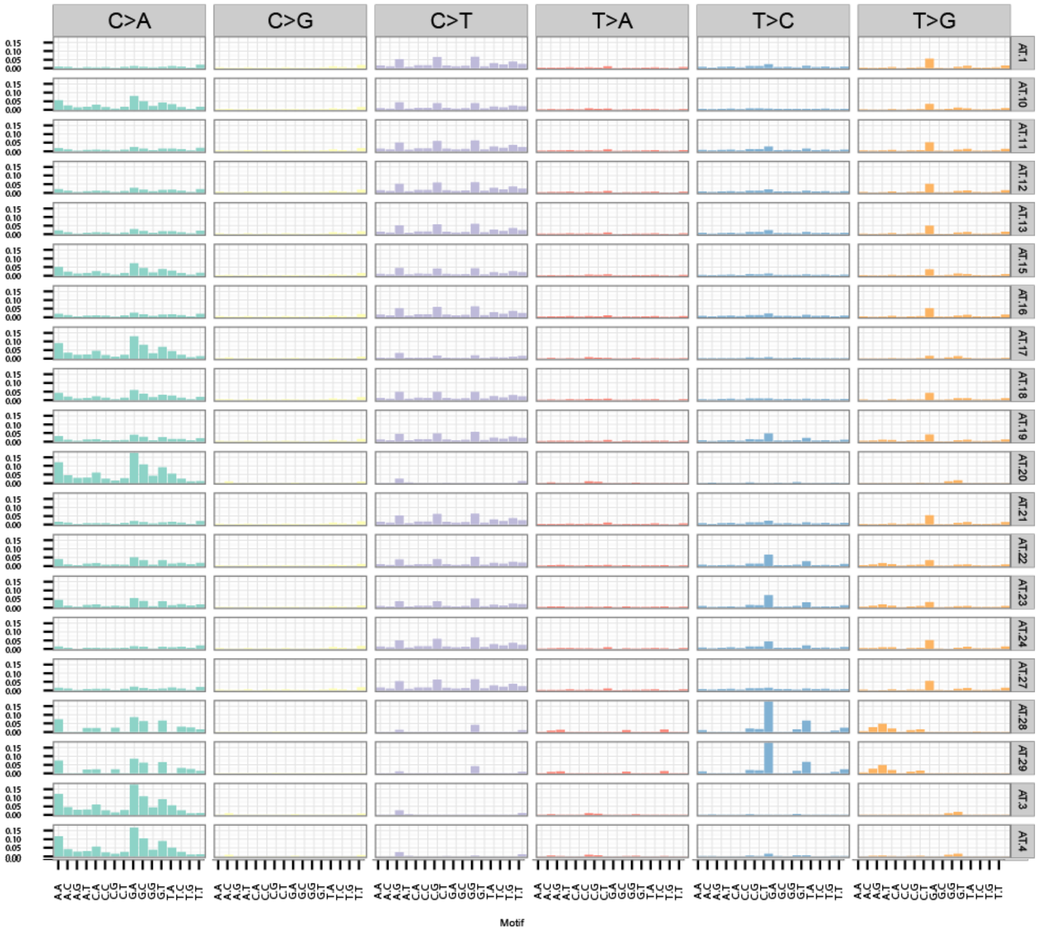
C>A
C>G
C>T
T>A
T>C
T>G
AT.1
AT.10
AT.11
AT.12
AT.13
AT.15
AT.16
AT.17
AT.18
AT.19
AT.20
AT.21
AT.22
AT.23
AT.24
AT.27
AT.28
AT.29
AT.3
AT.4
0.15
0.10
0.05
0.00
Motif

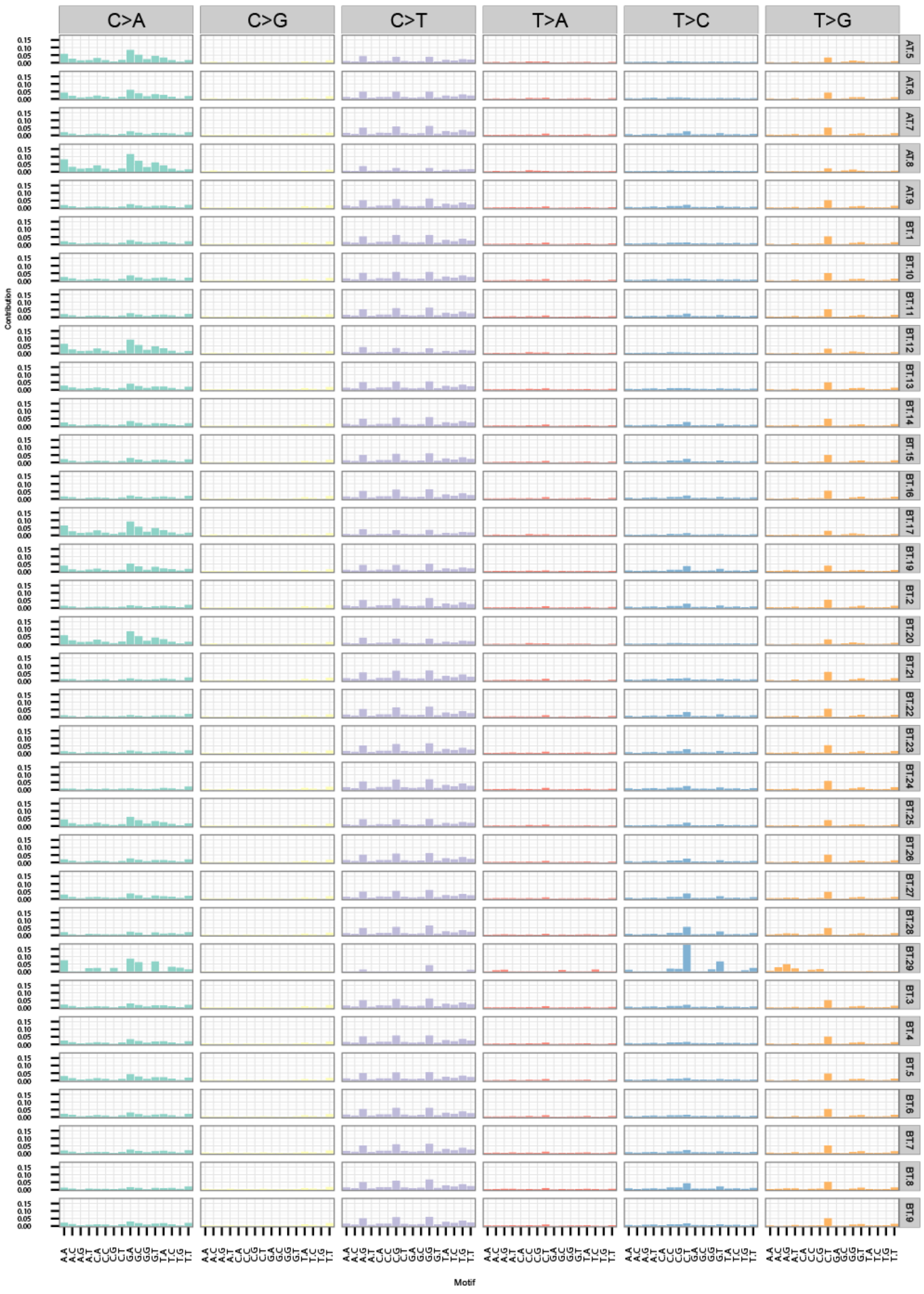

C>A
C>G
C>T
T>A
T>C
T>G
Contribution
Motif
AT.5
AT.6
AT.7
AT.8
AT.9
BT.1
BT.10
BT.11
BT.12
BT.13
BT.14
BT.15
BT.16
BT.17
BT.19
BT.2
BT.20
BT.21
BT.22
BT.23
BT.24
BT.25
BT.26
BT.27
BT.28
BT.29
BT.3
BT.4
BT.5
BT.6
BT.7
BT.8
BT.9

(d)

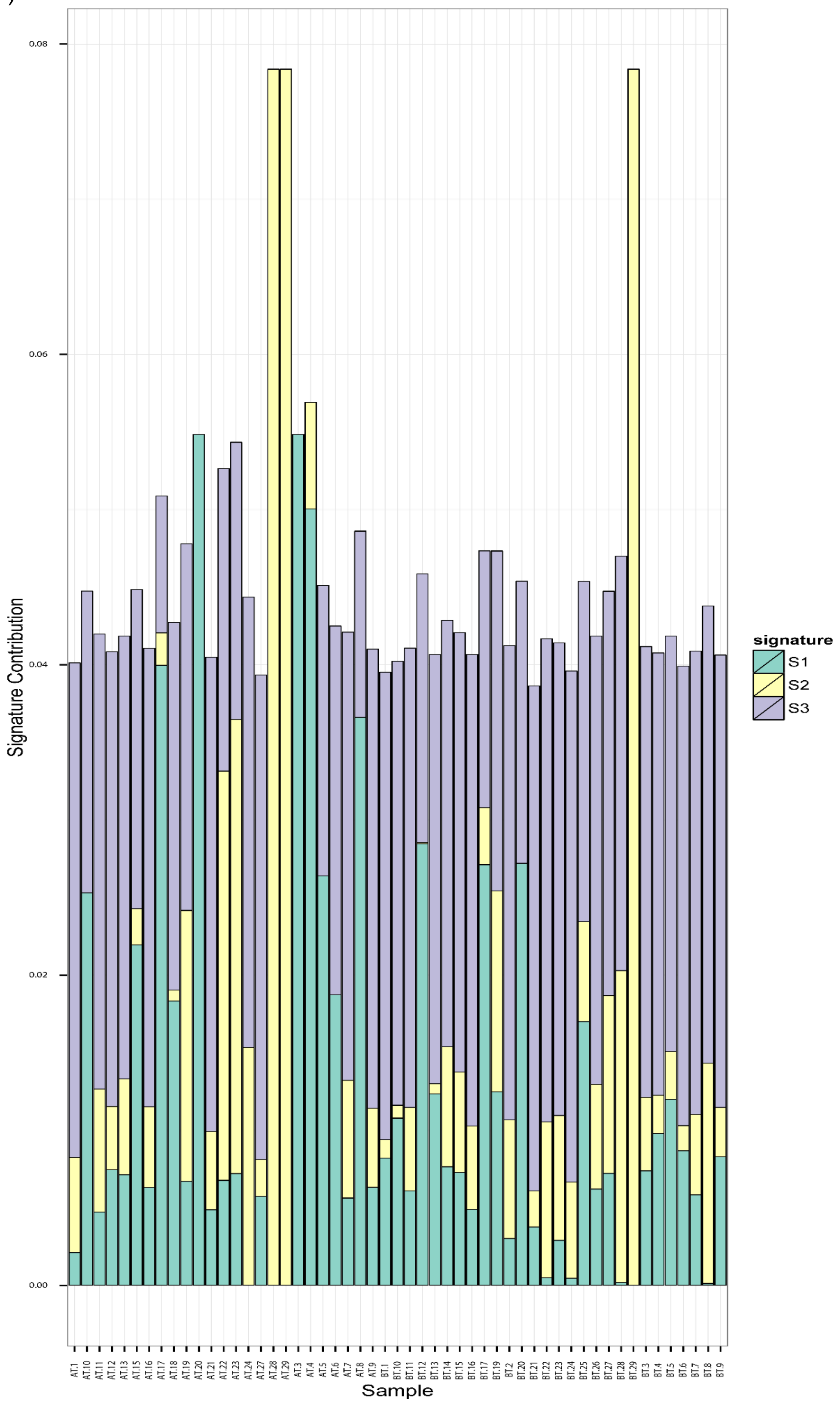

Supplementary Figure 5. Mutation spectra and signatures
(a) Heatmap of the contribution of each SNV in trinucleotide context (96-channels) to the 3 mutational signatures derived from pre- and post-treatment samples with more than 20 SNVs in total. (b) Representation of the data in (a) in bar chart form. (c) Mutation spectrum of each pre-treatment (BT) and post-treatment (AT) sample in 96-channel format. (d) Relative contributions of mutational signatures to each sample

(a)

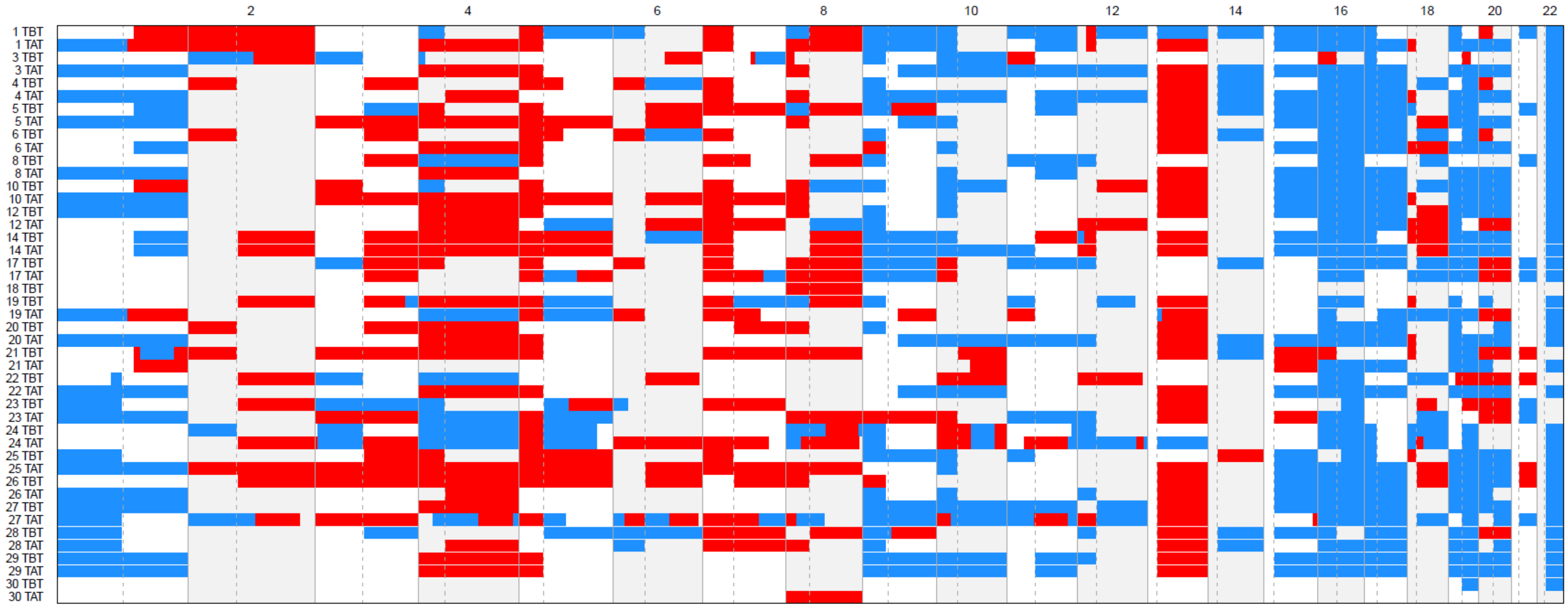

2
4
6
8
10
12
14
16
18
20
22
1 TBT
1 TAT
3 TBT
3 TAT
4 TBT
4 TAT
5 TBT
5 TAT
6 TBT
6 TAT
8 TBT
8 TAT
10 TBT
10 TAT
12 TBT
12 TAT
14 TBT
14 TAT
17 TBT
17 TAT
18 TBT
19 TBT
19 TAT
20 TBT
20 TAT
21 TBT
21 TAT
22 TBT
22 TAT
23 TBT
23 TAT
24 TBT
24 TAT
25 TBT
25 TAT
26 TBT
26 TAT
27 TBT
27 TAT
28 TBT
28 TAT
29 TBT
29 TAT
30 TBT
30 TAT
1
3
5
7
9
11
13
15
17
19
21

(b)

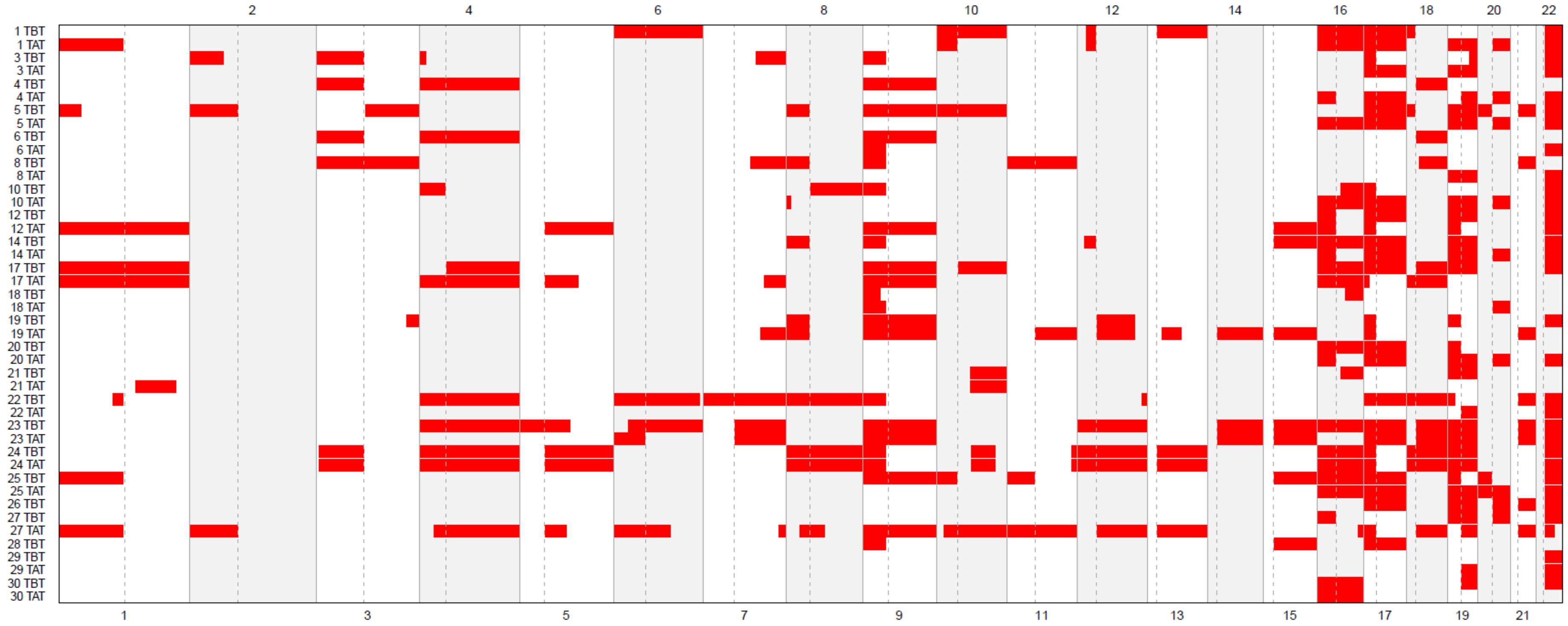
2
4
6
8
10
12
14
16
18
20
22
1 TBT
1 TAT
3 TBT
3 TAT
4 TBT
4 TAT
5 TBT
5 TAT
6 TBT
6 TAT
8 TBT
8 TAT
10 TBT
10 TAT
12 TBT
12 TAT
14 TBT
14 TAT
17 TBT
17 TAT
18 TBT
18 TAT
19 TBT
19 TAT
20 TBT
20 TAT
21 TBT
21 TAT
22 TBT
22 TAT
23 TBT
23 TAT
24 TBT
24 TAT
25 TBT
25 TAT
26 TBT
27 TBT
27 TAT
28 TBT
29 TBT
29 TAT
30 TBT
30 TAT
1
3
5
7
9
11
13
15
17
19
21

(c)

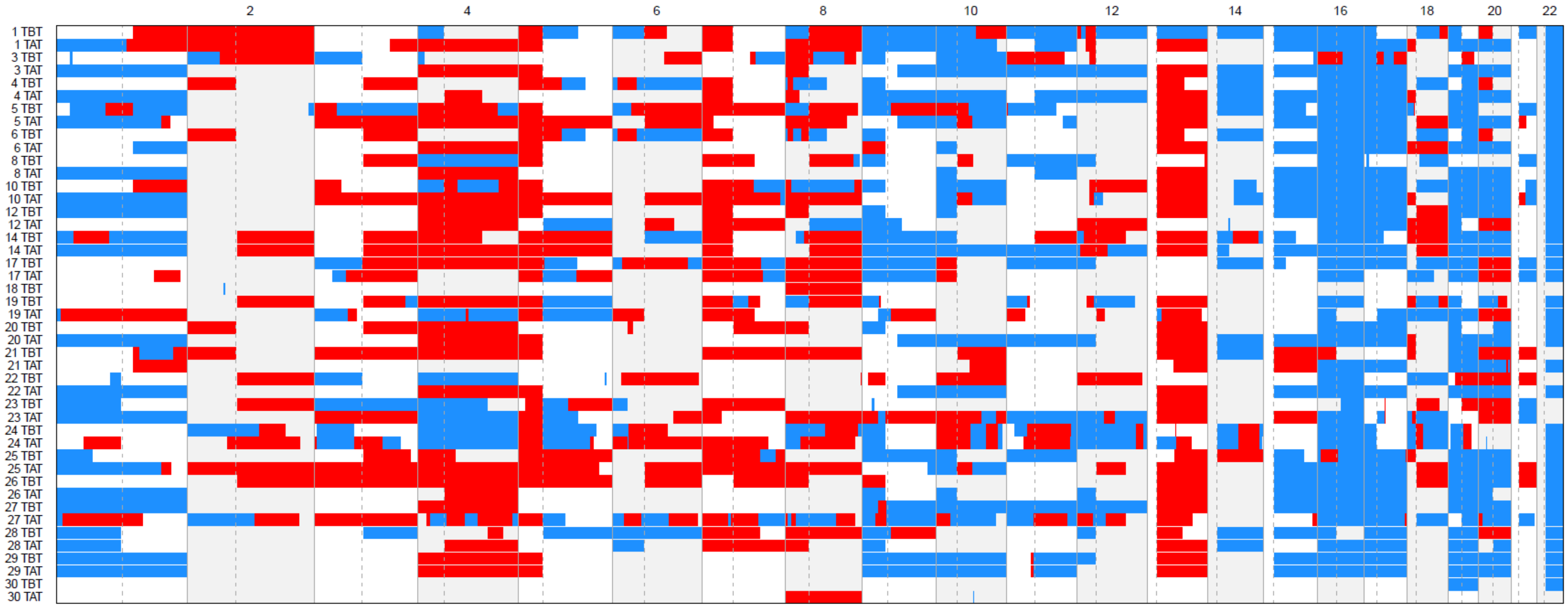

2
4
6
8
10
12
14
16
18
20
22
1 TBT
1 TAT
3 TBT
3 TAT
4 TBT
4 TAT
5 TBT
5 TAT
6 TBT
6 TAT
8 TBT
8 TAT
10 TBT
10 TAT
12 TBT
12 TAT
14 TBT
14 TAT
17 TBT
17 TAT
18 TBT
19 TBT
19 TAT
20 TBT
20 TAT
21 TBT
21 TAT
22 TBT
22 TAT
23 TBT
23 TAT
24 TBT
24 TAT
25 TBT
25 TAT
26 TBT
26 TAT
27 TBT
27 TAT
28 TBT
28 TAT
29 TBT
29 TAT
30 TBT
30 TAT
1
3
5
7
9
11
13
15
17
19
21

(d)

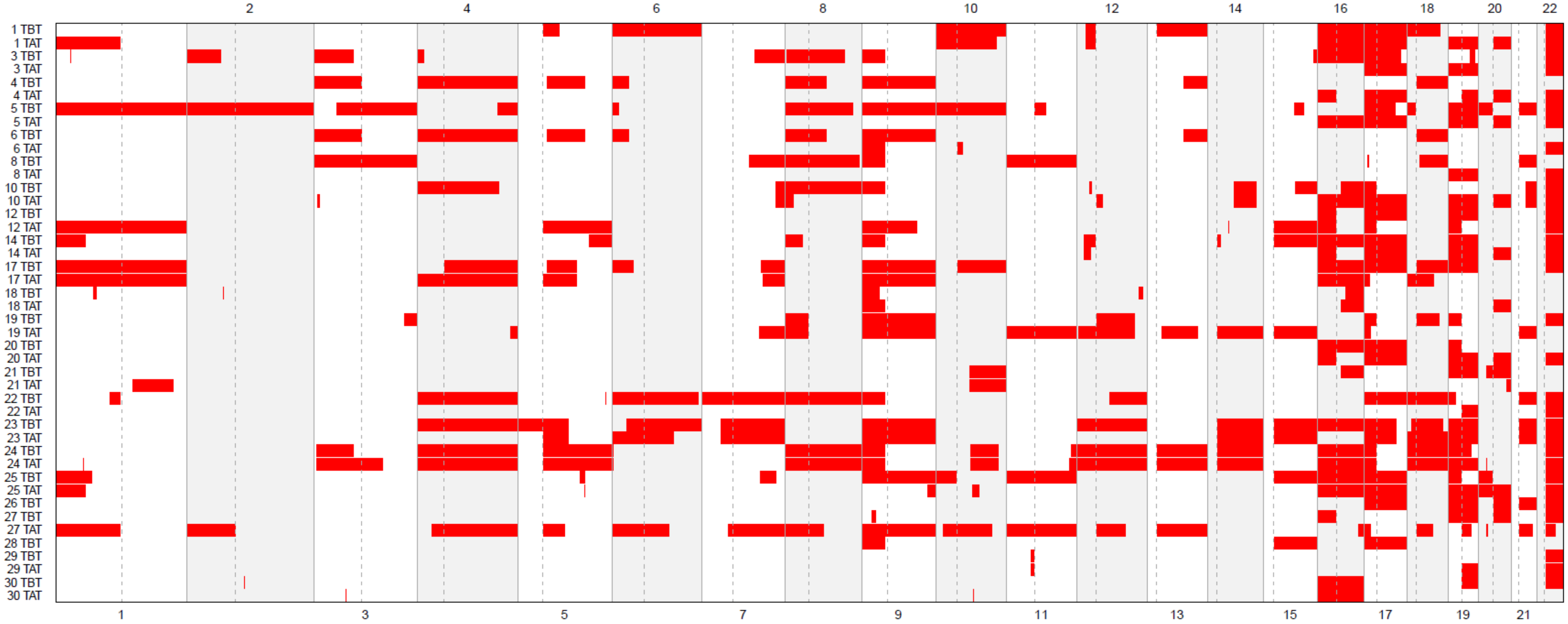
2
4
6
8
10
12
14
16
18
20
22
1 TBT
1 TAT
3 TBT
3 TAT
4 TBT
4 TAT
5 TBT
5 TAT
6 TBT
6 TAT
8 TBT
8 TAT
10 TBT
10 TAT
12 TBT
12 TAT
14 TBT
14 TAT
17 TBT
17 TAT
18 TBT
18 TAT
19 TBT
19 TAT
20 TBT
20 TAT
21 TBT
21 TAT
22 TBT
22 TAT
23 TBT
23 TAT
24 TBT
24 TAT
25 TBT
25 TAT
26 TBT
27 TBT
27 TAT
28 TBT
29 TBT
29 TAT
30 TBT
30 TAT
1
3
5
7
9
11
13
15
17
19
21

(e)

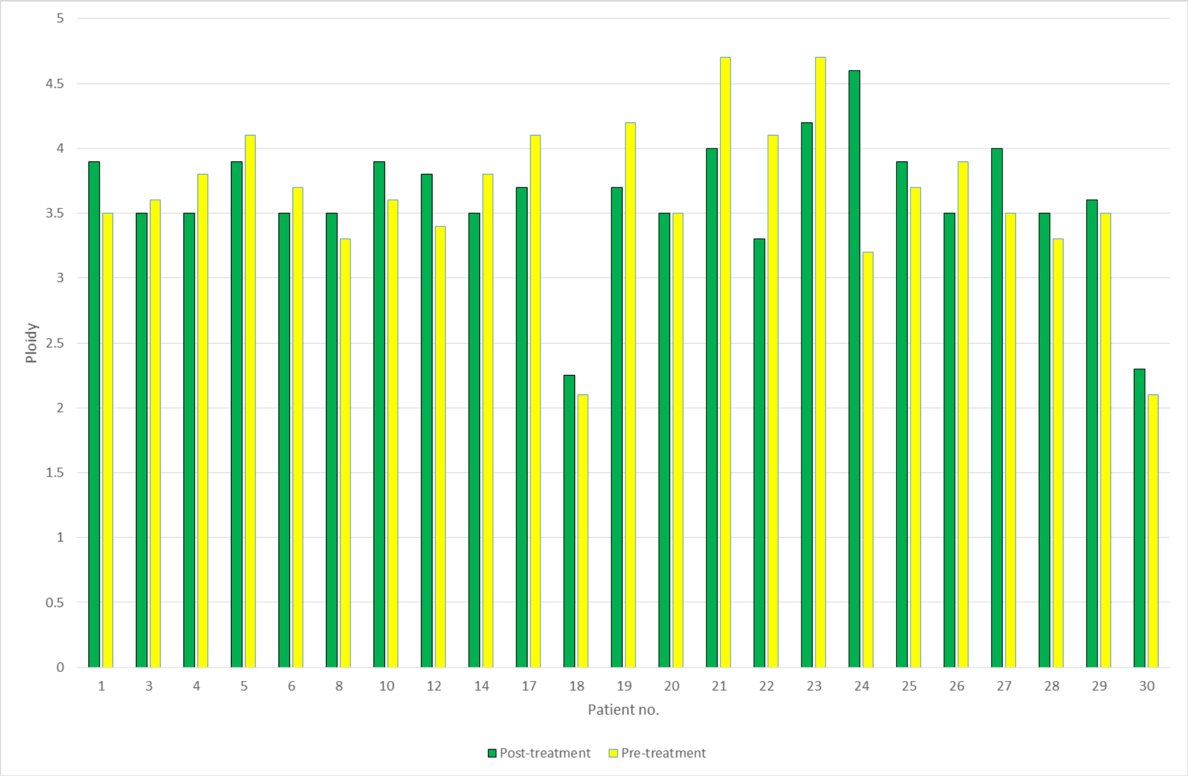

Supplementary Figure 6. Somatic copy number alterations, LOH and ploidy
(a) OncoSNP SCNAs rank 1 (highest confidence) copy number output. Red=gain, blue=deletion.
(b) OncoSNP rank 1 LOH output
(c) OncoSNP all ranks copy number output. Focal copy number changes are not shown for reasons of pictorial resolution, but are listed in Supplementary Table 5.
(d) OncoSNP all ranks LOH output
(e) OncoSNP estimates of ploidy for paired samples. The measure shown is average copy number across the genome.

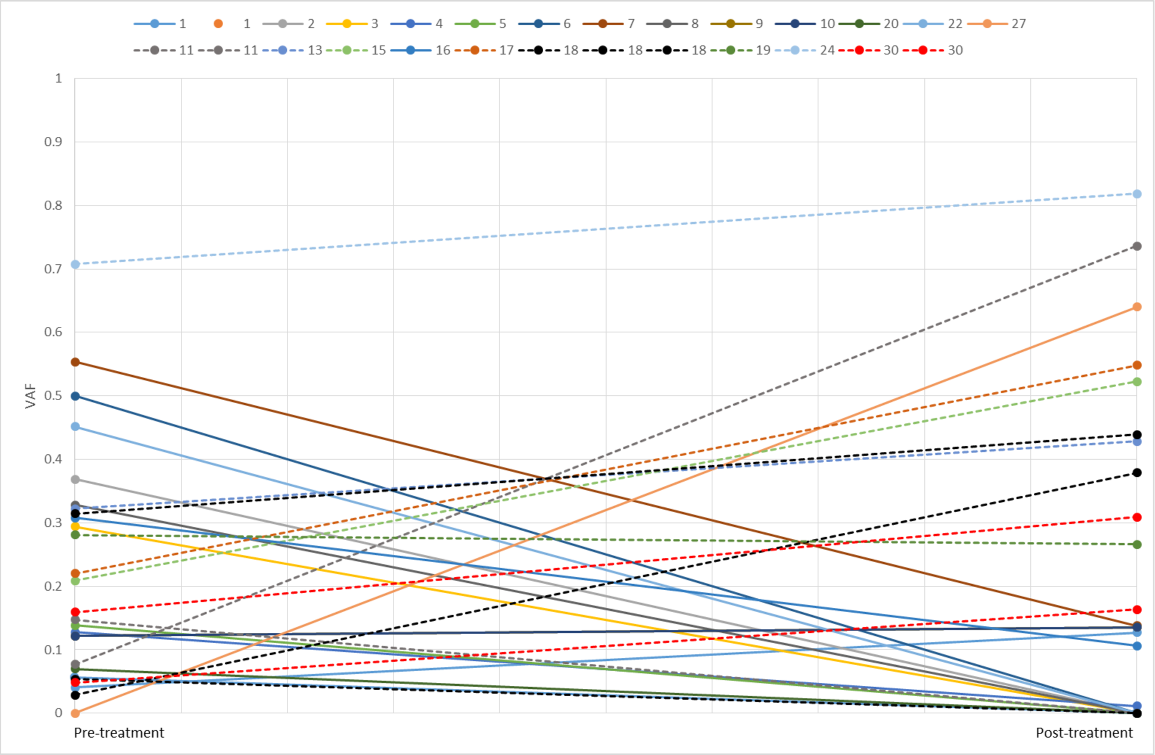

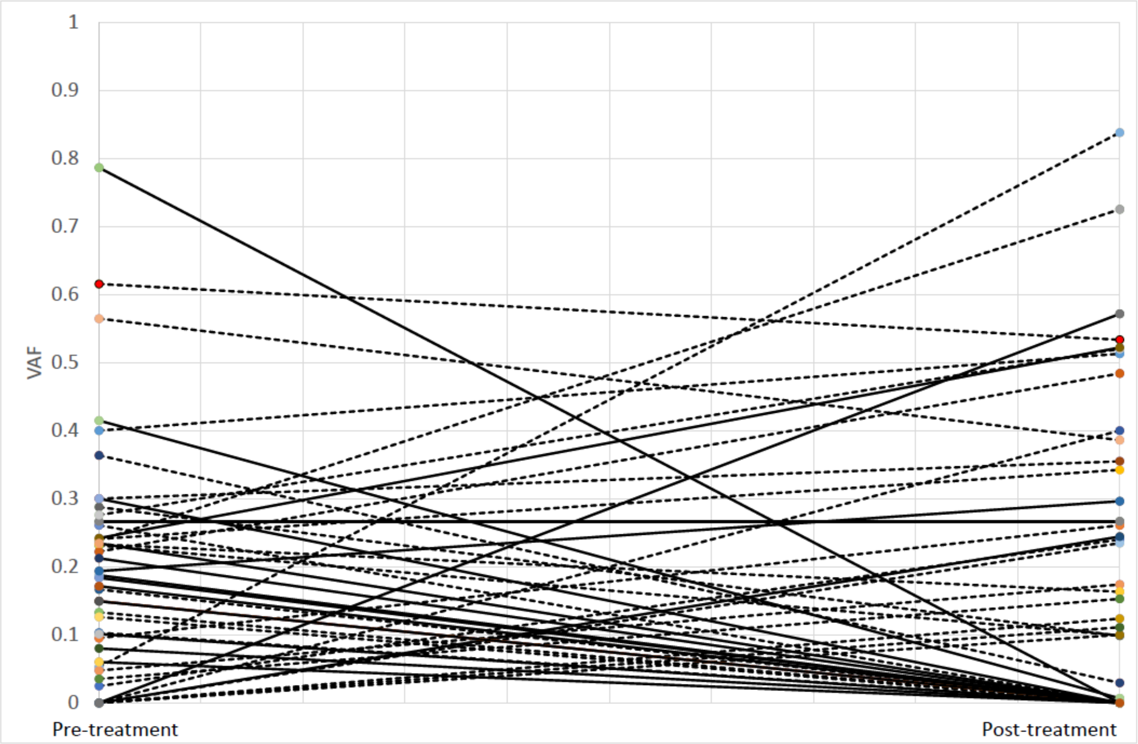

Supplementary Figure 7. Changes in p53 (upper) and other driver mutation (lower) VAF in each cancer between pre-treatment (left axis) and post-treatment (right axis) samples from exome data.
Each line represents a mutation. Responders are shown by solid lines, and non-responders by dashed lines. For p53, case number is shown above; some cancers had more than one mutation. Note that these VAFs are not corrected for tumour purity or ploidy. Cancers subsequently excluded from the analysis of clonal evolution after therapy (#2, #3, #4, #20, #26, #29) remain in this Figure for the sake of completeness.

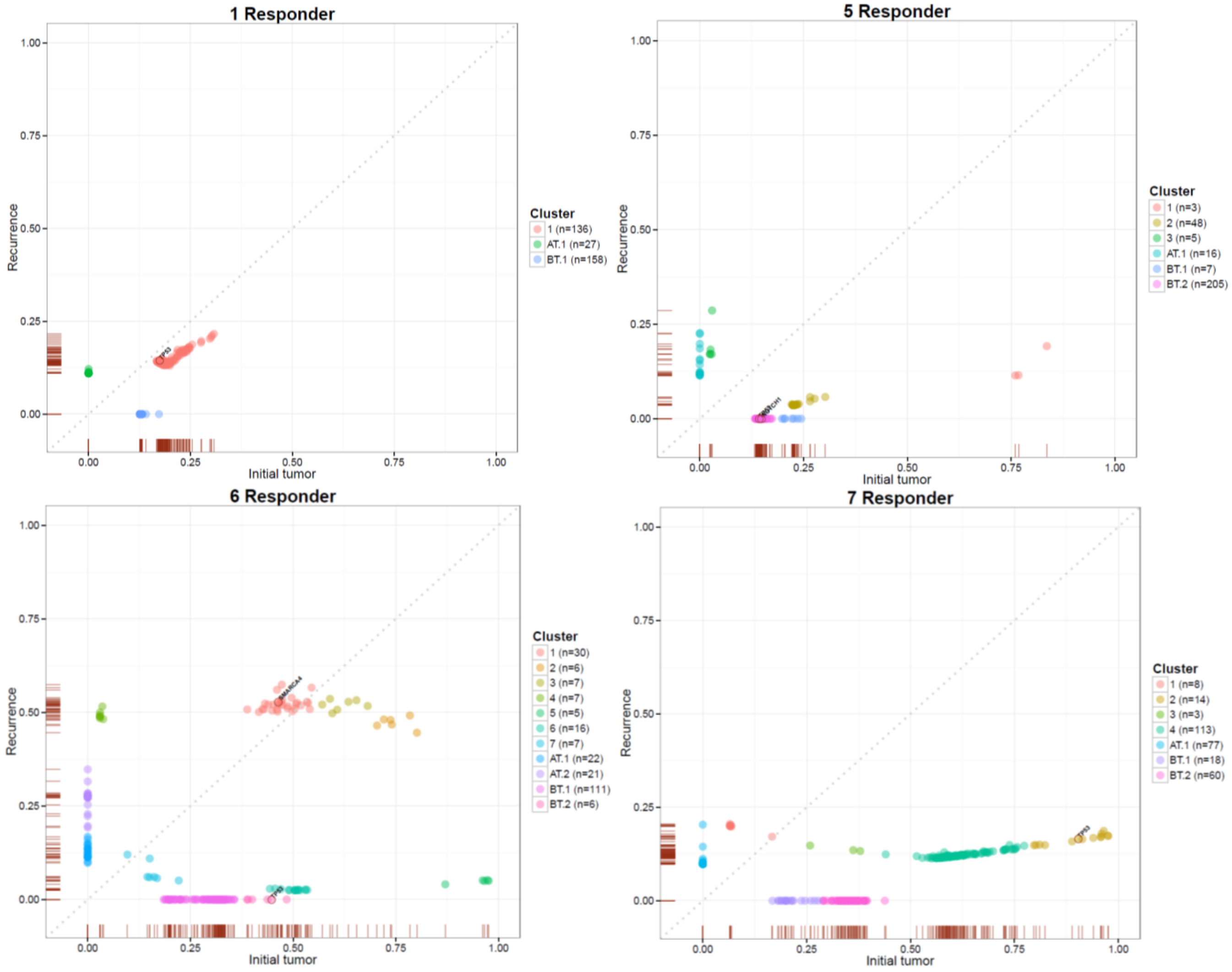
1 Responder
Recurrence
Initial tumor
Cluster
1 (n=136)
AT.1 (n=27)
BT.1 (n=158)
5 Responder
Recurrence
Initial tumor
Cluster
1 (n=3)
2 (n=48)
3 (n=5)
AT.1 (n=16)
BT.1 (n=7)
BT.2 (n=205)
6 Responder
Recurrence
Initial tumor
Cluster
1 (n=30)
2 (n=6)
3 (n=7)
4 (n=7)
5 (n=5)
6 (n=16)
7 (n=7)
AT.1 (n=22)
AT.2 (n=21)
BT.1 (n=111)
BT.2 (n=6)
7 Responder
Recurrence
Initial tumor
Cluster
1 (n=8)
2 (n=14)
3 (n=3)
4 (n=113)
AT.1 (n=77)
BT.1 (n=18)
BT.2 (n=60)

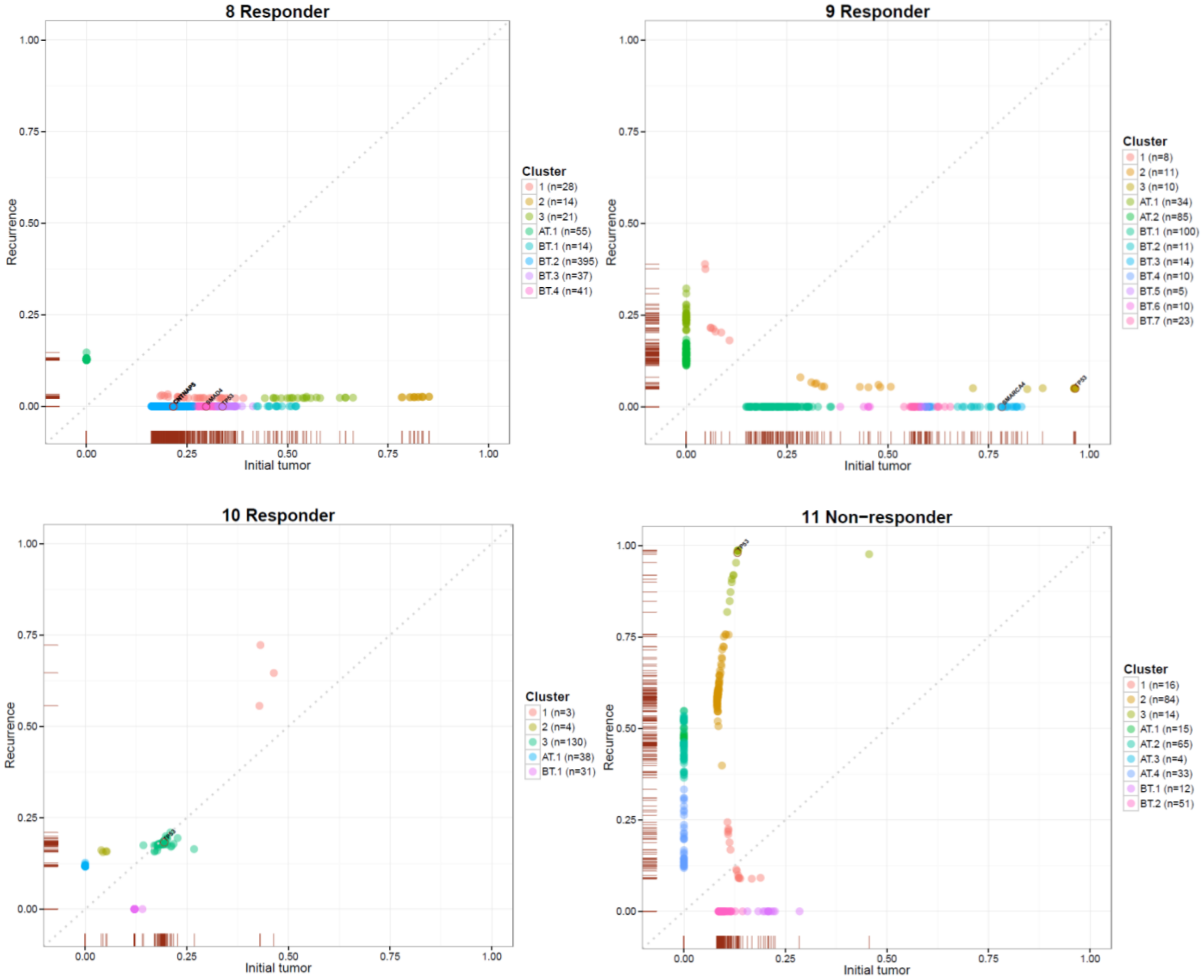

8 Responder
Recurrence
Initial tumor
Cluster
1 (n=28)
2 (n=14)
3 (n=21)
AT.1 (n=55)
BT.1 (n=14)
BT.2 (n=395)
BT.3 (n=37)
BT.4 (n=41)
9 Responder
Recurrence
Initial tumor
Cluster
1 (n=8)
2 (n=11)
3 (n=10)
AT.1 (n=34)
AT.2 (n=85)
BT.1 (n=100)
BT.2 (n=11)
BT.3 (n=14)
BT.4 (n=10)
BT.5 (n=5)
BT.6 (n=10)
BT.7 (n=23)
10 Responder
Recurrence
Initial tumor
Cluster
1 (n=3)
2 (n=4)
3 (n=130)
AT.1 (n=38)
BT.1 (n=31)
11 Non−responder
Recurrence
Initial tumor
Cluster
1 (n=16)
2 (n=84)
3 (n=14)
AT.1 (n=15)
AT.2 (n=65)
AT.3 (n=4)
AT.4 (n=33)
BT.1 (n=12)
BT.2 (n=51)

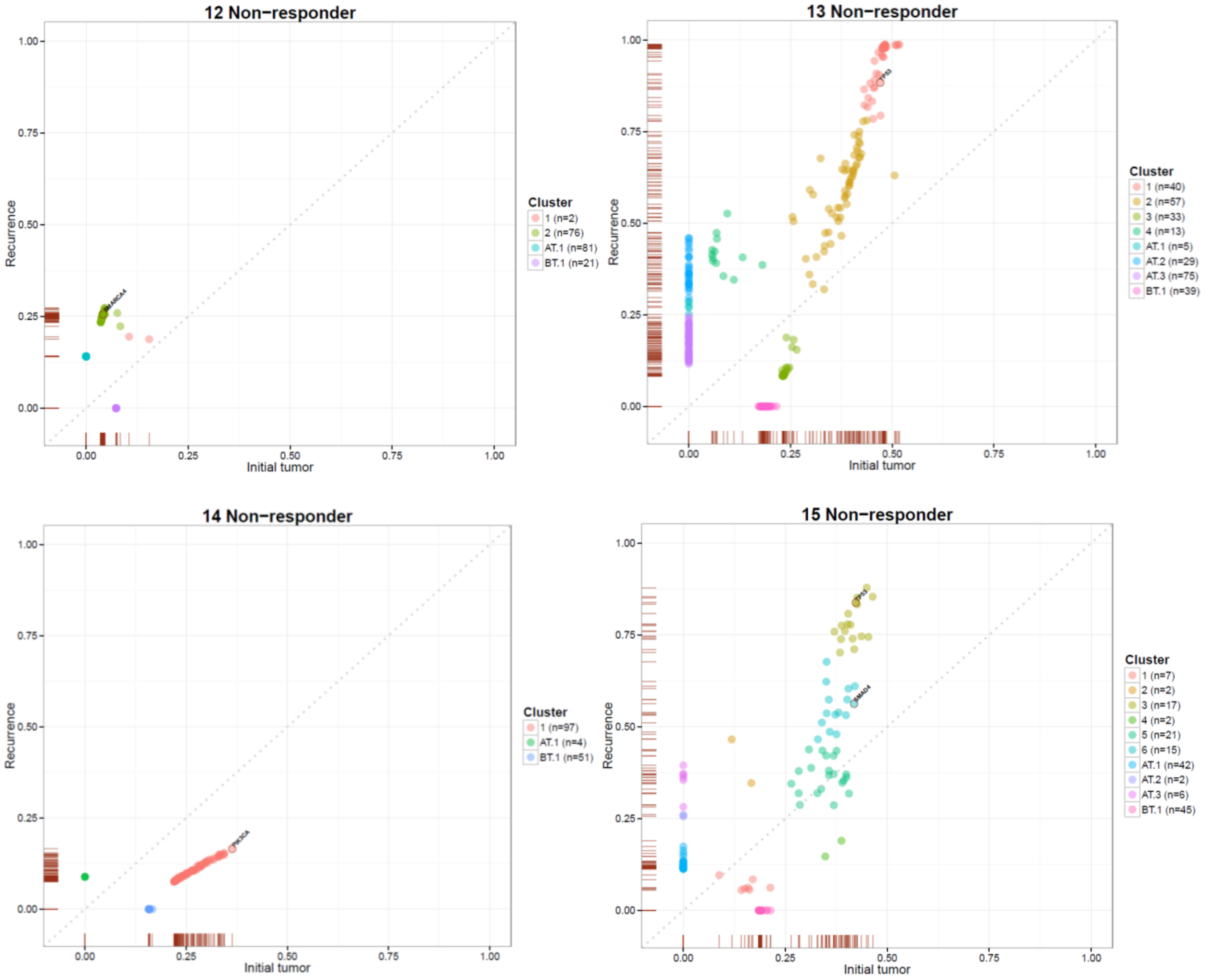
12 Non−responder
Recurrence
Initial tumor
Cluster
1 (n=2)
2 (n=76)
AT.1 (n=81)
BT.1 (n=21)
13 Non−responder
Recurrence
Initial tumor
Cluster
1 (n=40)
2 (n=57)
3 (n=33)
4 (n=13)
AT.1 (n=5)
AT.2 (n=29)
AT.3 (n=75)
BT.1 (n=39)
14 Non−responder
Recurrence
Initial tumor
Cluster
1 (n=97)
AT.1 (n=4)
BT.1 (n=51)
15 Non−responder
Recurrence
Initial tumor
Cluster
1 (n=7)
2 (n=2)
3 (n=17)
4 (n=2)
5 (n=21)
6 (n=15)
AT.1 (n=42)
AT.2 (n=2)
AT.3 (n=6)
BT.1 (n=45)

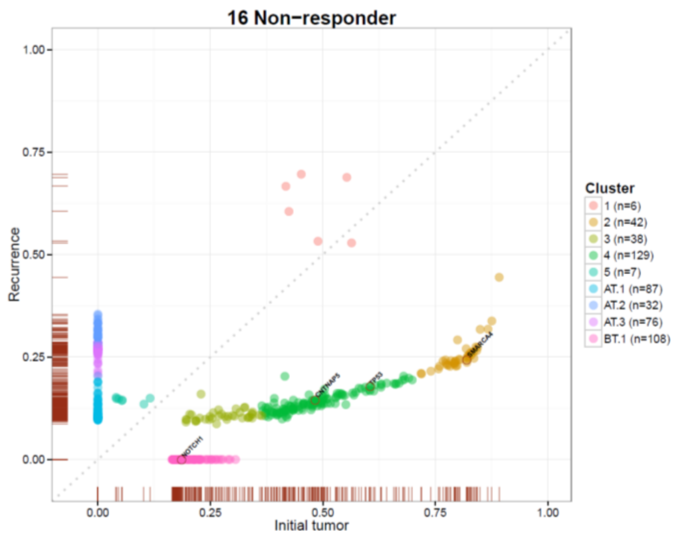
16 Non−responder
Recurrence
Initial tumor
0.00
0.25
0.50
0.75
1.00
Cluster
1 (n=6)
2 (n=42)
3 (n=38)
4 (n=129)
5 (n=7)
AT.1 (n=87)
AT.2 (n=32)
AT.3 (n=76)
BT.1 (n=108)

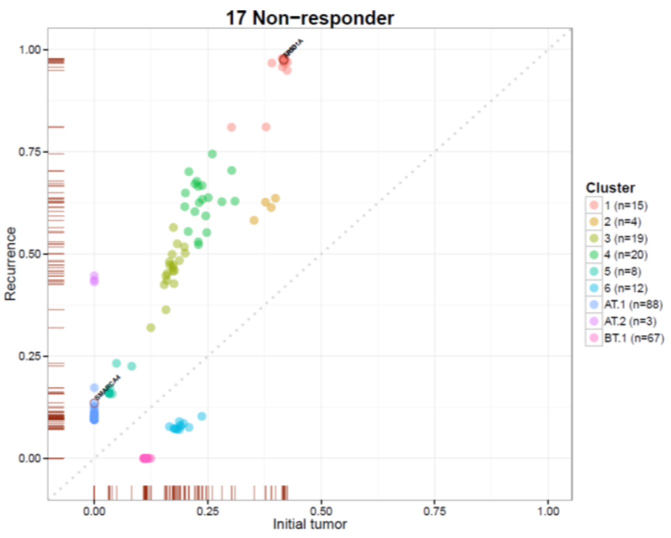
17 Non−responder
Recurrence
Initial tumor
0.00
0.25
0.50
0.75
1.00
Cluster
1 (n=15)
2 (n=4)
3 (n=19)
4 (n=20)
5 (n=8)
6 (n=12)
AT.1 (n=88)
AT.2 (n=3)
BT.1 (n=67)

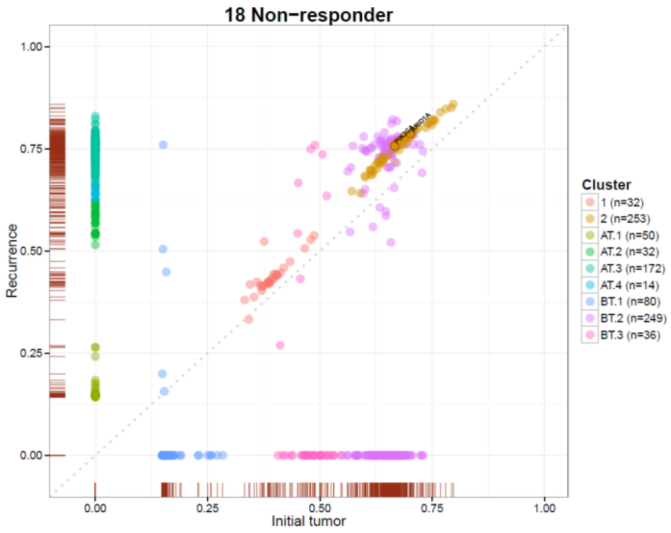
18 Non−responder
Recurrence
Initial tumor
0.00
0.25
0.50
0.75
1.00
Cluster
1 (n=32)
2 (n=253)
AT.1 (n=50)
AT.2 (n=32)
AT.3 (n=172)
AT.4 (n=14)
BT.1 (n=80)
BT.2 (n=249)
BT.3 (n=36)

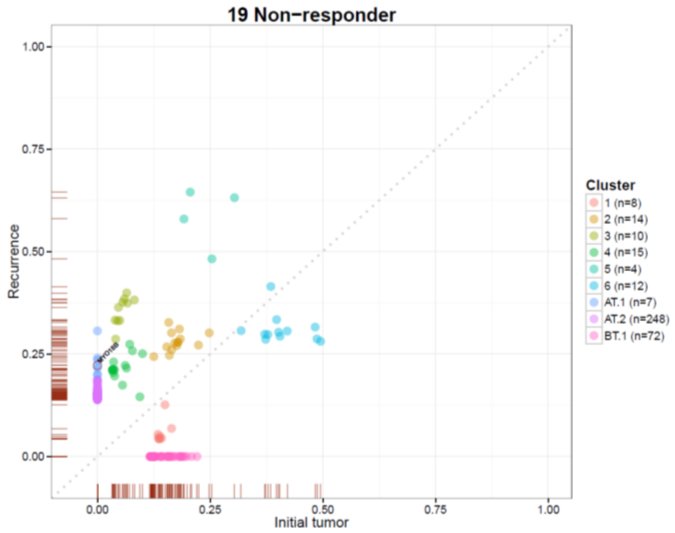
19 Non−responder
Recurrence
Initial tumor
0.00
0.25
0.50
0.75
1.00
Cluster
1 (n=8)
2 (n=14)
3 (n=10)
4 (n=15)
5 (n=4)
6 (n=12)
AT.1 (n=7)
AT.2 (n=248)
BT.1 (n=72)

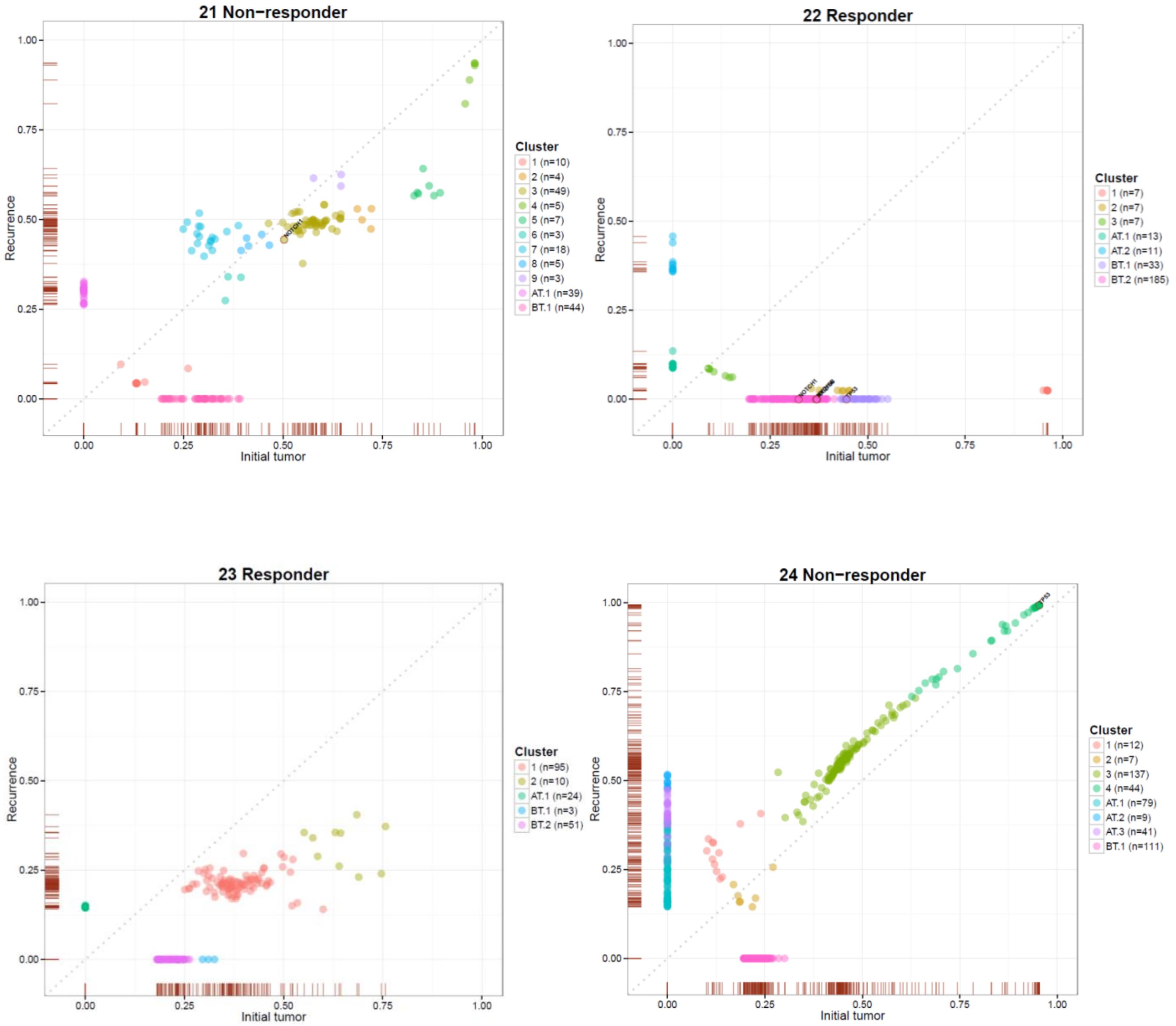
21 Non−responder
Recurrence
Initial tumor
Cluster
1 (n=10)
2 (n=4)
3 (n=49)
4 (n=5)
5 (n=7)
6 (n=3)
7 (n=18)
8 (n=5)
9 (n=3)
AT.1 (n=39)
BT.1 (n=44)
22 Responder
Recurrence
Initial tumor
Cluster
1 (n=7)
2 (n=7)
3 (n=7)
AT.1 (n=13)
AT.2 (n=11)
BT.1 (n=33)
BT.2 (n=185)
23 Responder
Recurrence
Initial tumor
Cluster
1 (n=95)
2 (n=10)
AT.1 (n=24)
BT.1 (n=3)
BT.2 (n=51)
24 Non−responder
Recurrence
Initial tumor
Cluster
1 (n=12)
2 (n=7)
3 (n=137)
4 (n=44)
AT.1 (n=79)
AT.2 (n=9)
AT.3 (n=41)
BT.1 (n=111)
0.00
0.25
0.50
0.75
1.00

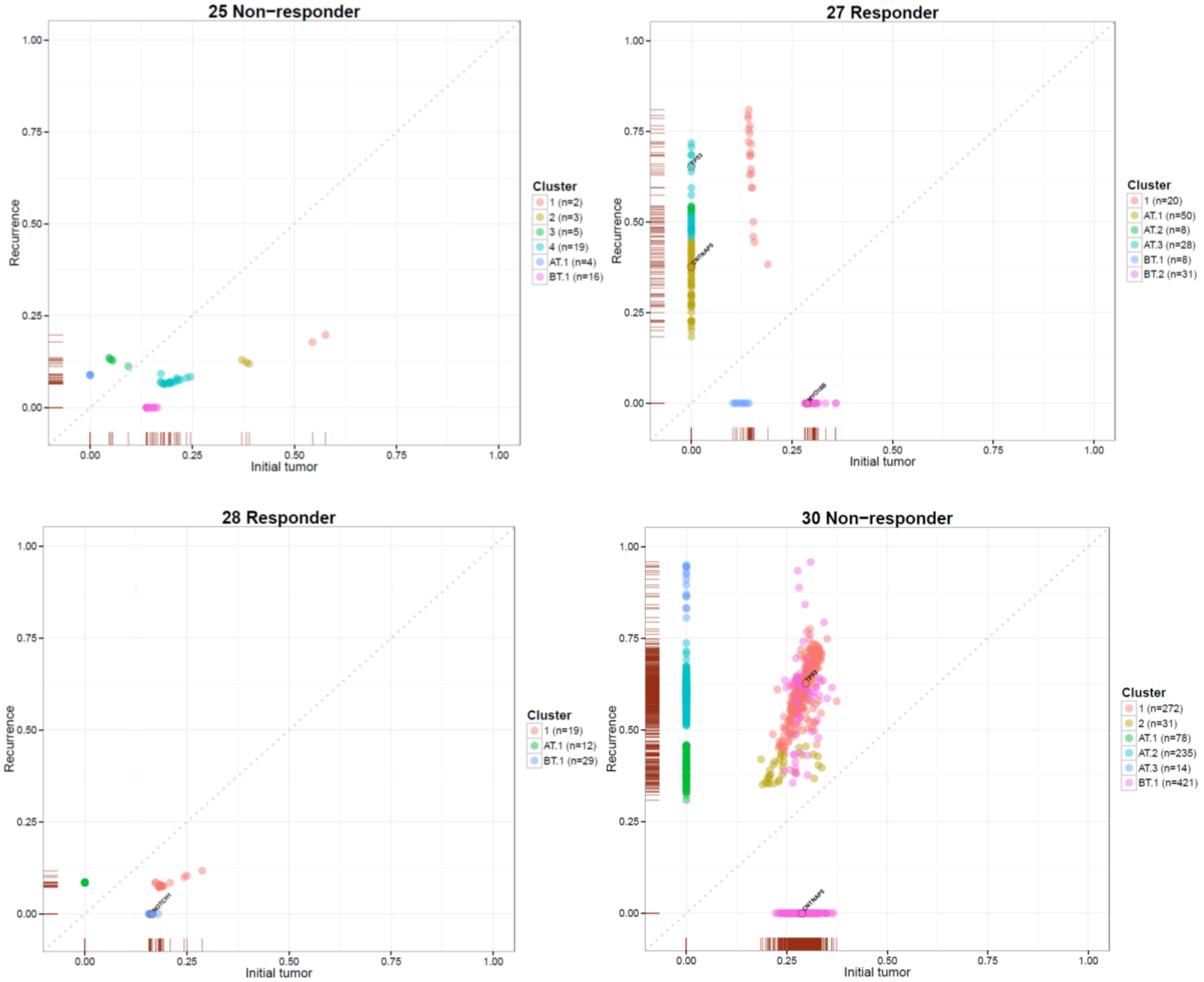

Supplementary Figure 8. Cancer evolution of cancers after treatment. X axis shows pre-treatment SNV VAF and Y axis, post-treatment VAF. Predicted clones are shown by colour. Given the excellent concordance with the deep Ampliseq data, we used exome sequencing data with a minimum read depth of 60X for this analysis [1]. Cancers excluded owing to purity concerns are not shown. Compared with bottleneckers, excluded cancers had lower numbers of mutations at lower VAFs in the post-treatment samples. We recognise, however, that bottlenecking cancers may be prone to decreased purity after treatment (Supplementary Note).

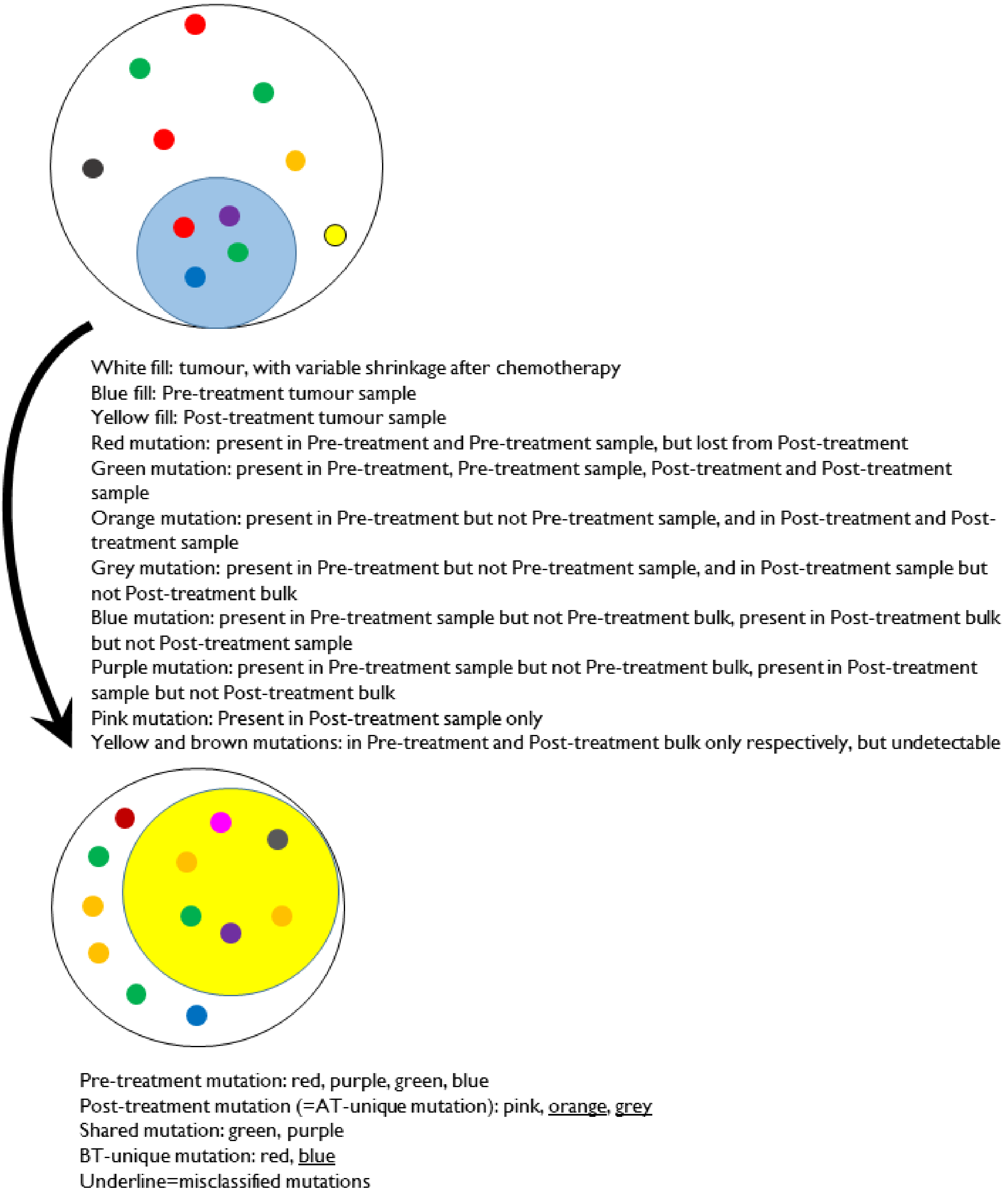

Supplementary Figure 9. Cancer sampling and mutation detection.
The Figure shows the tumour before and after treatment (white) with the sampled regions shaded blue and yellow respectively. Different types of mutation present in the data are coloured. Note that some of the mutations present in the original tumour are likely to have arisen in normal cells prior to the onset of tumorigenesis.

(a)

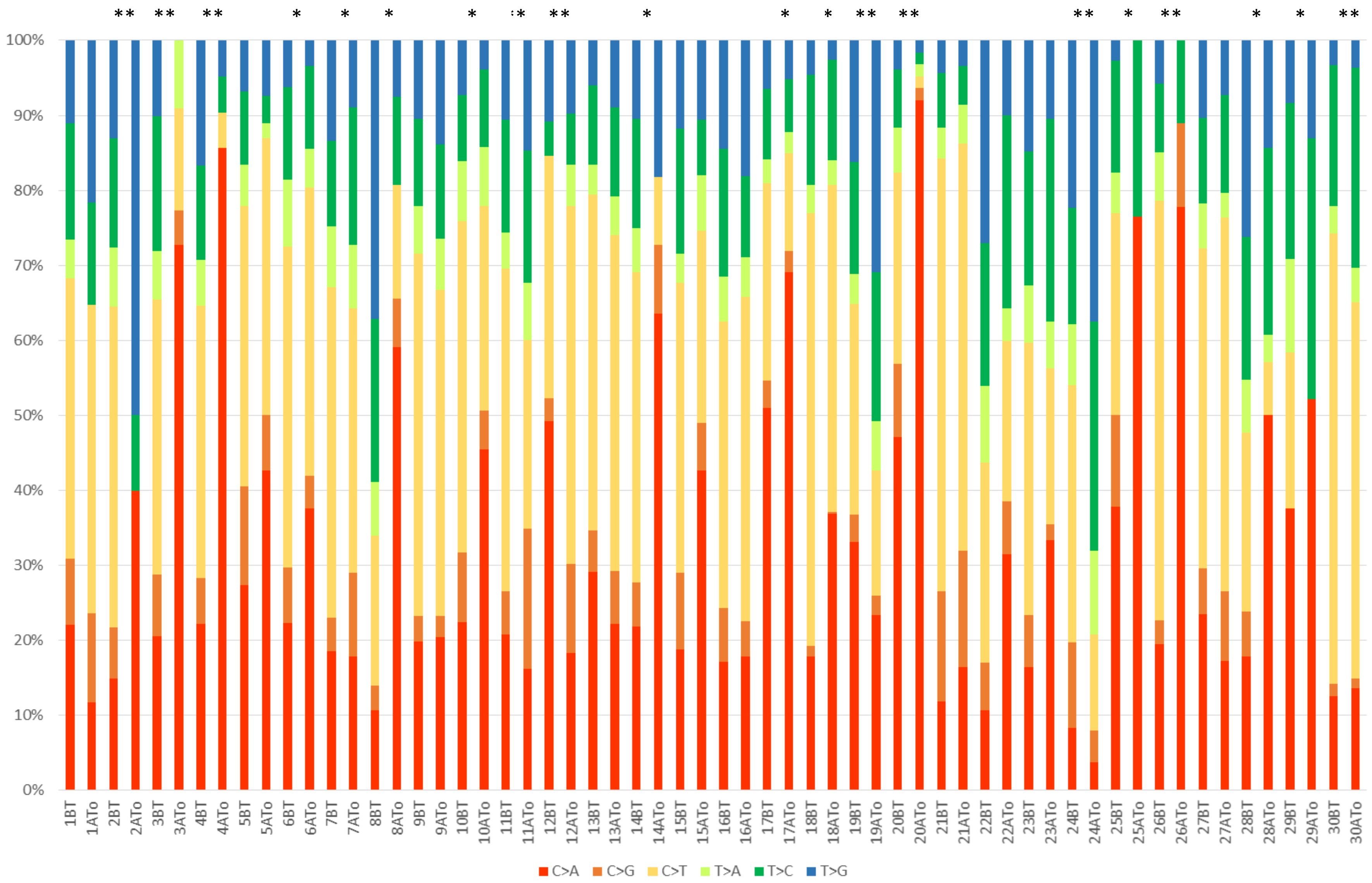

** ** ** * * * * :* ** * * * ** ** ** * ** * * **
100%
90%
80%
70%
60%
50%
40%
30%
20%
10%
0%
1BT 1ATo 2BT 2ATo 3BT 3ATo 4BT 4ATo 5BT 5ATo 6BT 6ATo 7BT 7ATo 8BT 8ATo 9BT 9ATo 10BT 10ATo 11BT 11ATo 12BT 12ATo 13BT 13ATo 14BT 14ATo 15BT 15ATo 16BT 16ATo 17BT 17ATo 18BT 18ATo 19BT 19ATo 20BT 20ATo 21BT 21ATo 22BT 22ATo 23BT 23ATo 24BT 24ATo 25BT 25ATo 26BT 26ATo 27BT 27ATo 28BT 28ATo 29BT 29ATo 30BT 30ATo
C>A C>G C>T T>A T>C T>G

(b)

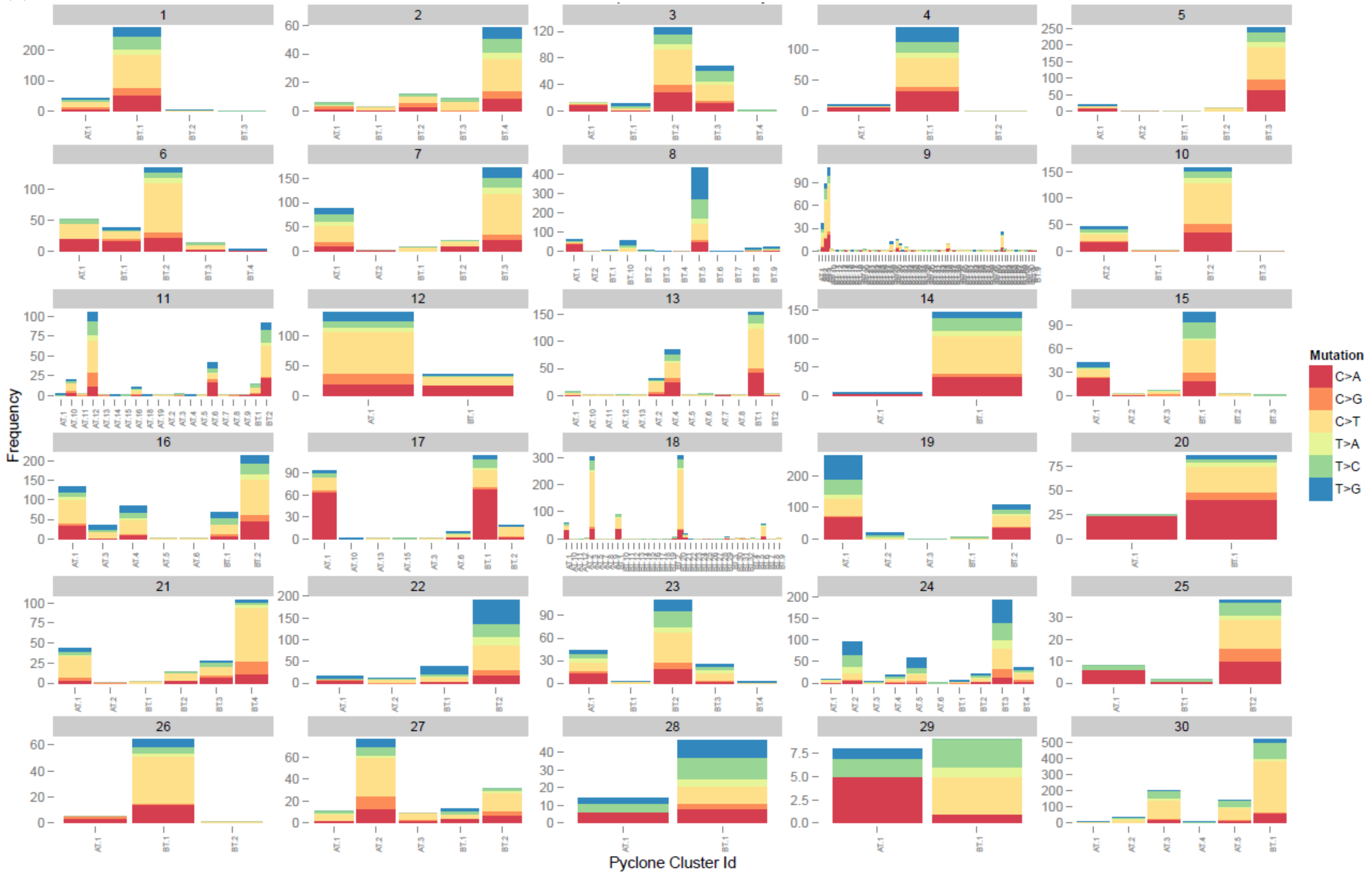

Supplementary Figure 10. Mutation spectra (a) in pre-treatment/shared and post-treatment samples, and (b) in sub-clones

(a) Pre-treatment/shared (BT) and post-treatment (ATo) mutations are shown. Test of VAF spectrum shift from mutation counts, ** P<0.001, * P<0.05 (b) Spectra are shown for selected, relatively populous sub-clones from each sample, with all pre-treatment (BT) and post-treatment (AT) sub-clone mutations displayed separately for each cancer. Shared clones between pre- and post-treatment samples are not shown.

(a)

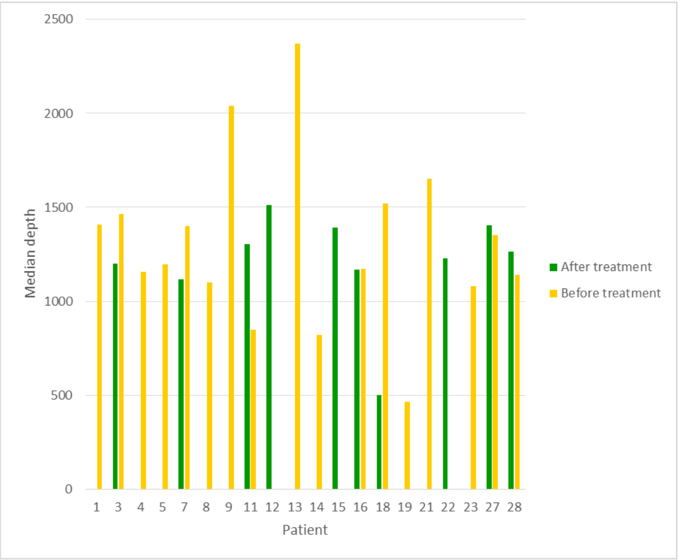

(b)

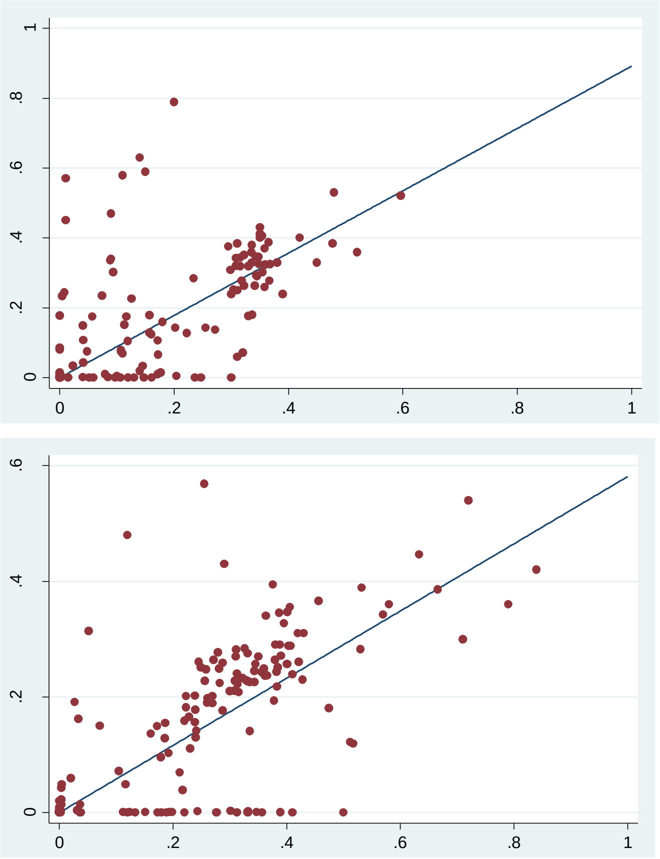

Supplementary Figure 11. Ampliseq FFPE sequencing

(a) Median depth of sequencing using the Ampliseq panel is shown for each FFPE sample. For several cancers, lack of FFPE sample precluded analysis.

(b) The plots show (upper) VAFs of SNVs present in the pre-treatment samples and (lower), VAFs of SNVs present in the post-treatment samples, with the Ampliseq fresh-frozen data on the x-axis and Ampliseq FFPE exome sequencing data on the y-axis. In both cases, there was good concordance (r^2=0.67, slope=0.89 and r^2=0.75, slope=0.58 respectively), although the reasons for the less steep slope in the post-treatment samples is unclear. Note that, owing to the higher possibility of false-positive calls in the FFPE samples, mutations outside the EAC drivers were not included in these plots if present in the FFPE samples only.

Case no.	Age	Sex	T	pT	N	Path R	Mandard	Bottleneck	1 year Rec	2 year Rec	5 year Rec	2 year OS	5 year OS
1	69	Male	2	3	0	1	3	0	0	1	1	0	0
2	78	Male	3	2	1	1	3		1	1	1	0	0
3	49	Male	3	3	1	1	3		0	1	1	0	0
4	53	Male	2	3	1	1	3		0	1	1	1	0
5	73	Male	3	3	0	1	2	1	0	0	1	1	0
6	63	Male	2	0	0	1	2	1	0	0	0	1	1
7	68	Male	3	2	1	1	3	1	0	0	0	1	1
8	54	Male	3	3	0	1	2	1	0	0	0	1	1
9	58	Female	2	1	0	1	3	1	0	0	0	1	1
10	61	Male	1	2	1	1	3	0	0	0	0	1	1
11	71	Male	3	2	1	0	4	1	1	1	1	0	0
12	56	Female	3	3	1	0	5	1	0	1	1	1	0
13	67	Male	2	3	1	0	4	0	0	1	1	0	0
14	62	Male	3	3	1	0	4	0	0	1	1	1	0
15	67	Male	2	3	1	0	4	0	1	1	1	0	0
16	60	Male	3	3	1	0	4	0	0	0	0	1	1
17	77	Male	3	2	1	0	5	0	0	0	1	1	0
18	68	Female	2	2	0	0	5	0	0	0	0	1	1
19	65	Male	2	0	3	0	4	0	0	0	0	1	1
20	65	Male	3	3	1	1	3		0	0	0	1	1
21	70	Male	3	3	1	0	4	0	0	0	1	1	0
22	60	Male	3	3	0	1	3	1	0	0	1	1	1
23	73	Male	3	2	0	1	3	0	0	0	0	1	1
24	57	Male	1	1	1	0	5	0	1	1	1	0	0
25	65	Female	3	4	1	0	4	0	1	1	1	0	0
26	76	Male	2	2	0	0	4		0	0	0	1	1
27	78	Female	4	2	0	1	3	1	0	0	0	1	1
28	74	Male	2	2	0	1	3	1	0	0	1	1	0
29	29	Male	3	4	0	0	5		0	1	1	0	0
30	69	Female	3	2	1	0	4	0	0	0	0	1	1

Supplementary Table 1. Clinical features of each of the 30 cases whose pre- and post-treatment samples underwent exome sequencing.

Surgical resection is the mainstay of radical treatment of oesophageal adenocarcinoma (EAC) in the USA and Europe. This is preceded by neo-adjuvant therapy (typically platinum-based chemotherapy with or without radiotherapy) in most cases[2] with the aim of reducing tumour burden. This confers an overall survival benefit of 7-13% at 2 years in locally advanced tumors [3]. Clinicopathological response to such therapy varies from complete, in a small proportion of cases, through partial to absent [4]. These data suggest that some tumours are intrinsically therapy-resistant whilst others are partially sensitive, or that the tumour contains a mixed population of sensitive and resistant cells (Supplementary Figure 1). However, since most cancers contain at least some resistant cells, *en bloc* resection and lymphadenectomy are performed for most patients. Despite this, overall prognosis following seemingly curative treatment remains poor, with just 30-40% survival at 5 years [5, 6]. The paired pre- and post-chemotherapy cancers undergoing exome sequencing in this study were derived from 15 patients who had a significant, but incomplete, pathological response (Mandard grade ≤3) to 2 cycles of 5FU and oxaliplatin neo-adjuvant therapy and 15 who showed very limited or no response (Mandard grade ≥4). The Table shows patient features by age, sex, stage (clinical and histological), binary pathological response, Mandard response grade, recurrence-free survival (Rec) years after enrolment into the study, and overall survival (OS).

Case	PathR	Chr	Position	Gene	Mutation
1	1	9	139,401,093	NOTCH1	c.3902-2A>G
1	1	17	7,577,548	TP53	p.Gly245Ser/c.733G>A
2	1	17	7,578,263	TP53	p.Arg196*/c.586C>T
3	1	17	7,577,565	TP53	p.Asn239Ser/c.716A>G
4	1	1	27,089,777	ARID1A	c.2732+1G>T
4	1	17	7,577,120	TP53	p.Arg273His/c.818G>A
5	1	3	178,936,082	PIK3CA	p.Glu542Lys/c.1624T>A
5	1	9	139,412,664	NOTCH1	p.Gly394Cys/c.1180G>T
5	1	17	7,577,082	TP53	p.Glu286Lys/c.856G>A
6	1	9	21,974,684	CDKN2A	p.Pro48Leu/c.143C>T
6	1	17	7,577,539	TP53	p.Arg248Trp/c.742C>T
6	1	19	11,134,251	SMARCA4	p.Arg973Trp/c.2917C>T
7	1	17	7,578,406	TP53	p.Arg175His/c.524G>A
8	1	1	27,105,825	ARID1A	p.Pro1813fs/c.5438delC
8	1	2	125,555,877	CNTNAP5	p.Val1065Ala/c.3194T>C
8	1	2	125,623,000	CNTNAP5	p.Glu1111Gly/c.3332A>G
8	1	17	7,577,114	TP53	p.Cys275Tyr/c.824G>A
8	1	18	48,591,918	SMAD4	p.Arg361Cys/c.1081C>T
9	1	17	7,578,406	TP53	p.Arg175His/c.524G>A
9	1	19	11,132,519	SMARCA4	p.Thr912Ile/c.2735C>T
10	1	17	7,578,406	TP53	p.Arg175His/c.524G>A
11	0	4	153,249,385	FBXW7	p.Arg465Cys/c.1393C>T
11	0	5	9,063,023	SEMA5A	p.Glu832*/c.2494G>T
11	0	9	139,400,204	NOTCH1	p.Pro1381fs/c.4143delC
11	0	9	139,412,252	NOTCH1	p.Ala465Thr/c.1393G>A
11	0	17	7,577,018	TP53	c.919+1G>T
11	0	17	7,577,120	TP53	p.Arg273His/c.818G>A
12	0	19	11,144,113	SMARCA4	p.Gly1232Ser/c.3694G>A
13	0	1	27,100,207	ARID1A	p.Arg1335*/c.4003C>T
13	0	17	7,578,526	TP53	p.Cys135Phe/c.404G>T
14	0	3	178,916,957	PIK3CA	p.Arg115Gln/c.344G>A
14	0	17	7,577,062-75	TP53	p.Asn288ArgFS/c.863-876del14
15	0	17	7,577,539	TP53	p.Arg248Trp/c.742C>T
15	0	18	48,604,673	SMAD4	p.Cys499Arg/c.1495T>C
16	0	2	125,555,822	CNTNAP5	p.Thr1047Ser/c.3139A>T
16	0	9	139,412,282	NOTCH1	p.Glu455Gln/c.1363G>C
16	0	17	7,577,539	TP53	p.Arg248Gly/c.742C>G
17	0	1	27,105,892	ARID1A	p.Gln1835*/c.5503C>T
17	0	17	7,577,121	TP53	p.Arg273Cys/c.817C>T
18	0	1	27,089,541	ARID1A	p.Asn833His/c.2497A>C
18	0	1	27,105,930	ARID1A	p.Asp1850fs/c.5548delG
18	0	3	41,266,100	CTNNB1	p.Ser33Pro/c.97T>C

18	0	3	178,916,936	PIK3CA	p.Arg108His/c.323G>A
18	0	4	153,244,155	FBXW7	p.Gly667fs/c.2001delG
18	0	9	21,970,970	CDKN2A	p.Leu130Met/c.388C>A
18	0	17	7,578,457	TP53	p.Arg158His/c.473G>A
18	0	17	7,579,382	TP53	p.Thr102fs/c.304delA
18	0	17	7,579,470	TP53	p.Pro72fs/c.216delC
18	0	X	123,182,927	STAG2	p.Arg298Cys/c.892C>T
19	0	17	7,577,121	TP53	p.Arg273Cys/c.817C>T
20	1	9	139,401,780	NOTCH1	p.Cys1207Phe/c.3620G>T
20	1	13	66,878,846	PCDH9	p.Leu1185Met/c.3553C>A
20	1	17	7,578,413	TP53	p.Val173Leu/c.517G>T
21	0	9	139,396,305	NOTCH1	p.Gln1845Glu/c.5533C>G
21	0	X	123,181,311	STAG2	p.Arg259*/c.775C>T
22	1	1	27,101,273	ARID1A	p.Gln1519*/c.4555C>T
22	1	9	139,409,067	NOTCH1	p.Thr701Ile/c.2102C>T
22	1	17	7,577,022	TP53	p.Arg306*/c.916C>T
24	0	17	7,578,478	TP53	p.Pro151Arg/c.452C>G
27	1	22	26,228,912	MYO18B	p.Pro1003Gln/c.3008C>A
28	1	9	139,412,239	NOTCH1	p.Asp469Gly/c.1406A>G
28	1	17	7,577,551	TP53	p.Gly244Ser/c.730C>A
29	0	17	7,577,094	TP53	p.Arg282Trp/c.844C>T
30	0	1	27,099,102	ARID1A	p.Pro1175fs/c.3524delC
30	0	2	125,671,851	CNTNAP5	p.Glu1303Lys/c.3907G>A
30	0	12	25,398,281	KRAS	p.Gly13Asp/c.38G>A
30	0	13	67,800,839	PCDH9	p.Gln578His/c.1734A>T
30	0	17	7,577,139	TP53	p.Arg267Trp/c.799C>T
30	0	17	7,578,274-6	TP53	p.Pro191del/c.572_574delCTC

Supplementary Table 2. Somatic EAC driver mutations in the pre-treatment samples discovered in exome sequencing data.

Case=patient number; PathR=binary pathological response; Chr=chromosome; Position=Human Genome reference location (Build 37); Gene=driver gene; Mutation=protein level change/DNA level change. Mutations in the pre-treatment sample found below a VAF cut-off of 0.02, but present at higher VAF in post-treatment samples are not shown here, but are listed in Supplementary Table 3. Supplementary Tables 4 and 8 show driver mutations discovered in Ampliseq data, most of which were subsequently found to be present in the exome data.

(a)

Case	Gene	Mutation	VAF Pre- > Post-
1	TP53	G245S	0.04 > 0.13
1	TP53	Y220C	0.06 > 0.00
1	TP53	H193P	0.05 > 0.00
2	TP53	R196X	0.37 > 0.00
3	TP53	N239S	0.29 > 0.00
4	TP53	R273H	0.13 > 0.01
5	TP53	E286K	0.14 > 0.00
6	TP53	R248W	0.50 > 0.00
7	TP53	R175H	0.55 > 0.14
8	TP53	C275Y	0.33 > 0.00
9	TP53	R175H	0.71 > 0.02
10	TP53	R175H	0.12 > 0.14
11	TP53	c.919+1G>T	0.15 > 0.00
11	TP53	R273H	0.08 > 0.74
12	TP53	R158H	0.02 > 0.11
13	TP53	C135F	0.32 > 0.43
14	TP53	N288fs	0.33 > 0.23
15	TP53	R248W	0.21 > 0.52
16	TP53	R248G	0.31 > 0.11
17	TP53	R273C	0.22 > 0.55
18	TP53	R158H	0.05 > 0.00
18	TP53	T102fs	0.03 > 0.38
18	TP53	P72fs	0.31 > 0.44
19	TP53	R273C	0.28 > 0.27
20	TP53	V173L	0.07 > 0.00
22	TP53	R306X	0.45 > 0.00
23	TP53	c.375+2G>C	0.40 > 0.21
24	TP53	P151R	0.71 > 0.82
27	TP53	Y220C	0.00 > 0.67
28	TP53	G244S	0.02 > 0.04
29	TP53	R282W	0.50 > 0.46 (?germline)
30	TP53	R267W	0.16 > 0.31
30	TP53	P191del	0.05 > 0.16
30	TP53	R273C	0.13 > 0.01

(b)

Case	Gene	Mutation	VAF Pre-	COSMIC	cBio	SIFT	PP2
5	STAG2	G1080R	0.00 > 0.71	No	No	0.54	0.00
18	ENSP00000218089	R298C	0.14 > 0.70	No	No	0.02	1.00
21		R259X	0.56 > 0.39				
3	ARID1A	P289L	0.00 > 0.09	No	No	0.43	0.32
4	ENSP00000320485	c.2732+1G>T	0.13 > 0.00				
8		P1813fs	0.41 > 0.00				
13		R1335X	0.24 > 0.72				
17		E1835X	0.20 > 0.48				
18		N833H	0.40 > 0.51	No	No	0.05	1.00
18		D1850fs	0.24 > 0.34				
22		R1519X	0.30 > 0.00				
30		P1175fs	0.10 > 0.26				
8	SMAD4	R361C	0.18 > 0.00	Many	Many	0.00	1.00
15	ENSP00000381452	C499R	0.27 > 0.27	Yes (1) + 3 at site	2 at site	0.00	1.00
16		R361C	0.15 > 0.00			0.00	1.00
11	FBXW7	R465C	0.05 > 0.84	Many	Many	0.00	1.00
17	ENSP00000281708	R505S	0.00 > 0.11	Many	Many	0.00	1.00
18		G667fs	0.36 > 0.03				
1	NOTCH1	c.3902-2A>G	0.21 > 0.00				
5	ENSP00000277541	G394C	0.10 > 0.00	No	No	0.02	1.00

11		P1381fs	0.26 > 0.00				
11		A465T	0.13 > 0.00	Yes (5)	Yes (3) + 2 at site	0.00	0.99
16		E455Q	0.10 > 0.00	No	1 at site	0.03	1.00
20		T701I	0.19 > 0.00	No	2 at site	0.20	0.93
21		C1207F	0.08 > 0.00	No	No	0.00	1.00
22		Q1845E	0.23 > 0.16	No	No	1.00	0.13
28		D469G	0.23 > 0.00	3 at site	2 at site	0.02	1.00
28		E610X	0.16 > 0.00				
5	PIK3CA	E542K	0.13 > 0.00	Many	Many	0.00	1.00
14	ENSP00000263967	R115Q	0.29 > 0.10	5 at site	1 at site	0.00	0.14
17		R115L	0.00 > 0.10	Yes (4) + 1 at site	Yes (4)	0.13	0.79
18		R108H	0.30 > 0.35	Many	Many	0.00	1.00
22		E542K	0.22 > 0.00	Many	Many	0.00	1.00
25		E542K	0.13 > 0.00	Many	Many	0.00	1.00
30		L99P	0.03 > 0.00	Many	Many	0.02	1.00
19	MYO18B	L901R	0.00 > 0.23	No	No	0.07	0.95
27	ENSP00000334563	P1003E	0.17 > 0.00	No	1 at site	0.27	1.00
6	CDKN2A	P48L	0.24 > 0.52	Several	Several	0.00	1.00
13	ENSP00000462950	c.206+1G>C	0.22 > 0.64				
18		L130M	0.62 > 0.53	11 at site	1 at site	0.00	1.00
8	CNTNAP5	V1065A	0.15 > 0.00	No	Yes (1)	1.00	0.00
8	ENSP00000399013	E1111G	0.15 > 0.00	No	No	0.98	0.01
16		T1047S	0.23 > 0.10	No	No	0.33	0.01
27		V1045L	0.00 > 0.24	No	No	1.00	0.00
30		E1303K	0.17 > 0.00	No	No	0.00	1.00
6	SMARCA4	R973W	0.19 > 0.30	Yes (1) + 4 at site	Several	0.00	1.00
9	ENSP00000395654	T912I	0.79 > 0.00	1 at site	1 at site	0.00	1.00
12	ENSP00000343896	G1232S	0.04 > 0.15	Several	Several	0.00	0.99
16	ENSP00000445036	R1135W	0.00 > 0.40	Yes (1) + 2 at site	2 at site	0.00	1.00
17	ENSP00000392837	V1016M	0.00 > 0.12	Yes (2)	Yes (2)	0.00	1.00
20	PCDH9	L1185M	0.06 > 0.00	No	No	0.19	0.00
30	ENSP00000367096	Q578H	0.22 > 0.48	No	No	0.02	0.97
11	SEMA5A	E832X	0.05 > 0.17	No	No		
	ENSP00000367096						
30	KRAS	G13D	0.31 > 0.26	Many	Many	0.00	1.00
	ENST00000256078						
30	CTNNB1	S33P	0.34 > 0.32	Many	Many	0.00	1.00
30	ENSP00000392837	A525V	0.33 > 0.37	No	No	0.24	0.40

Supplementary Table 3. Driver gene mutation VAF change and functionality in sequencing data.
Exome sequencing data are shown, except for changes only detected by Ampliseq (Supplementary Tables 4 and 8). Cancers subsequently excluded from the clonal evolution analysis owing to purity concerns in post-treatment samples are shown for completeness, in italics. VAF changes are not corrected for tumour purity or ploidy.
(a) p53 mutations. These all had strong predicted pathogenic effects. The VAF changes are shown graphically in Supplementary Figure 7.
(b) Mutations in driver genes apart from p53. For missense changes, mutations at the same site (including different amino acid changes) are shown from the overlapping, but non-identical, databases, COSMIC (http:// http://cancer.sanger.ac.uk/cancergenome/projects/cosmic/) and cBioPortal (http://www.cbioportal.org/), as at September 2015: "Many" or "Several" = >5 or >10 examples of the specific mutation respectively; "Yes (N)"=<5 examples of the specific mutation (number); and "N at site"=number of other SNVs involving that amino acid. In addition, SIFT (low=functional) and Polyphen2 (high=functional) prediction scores are shown. Protein-truncating and splice site mutations are regarded as likely to be functional. Ensembl protein reference links are also provided.

Case	PathR	Chr	Position	Gene	Mutation	Change after treatment	Notes
30	0	3	178,916,910	PIK3CA	p.Leu99Phe/c.297A>T	Retained, low VAF	1
22	1	3	178,936,082	PIK3CA	p.Glu542Lys/c.1624T>A	Lost	2
13	0	9	21,970,901	CDKN2A	p.Gly102Arg/c.304A>C	Retained	3
28	1	9	139,410,010	NOTCH1	p.Gln610X/c.1829G>A	Lost	2
28	1	9	139,412,239	NOTCH1	p.Asp469Gly/c.1406A>G	Retained, low VAF	2
30	0	17	17,577,121	TP53	p.Arg273Cys/c.817C>T	Lost	2
1	1	17	17,578,271	TP53	p.His193Pro/c.578A>C	Lost	1
12	0	17	17,578,457	TP53	p.Arg158His/c.473G>A	Retained	2
23	1	17	17,579,310	TP53	c.375+2G>C	Retained	3
16	0	18	48,591,919	SMAD4	p.Arg361His/c.1082G>A	Gained	1

Supplementary Table 4. Mutations found by Ampliseq deep sequencing of pre-treatment fresh-frozen samples, but not called in the exome sequencing data.

All of these changes either were predicted by SIFT and Polyphen2 to have deleterious consequences or were present recurrently in COSMIC and/or cBioPortal. The reasons for the failure of variants to be called in the exome sequencing data are shown by: 1. VAF below threshold in exome data; 2. Present in exome sequencing, but not called because surrounding region sub-optimal quality; 3. Low coverage in exome sequencing.

Case	Chr	Start Position	End Position	Gain/Deletion	Gene
3BT	1	26,411,918	28,251,700	Deletion	ARID1A
3BT	5	38,139	1,377,172	Gain	TERT
3BT	19	38,872,536	40,404,343	Gain	AKT2
4AT	17	37,771,746	38,948,438	Gain	ERBB2
5BT	8	128,355,019	129,128,194	Gain	MYC
10AT	12	4,066,795	4,823,986	Gain	CCND2
14AT	18	18,561,020	19,867,362	Gain	GATA6
17AT	18	48,251,054	49,804,768	Deletion	SMAD4
18BT	5	38,139	1,317,949	Gain	TERT
19BT	12	24,117,433	25,861,867	Gain	KRAS
22AT	1	44,844,958	46,743,900	Deletion	MUTYH
23BTAT	17	37,868,853	40,491,803	Gain	ERBB2
24BTAT	11	408,352	733,639	Gain	HRAS
24BTAT	17	37,711,565	38,813,824	Gain	ERBB2
24AT	19	10,897,613	11,374,675	Deletion	SMARCA4
24BTAT	19	29,971,226	30,908,586	Gain	CCNE1
24BTAT	20	14,615,924	15,455,573	Deletion	MACROD2
27BT	9	21,573,016	22,291,931	Deletion	CDKN2A
27AT	5	38,139	1,493,608	Gain	TERT
27AT	19	28,959,499	30,105,969	Gain	CCNE1

Supplementary Table 5. Focal copy number changes involving known oncogenes, tumour suppressors and other reported sites of such changes in gastrointestinal cancer in the 23 cancer pairs analysed by SNP arrays [7]. Chromothripsis (>10 segmental copy number changes per chromosome) was found in 15 samples (8 pre-treatment, 7 post-treatment) and was not associated with treatment response. Gains and deletions were also found in multiple samples at the *FHIT* fragile site and are not shown in the table.

Gene	Functional mutations			VAF (median, IQR)	
	BT-only	Shared	AT-only	Before	After
ERBB2 (Lapatinib)	0	1 (2)	0 (1)	0.059 (NA)	0.102 (NA)
KIT (Sunitinib/Sorafenib/Imatinib)	0	1	0	0.122 (NA)	0.104 (NA)
ABL2 (Imatinib/Dasatinib/GNF-2)	0	1	0	0.118 (NA)	0.333 (NA)
BRAF (AZ628)	0	0	1	0.000 (NA)	0.186 (NA)
ALK (Crizotinib/NVP-TAE684)	0	3	0	0.191 (0.096-0.254)	0.024 (0.015-0.046)
MET (Crizotonib)	0	1	0	0.251 (NA)	0.024 (NA)
CDK4 (CGP-082996/Seliciclib)	0	1	0	0.688 (NA)	0.024 (NA)
CDK5 (CGP-60474/Seliciclib)	0	1	0	0.313 (NA)	0.412 (NA)
PLK1 (BI-2536/GW843682X)	0	1	0	0.311 (NA)	0.184 (NA)
RPS6KA2 (CMK)	0	2	0	0.320 (0.229-0.410)	0.012 (0.007-0.018)
AKT1 (A-443654)	0	1	0	0.109 (NA)	0.011 (NA)
AKT2 (A-443654)	(1)	0	0		
CCNE1 (CYC065)	0	(1)	(1)		
CCND2 (PD-0332991)	0	0	(1)		
PRKAG2 (A-769662)	0	1	0	0.174 (NA)	0.485 (NA)
RET (Lestaurtinib)	0	1	0	0.098 (NA)	0.011 (NA)
WEE1 (681640)	0	0	1	0.000 (NA)	0.400 (NA)
FGFR2 (PD-173074)	0	1	0	0.104 (NA)	0.003 (NA)

Supplementary Table 6. Potentially-actionable mutations
We searched the following genes (targeted therapies in brackets) for mutations in our EACs: EGFR (Erlotinib/Lapatinib), ERBB2 (Lapatinib), MTOR (Rapamycin/Torin 1), PDGFRA (Sunitinib/Sorafenib), PDGFRB (Sunitinib/Sorafenib), VEGFR (Sunitinib/Sorafenib), VEGFA (Bevacuzimab), FLT3 (Sunitinib/Sorafenib), KIT (Sunitinib/Sorafenib/Imatinib), ABL1 (Imatinib/Dasatinib/GNF-2), ABL2 (Imatinib/Dasatinib/GNF-2), BRAF (AZ628), AURKA (VX680), AURKB (VX680), AURKC (VX680), ALK (Crizotinib/NVP-TAE684), MET (Crizotonib), SRC (Saracatanib/Dasatanib), KIF11 (NSC83265), APH1A (Z-LLNle-CHO), APH1B (Z-LLNle-CHO), BCR (GNF-2), CDK1 (CGP-60474/Seliciclib), CDK2 (CGP-60474/Seliciclib), CDK4 (CGP-082996/Seliciclib), CDK5 (CGP-60474/Seliciclib), CDK7 (CGP-60474/Seliciclib), CDK9 (CGP-60474/Seliciclib), BMX (WZ-1-84), TEC (WZ-1-84), PLK1 (BI-2536/GW843682X), PLK2 (BI-2536), PLK3 (BI-2536), IGF1R (BMS-536924), ITK (BMS-509744), RPS6KA2 (CMK), DHFR (Daraprim), AKT1 (A-443654), AKT2 (A-443654), AKT3 (A-443654), NFKB (Parthenolide), IKKE (KIN001-135), ERK5 (XMD8-85), PPP1R15A (Salubrinal), ROCK1 (GSK269962A), ROCK2 (GSK269962A), PRKAA1 (A-769662), PRKAA2 (A-769662), PRKAB1 (A-769662), PRKAB2 (A-769662), PRKAG1 (A-769662), PRKAG2 (A-769662), PRKAG3 (A-769662), SHP1 (NSC-87877), SHP2 (NSC-87877), GSK3B (CHIR-99021), PI3KB (AZD6482), JNK (JNK-9L), FAK (PF-562271), P4HA1 (DMOG), P4HA3 (DMOG), FNTA (FTI-277), XIAP (Embelin), CASP3 (PAC-I), PAK (IPA-3), SGK3 (GSK-650394), SYK (BAY 61-3606), BCL2 (Obatoclax Mesylate), BCLXL (Obatoclax Mesylate), MCL2 (Obatoclax Mesylate), BTK (LFM-A13), HSP90 (AUY922), PRKC (Bryostatin 1), MEK1 (RDEA119), MEK2 (RDEA119), PARP1 (AZD-2281), PARP2 (AZD-2281), TNFA (Lenalidomide), CHK1 (AZD7762), CHK2 (AZD7762), NTRK1 (GW 441756), JAK (Lestaurtinib), RET (Lestaurtinib), MAPK11 (VX-702), MAPK12 (VX-702), MAPK13 (VX-702), MAPK14 (VX-702), ATM (KU-55933), HSP70 (Elesclomol), SMO (Vismodegib), TBK1 (BX-795), PDK1 (BX-795), IKK (BX-795), DNAPK (NU-7441), PIM3 (SL 0101-1), WEE1 (681640), MDM2 (Nutlin-3a), CDK2-CCNE1 (CYC065), CDK6-CCND2 (PD-0332991), FGFR1 (PD-173074), FGFR2 (PD-173074) and FGFR3 (PD-173074). All somatic SNVs of each gene are shown in the table below, with mutations previously found in cancer shown in bold. Figures in brackets are numbers of tumours with focal copy number changes.

Mutation type	Responder v Non-responder		Bottlenecker v Non-bottlenecker	
	Median, count or proportion	P	Median, count or proportion	P
SNV+indel number	432 v 314	0.093	575 v 365	**0.043**
SCNA burden	14 v 9	0.577	10 v 10	0.383
EAC driver burden	2 v 2	0.740	3 v 2	0.148
MSI	0 v 2	0.483	0 v 2	1.000
p53 mutation	14 v 11	0.330	5 v 15	0.544
C>A proportion	0.211 v 0.258	0.561	0.221 v 0.229	0.977
C>G proportion	0.071 v 0.060	0.165	0.059 v 0.073	0.349
C>T proportion	0.364 v 0.404	0.395	0.361 v 0.409	0.429
T>A proportion	0.071 v 0.050	**0.0028**	0.064 v 0.052	0.061
T>C proportion	0.141 v 0.136	0.787	0.136 v 0.140	0.930
T>G proportion	0.143 v 0.092	0.089	0.159 v 0.097	0.169

Supplementary Table 7. Summary of associations between molecular variables in pre-treatment sample and clinical response to therapy.
Global mutation measures derived from pre-treatment samples were assessed for associations with response (N=30, or N=23 for SCNAs) as a binary variable. Associations with bottlenecking (N=24) were also assessed. P values were derived from the t test for continuous variables and Fisher's exact test for categorical variables. Medians, counts or proportions in responder v non-responders and in bottleneckers v non-bottleneckers are shown in the three sub-panels as appropriate for the explanatory variable in question. For mutation and SCNA burden tests, the MSI+ cancers were excluded. Relapse-free survival was also tested (not shown) for associations as a binary variable in reverse stepwise logistic regression model. For two-year survival, significant independent associations were found for age (OR=0.861, P=0.045), pathological response (OR=0.0571, P=0.035) and SNV+indel burden in the post-treatment sample (OR=0.991, P=0.040). For five-year survival, only associations with pathological response (OR=0.0366, P=0.035) and SNV+indel burden in the post-treatment sample (OR=0.994, P=0.040) remained. These associations were not present for overall survival in a CoxPH model

Case	Gene	Mutation	Frozen	FFPE	Notes
3	TP53	p.Asn239Ser/c.716A>G	Loss	Loss	Concordant
7	TP53	p.Arg175His/c.524G>A	Retention	Retention	Concordant
11	FBXW7	p.Arg465Cys/c.1393C>T	Retention	Retention	Concordant
11	NOTCH1	p.Pro1381fs/c.4143delC	Loss	Absence	NOTCH1 sub-clonal/regional
11	NOTCH1	p.Ala465Thr/c.1393G>A	Loss	Absence	NOTCH1 sub-clonal/regional
11	TP53	c.919+1G>T	Loss	Absence	p53 sub-clonal/regional
11	TP53	p.Arg273His/c.818G>A	Retention	Retention	Concordant
16	TP53	p.Arg248Gly/c.742C>G	Retention	Retention	Concordant
16	SMARCA4	p.Arg1135Trp/c.3403C>T	Retention	Retention	Concordant
18	ARID1A	p.Asn833His/c.2497A>C	Retention	Loss	Loss, unlike original
18	ARID1A	p.Asp1850fs/c.5548delG	Retention	Retention	Concordant
18	CTNNB1	p.Ser33Pro/c.97T>C	Retention	Retention	Concordant
18	PIK3CA	p.Arg108His/c.323G>A	Retention	Retention	Concordant
18	FBXW7	p.Gly667fs/c.2001delG	Retention	Retention	Concordant
18	CDKN2A	p.Leu130Met/c.388C>A	Retention	Retention	Concordant
18	TP53	p.Arg158His/c.473G>A	Loss	Loss	Concordant
18	TP53	p.Thr102fs/c.304delA	Retention	Retention	Concordant
18	TP53	p.Pro72fs/c.216delC	Retention	Retention	Concordant
27	TP53	p.Tyr220Cys/c.659A>G	Gain	Retention	No gain, unlike original
28	NOTCH1	p.Asp469Gly/c.1406A>G	Loss	Absence	NOTCH1 sub-clonal/regional
28	TP53	p.Gly244Ser/c.730C>A	Retention	Retention	Concordant

Supplementary Table 8. Ampliseq deep sequencing of FFPE samples

The table compares loss, gain and retention of driver gene mutations between paired pre- and post-treatment in frozen and FFPE samples for the cancers in which both pre- and post-treatment samples were available. Frozen sample data were derived from whichever of the exome and Ampliseq experiments had higher coverage. Alleles were scored as retained based on VAF calling thresholds used for each platform (see Methods). Mutations not present in the FFPE data are shown as absent. In addition three mutations were found by Ampliseq deep sequencing of FFPE samples, but were not present in the fresh-frozen sample sequencing data. These were: case #3, *ARID1A* p.Pro289Leu/c.866C>T in post-treatment sample; case #13 *NOTCH1* p.Leu590Phe/c.1768A>T in pre-treatment sample (post-treatment sample not done); and case #30 *NOTCH1* p.Pro2512Thr/c.7534C>A in post-treatment sample (pre-treatment sample not done). The last of these was predicted to have deleterious consequences and was present recurrently in COSMIC and cBioPortal. It should be borne in mind that occasional artefactual C:G>T:A mutations owing to 5'methyl-cytosine deamination may be present in the above list, although none of the samples showed a mutation burden and spectrum typical of heavily deaminated samples (E. Domingo, unpubl.)

SUPPLEMENTARY NOTE. INVESTIGATION OF THE POTENTIAL CAUSES OF LOSS OR GAIN OF DRIVER MUTATIONS.

Several cancers, especially good responders, showed loss of mutations in the major driver genes after therapy (Supplementary Figure 7). In a smaller number of cases, major driver mutations were gained. Although high levels of concordance with the high depth Ampliseq data were found (Supplementary Figures 4 and 11), we re-assessed in detail potential reasons for these observations: tumour purity, insufficient coverage, copy number/ploidy changes, and sampling effects/polyclonality.

1. Tumour purity

We investigated whether decreased tumour purity owing to treatment could explain our data. We started from the null that (i) the mutation complement of paired pre- and post-treatment samples was the same, (ii) sampling effects would cause loss or gain of some mutations, introducing noise but not directional bias, and (iii) apart from minor sampling differences, purity was the only factor that truly differed between pre- and post-treatment samples. We then used the exome sequencing data to derive a measure of hypothetical *relative* pre- *v* post-treatment sample purity based on the mutations present in each of those samples. Specifically, the relative purity of each tumour pair was estimated as the slope of the regression through the origin of post-treatment VAF on pre-treatment VAF. Two measures were used: in the first, a highly conservative estimate that took no account of sampling effects, mutations present in the pre-treatment sample but absent in the paired post-treatment sample were included; and in the second, only mutations present (at VAF >0.00) in both pre- and post-treatment samples were used. Copy number differences were not included, since overall ploidy estimates for the pre-and post-treatment samples of each cancer were very similar and the effects of individual changes would therefore be expected to be smoothed across the exome, reducing the strength of the correlation but having very little effect on its slope.

In fact, the regression was highly significant (typically P<0.001) in all cases. The slope of the regression line is shown in the chart below for each cancer: a value >1 indicates a gain in purity after treatment, and a value <1, a reduction. The purple bars represent the spread between the (lower) purity change estimate with all post-treatment mutations included and the (upper) estimate in which only post-treatment mutations with VAF >0 were included. Most cancers appeared to reduce in purity, especially responders, and a few had an estimated decrease to <20% of the pre-treatment purity even on the less stringent measure.

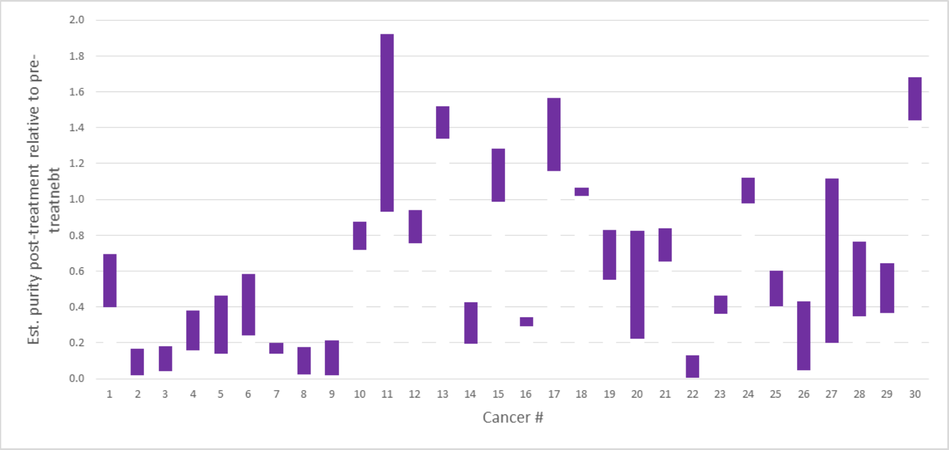

Estimated change in purity between pre- and post-treatment samples (<1 means reduced purity). The higher value of each bar represents the estimate using only mutations shared (at any VAF) between pre- and post-treatment samples, whereas the lower value represents the estimate when all pre-treatment mutations were included.

The following cancers were flagged as potentially showing purity reductions that could affect the accurate scoring of mutations: #2, #3, #8, #9, #22 and #26. However, it should also be noted that genetic bottlenecking can mimic reduced purity using the above method of assessment and, conversely, if the regression relies on a small number of mutations, it can fail to detect low purity. We therefore used two additional measures in an attempt to distinguish reduced purity from bottlenecking

- low estimated tumour cell content (<20% in histological sections)
- low mutation burden and low mutation VAF after therapy.

These metrics identified possible problems with the post-treatment samples of cancers #4, #20 and #29 in addition to #2, #3 and #26, and these 6 samples were excluded from the clonal evolution analysis (see diagram below and scatter plot).

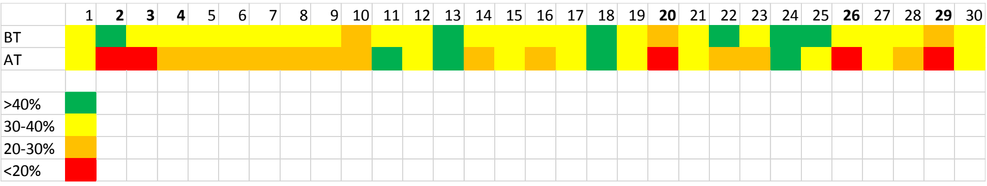

Estimated tumour purity in pre- (BT) and post-treatment samples (AT) from each patient. Excluded post-treatment cancers are in bold.

However, the post-treatment samples of cancers #8, #9 and #22 did not have problematic metrics, and we reasoned that their large estimated purity change from VAF regression was unlikely to be accurate. The post-treatment mutation burden in these samples (see below) was such that the VAF regression probably resulted from true clonal shifts. Alternative causes for the post-treatment mutation burden, such as infiltrating fibroblast or inflammatory cells, were highly unlikely to be responsible for the mutations detected, because those non-tumour cell populations were most unlikely to be clonal and hence would not produce detectable background somatic mutations [8]. The estimated severe reduction in purity of #8, #9 and #22 was thus most likely the result of bottleneck behaviour causing changes in clonal composition.

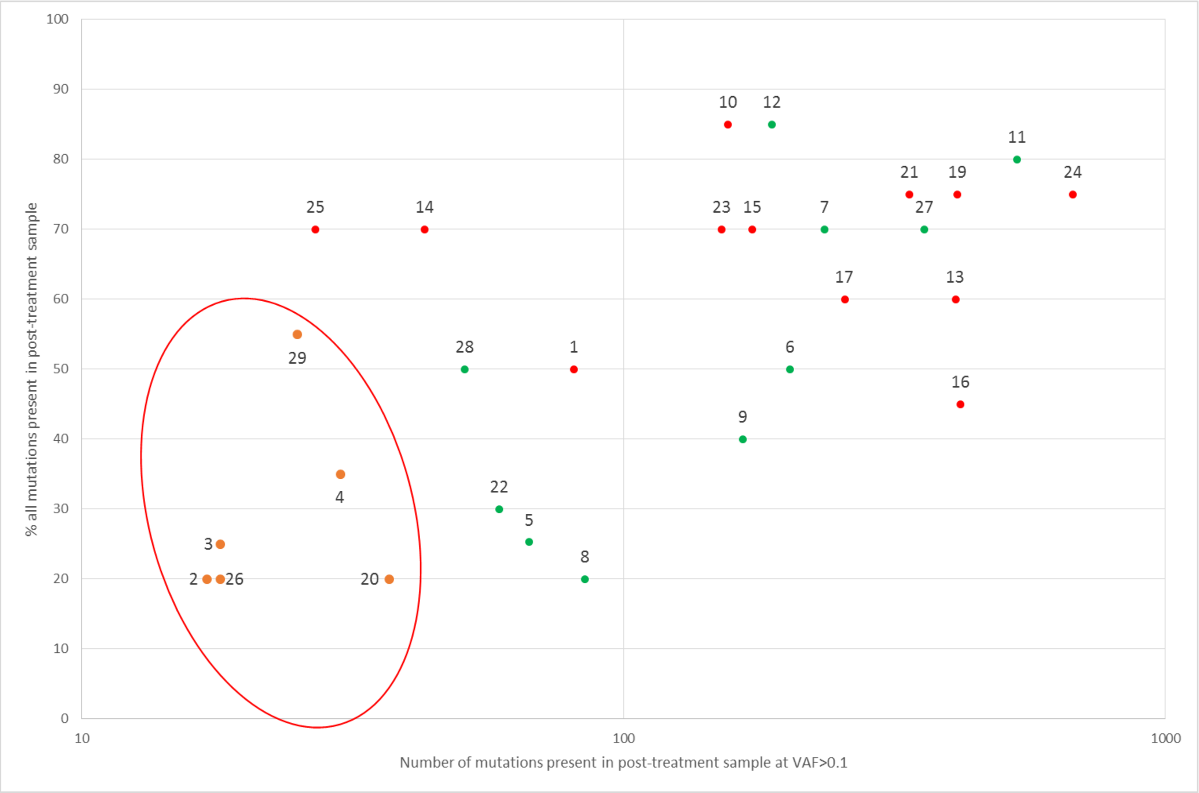

Plot illustrating post-treatment samples suspicious of low purity based on overall burden of mutations at moderate or greater frequency (VAF>0.1) in the post-treatment sample (x-axis) and/or the number of post-treatment mutations corrected for overall mutation burden in each cancer (y-axis). The 6 excluded cancers based on this assessment, plus histological estimation of tumour cell content, and estimated changes in VAF after treatment are shown in the oval. Cancers with bottleneck-like behaviour are shown as green data points. MSI+/polyclonal samples #18 and #30 are not shown owing their very large mutation burdens, but they did not show evidence of purity problems..

Although we excluded the 6 post-treatment cancer samples as a precaution, it was clear that they contained analysable cancer cells. SNP array-based copy number analysis for larger regions relies on tens of thousands of data points and hence is expected to be highly sensitive to SCNAs. Several large SCNAS were called by OncoSNP [9] in the post-treatment samples of cancers #3, #4, #20 and #29, with #4 also showing an *ERBB2* amplification: details are given in Supplementary Figure 6. The exclusion of cases #4 and #29 is

discussed further alongside their histology in Supplementary Figure 2. Further evidence that the post-treatment sample of excluded cancers contained analysable cancer cells came from mRNA expression profiling, in which these samples clustered with other pre- and post-treatment cancer samples and separately from normal tissue (details not shown).

2. *Insufficiently deep sequencing*

This is issue is evidently closely related to that of tumour purity. Let us consider the deep-coverage fresh-frozen DNA Ampliseq data, in which most EAC driver genes were sequenced to 1000-5000x coverage. Assume, conservatively, that a tumour is tetraploid (or the equivalent for a sub-clone), such that an SNV is present in only 25% of tumour reads, and also assume that the sample is composed of 90% contaminating normal cells. It follows that only 2.5% of all reads are mutant. Thus, in a total of 1000 reads, 25 reads are expected to be mutant if the library is evenly obtained from the input DNA. In our Ampliseq analysis, we have reported all driver mutations that have exceeded the standard cut-offs for calling (see Methods) in *any* pre- or post-treatment sample (exome, Ampliseq fresh-frozen, Ampliseq FFPE) from each patient. Thus for a mutation present at VAF ≥3% in a pre-treatment exome sequencing sample, we have reported the number of variant and reference alleles from the other samples, including the post-treatment fresh-frozen Ampliseq sample. If we regard 0.5% as the Ampliseq background mutation frequency, the chance of not finding ≥5 mutant reads (0.5% x 1000x) if the real VAF were 2.5% is $P=1.14x10^{-6}$ (exact binomial). Thus, except under conditions of extreme tumour impurity, considerable polyclonality or unequal allelic representation, we should detect SNVs under almost all situations. Below, we show the actual sequencing depth obtained for p53 in fresh-frozen samples from responders. Evidently, the depth achieved should in principle be enough to detect a very low proportion of p53-mutant cells close to background levels, suggesting that any issues were the result of purity problems rather than low sequencing depth.

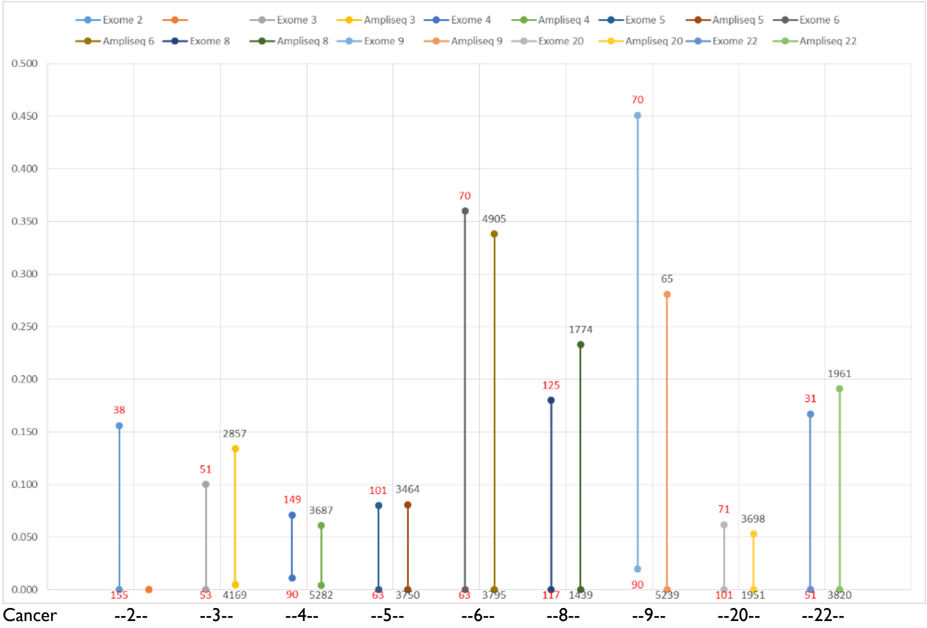

Plot illustrating p53 VAF changes from pre-treatment sample (upper value of each bar) to post-treatment sample (lower value of each bar). Exome sequencing data shown first followed by Ampliseq data for each cancer. The numbers show total read depth at that site.

Loss of a mutation after chemotherapy in the post-treatment sample is expected to be negatively associated with VAF if reduced purity and/or sampling are cause. In non-responders, this was indeed the case (see Figure below). By contrast, loss was largely independent of VAF in responders, a feature more typical of a bottleneck than purity decrease. Thus in the whole set of responders, these data provided confidence that our observations were not solely the result of purity problems, sampling or other technical issues (i.e. at least some mutation losses were biological in origin). However, whilst in principle, Ampliseq was sufficient to compensate even for tumour purity <<5% and the concordance between exome and Ampliseq data was overall very high, we acknowledged the possibility that Ampliseq VAF might not be linearly related to true VAF, and using this precautionary approach, we did not change our exclusion of 6 post-treatment samples.

Proportion of all variants present in pre-treatment samples that are lost from the paired post-treatment sample in responders (pink) and non-responders (black). All variants are at sites with minimum 60X read depth in both samples. The x-axis shows values using different minimum VAF cut-offs to select variants in the pre-treatment sample, thus taking into account possible sampling effects and contaminating non-neoplastic cells. At each cut-off, responders and non-responders differed significantly (P<0.00001).

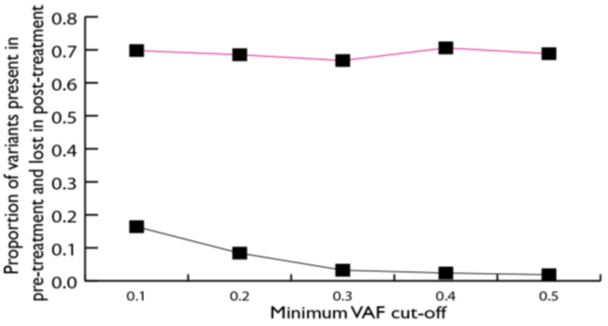

3. Copy number or ploidy changes

We searched for copy number changes as potential causes of loss (or gain) of driver mutations after therapy. We searched for concordance between a driver mutation loss or gain after treatment and a copy number change between the pre- and post-treatment sample at the site of the driver gene. Results are

Trio	PathR	Chr	Position	Gene	Mutation	Loss, or gain of mutation in post-treatment sample	Copy number change from pre- to post-treatment sample
1	1	9	139,401,093	NOTCH1	c.3902-2A>G	Loss	None
1	1	17	7,577,548	TP53	p.Gly245Ser/c.733G>A	Gain	Gain
1	1	17	7,578,190	TP53	p.Tyr220Cys/c.659A>G	Loss	Gain
3	1	17	7,577,565	TP53	p.Asn239Ser/c.716A>G	Loss	Loss
4	1	1	27,089,777	ARID1A	c.2732+1G>T	Loss	Loss
4	1	17	7,577,120	TP53	p.Arg273His/c.818G>A	Loss	Loss
5	1	3	178,936,082	PIK3CA	p.Glu542Lys/c.1624T>A	Loss	Gain
5	1	9	139,412,664	NOTCH1	p.Gly394Cys/c.1180G>T	Loss	Loss
5	1	17	7,577,082	TP53	p.Glu286Lys/c.856G>A	Loss	Loss
6	1	1	27,092,993	ARID1A	p.Leu975X/c.2924G>A	Gain	Gain
6	1	17	7,577,539	TP53	p.Arg248Trp/c.742C>T	Loss	None
8	1	1	27,105,825	ARID1A	p.Pro1813fs/c.5438delC	Loss	Loss
8	1	2	125,555,877	CNTNAP5	p.Val1065Ala/c.3194T>C	Loss	Loss
8	1	2	125,623,000	CNTNAP5	p.Glu1111Gly/c.3332A>G	Loss	Loss
8	1	17	7,577,114	TP53	p.Cys275Tyr/c.824G>A	Loss	Loss
8	1	18	48,591,918	SMAD4	p.Arg361Cys/c.1081C>T	Loss	Gain
17	0	4	153,247,289	FBXW7	p.Arg505Ser/c.1513C>A	Gain	Loss
17	0	19	11,135,079	SMARCA4	p.Val1016Met/c.3046G>A	Gain	Gain
18	0	17	7,578,457	TP53	p.Arg158His/c.473G>A	Loss	None
20	1	9	139,401,780	NOTCH1	p.Cys1207Phe/c.3620G>T	Loss	Loss
20	1	13	66,878,846	PCDH9	p.Leu1185Met/c.3553C>A	Loss	Gain
20	1	17	7,578,413	TP53	p.Val173Leu/c.517G>T	Loss	None
21	0	3	178,936,082	PIK3CA	p.Glu542Lys/c.1624T>A	Gain	Gain
22	1	1	27,101,273	ARID1A	p.Gln1519*/c.4555C>T	Loss	Loss
22	1	3	178,936,082	PIK3CA	p.Glu542Lys/c.1624T>A	Loss	Loss
22	1	9	139,409,067	NOTCH1	p.Thr701Ile/c.2102C>T	Loss	Loss
22	1	17	7,577,022	TP53	p.Arg306*/c.916C>T	Loss	Loss
27	1	2	125,555,816	CNTNAP5	p.Val1045Leu/c.3133G>T	Gain	None
27	1	17	7,578,190	TP53	p.Tyr220Cys/c.659A>G	Gain	Gain
28	1	9	139,410,010	NOTCH1	p.Gln610Ter/c.1829G>A	Loss	Loss
28	1	9	139,412,239	NOTCH1	p.Asp469Gly/c.1406A>G	Loss	Loss
28	1	9	139,412,239	NOTCH1	p.Asp469Gly/c.1406A>G	Loss	Loss
30	0	2	125,671,851	CNTNAP5	p.Glu1303Lys/c.3907G>A	Loss	Loss
30	0	3	178,916,910	PIK3CA	p.Leu99Phe/c.297A>T	Loss	None
30	0	17	7,577,121	TP53	p.Arg273Cys/c.817C>T	Loss	None

shown from the 23 paired fresh-frozen samples with SNP array SCNA/LOH data. Sequence data are derived from exome sequencing and Ampliseq. Given that we found no significant change in ploidy between pre- and post-treatment samples (Supplementary Figure 6), we expected that SCNA gains and losses after treatment should be equally likely at any locus. We then tested whether there was concordance between SNV loss or gain and copy number change at that locus. Assuming that "no copy number change" was always discordant with an SNV change, 25/35 mutations showed concordant copy number and SNV change (P=0.017, exact binomial). Although we were unable to phase our SNP alleles and SNVs, these data suggest that copy number changes can account for some, but not all, examples of mutation loss or gain after treatment.

4. Sampling effects and polyclonality

Sampling effects inevitably cause loss and gain of mutations, especially if the evolution of EACs is highly branched (Supplementary Figure 9) with parallel or convergent evolution, as was shown by several cancers in this study. This factor may in part be avoidable by the use of very deep sequencing, but is hard to overcome entirely and can, for example, lead to over-estimation of the change in purity after treatment.

SUPPLEMENTARY REFERENCES

1. Kim H*, et al.* Whole-genome and multisector exome sequencing of primary and post-treatment glioblastoma reveals patterns of tumor evolution. *Genome research* **25**, 316-327 (2015).

2. Dulak AM*, et al.* Exome and whole-genome sequencing of esophageal adenocarcinoma identifies recurrent driver events and mutational complexity. *Nature genetics* **45**, 478-486 (2013).

3. Gebski V, Burmeister B, Smithers BM, Foo K, Zalcberg J, Simes J. Survival benefits from neoadjuvant chemoradiotherapy or chemotherapy in oesophageal carcinoma: a meta-analysis. *The Lancet Oncology* **8**, 226-234 (2007).

4. Mandard AM*, et al.* Pathologic assessment of tumor regression after preoperative chemoradiotherapy of esophageal carcinoma. Clinicopathologic correlations. *Cancer* **73**, 2680-2686 (1994).

5. Rouvelas I, Zeng W, Lindblad M, Viklund P, Ye W, Lagergren J. Survival after surgery for oesophageal cancer: a population-based study. *The Lancet Oncology* **6**, 864-870 (2005).

6. Jamieson GG, Mathew G, Ludemann R, Wayman J, Myers JC, Devitt PG. Postoperative mortality following oesophagectomy and problems in reporting its rate. *The British journal of surgery* **91**, 943-947 (2004).

7. Dulak AM*, et al.* Gastrointestinal adenocarcinomas of the esophagus, stomach, and colon exhibit distinct patterns of genome instability and oncogenesis. *Cancer research* **72**, 4383-4393 (2012).

8. Gerlinger M*, et al.* Ultra-deep T cell receptor sequencing reveals the complexity and intratumour heterogeneity of T cell clones in renal cell carcinomas. *The Journal of pathology* **231**, 424-432 (2013).

9. Knight SJ*, et al.* Quantification of subclonal distributions of recurrent genomic aberrations in paired pre-treatment and relapse samples from patients with B-cell chronic lymphocytic leukemia. *Leukemia* **26**, 1564-1575 (2012).
